# Identifying the earliest citrus responses to *Candidatus* Liberibacter asiaticus infection: a temporal metabolomics study

**DOI:** 10.3389/fpls.2024.1455344

**Published:** 2024-11-06

**Authors:** Jingwen Li, Yuanzhi Zimmy Wang, Fred G. Gmitter, Yu Wang

**Affiliations:** Citrus Research and Education Center, Institute of Food and Agricultural Sciences, University of Florida, Lake Alfred, FL, United States

**Keywords:** Huanglongbing (HLB), temporal metabolomics, early biomarkers, machine learning, citrus

## Abstract

The global citrus industry faces a great threat from Huanglongbing (HLB), a destructive disease caused by ‘*Candidatus* Liberibacter asiaticus’ (*C*Las) that induces significant economic losses without any known cure. Understanding how citrus plants defend against HLB, particularly at the early stages of infection, is crucial for developing long-term solutions. This study investigated the earliest metabolic responses of fresh citrus leaves to *C*Las infection using untargeted metabolomics and machine learning models. HLB-tolerant and HLB-sensitive cultivars were compared to analyze their biochemical reactions within 48 hours post-infection. HESI/Q-Orbitrap MS analysis identified temporal differential metabolites, revealing distinct metabolic pathways activated in response to *C*Las infection. Both cultivars responded by increasing specific metabolite concentrations, such as flavonoids, within 2 hours post-infection, but the HLB-tolerant cultivar maintained higher levels throughout the 48-hour period. This early metabolic activity could influence long-term plant health by enhancing disease resistance and reducing pathogen impact. These findings provide potential biomarkers for breeding HLB-resistant cultivars and offer valuable insights for developing sustainable management strategies to mitigate the impact of HLB on the citrus industry, ensuring its long-term productivity and economic viability.

## Graphical Abstract

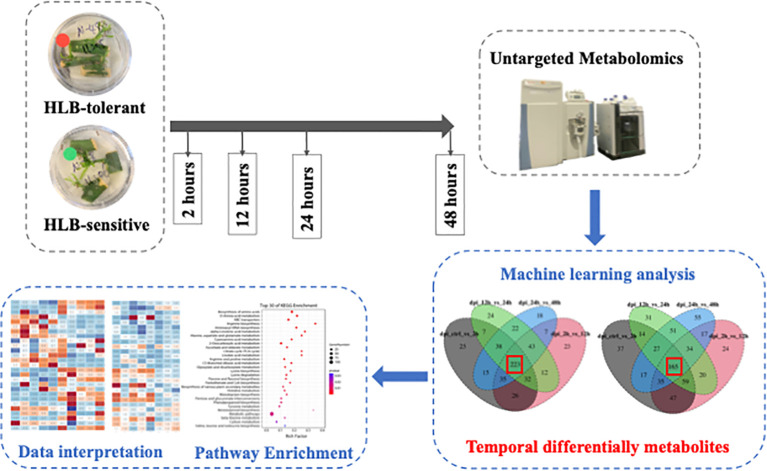

## 1 Introduction

Citrus greening disease, or citrus HLB, stands as one of the most catastrophic diseases for the global citrus industry, causing substantial economic losses. This infectious disease is attributed to the phloem-restricted *Candidatus* Liberibacter spp., with the Asian species *C*Las being particularly significant. They are mainly transmitted in the field through phloem sap-feeding insect vectors, psyllids (Hemiptera: Psylloidea), such as *Diaphorina citri* Kuwayama (Asiatic citrus psyllid), *Trioza erytreae* Del *Guerci* (African citrus psyllid), and *Cacopsylla citrisuga* Yang & Li (Cen et al., 2012). In the Americas and Asia, *C*Las serves as the principal etiological agent, while *D. citri* functions as the primary vector. Observations indicate that *D. citri* vectors possess a considerable capacity for movement, facilitating the dissemination of *C*Las over extensive distances in newly introduced regions (Carmo-Sousa et al., 2020).

The symptoms of HLB may not always be readily apparent or easily discernible. During the early stages of infection, symptoms could be subtle and easily overlooked (Pandey and Wang, 2019). However, as the disease progresses, more noticeable symptoms may appear. Physiologically, affected citrus trees display blotchy mottle leaves, produce multiple off-season flowers, exhibit yellow shoots, and show partial greening of the fruit peel. The disease also leads to root decay, tree dwarfing, and significant yield reduction (Li et al., 2019). Anatomically, HLB causes phloem necrosis, plugging, and collapse, which disrupt the transport of photoassimilates and result in thickened and distorted cell walls. This phloem malfunction potentially leads to excessive starch accumulation, which is one of the typical symptoms of HLB disease (Suh et al., 2021). Moreover, HLB-affected fruits often have bitter or off-flavor traits due to higher acid, flavonoids, limonin, and nomilin content and lower sugar content, which seriously affects the fruit quality and commercial value. As the disease advances, trees typically experience a decline within a few years of the infection’s onset, potentially leading to their death and resulting in significant economic losses for the growers (Nehela and Killiny, 2020).

HLB infects most of the citrus species, hybrids, cultivars, and some citrus relatives, especially the existing commercial citrus varieties, such as “Murcott” mandarin (C. reticulata Blanco) (Suh et al., 2021) and “Valencia” sweet orange (*C. sinensis* (L.) Osbeck) (Folimonova et al., 2009). A few commercial varieties, including Persian lime (*C. latifolia*) and “LB8-9” Sugar Belle^®^ mandarin hybrid (*C. reticulata* “Clementine” mandarin × “Minneola” tangelo), have shown evident tolerance to HLB (Folimonova et al., 2009). Interestingly, it was reported that HLB-tolerated cultivars (e.g., Sugar Belle^®^ mandarin), in comparison to HLB-sensitive cultivars (e.g., “Valencia” sweet orange), may have superior internal structural preservation and more vital phloem regeneration ability, which compensate for the dysfunctional phloem (Deng et al., 2019). HLB poses a significant challenge to the worldwide citrus industry, and currently, there is no cure. Approaches like using antibiotics, insecticides, foliar nutrients, plant defense activators, thermotherapy, and controlling vector psyllids have their own limitations and can only serve as a short-term solution (Munir et al., 2021). In this way, it is crucial to find sustainable and viable alternative approaches for managing citrus HLB in field settings, particularly breeding tolerant citrus cultivars that can survive the HLB.

As mentioned above, even though HLB-affected plants exhibit visible symptoms in the later stages of the disease, HLB-affected trees do not display typical symptoms during the early stages. Detecting HLB at this pre-symptomatic stage is crucial for implementing effective intervention strategies to curb the spread of this devastating disease by psyllids (Sanchez et al., 2020). Several studies have focused on early detection of HLB using Raman spectroscopy or transcriptomics (Zheng and Zhao, 2013; Sanchez et al., 2019; Wang et al., 2019; Sanchez et al., 2020). But limited research has investigated HLB detection as early as 48 hours of pathogen infection by examining transcriptional or metabolic changes. Moreover, the mechanisms underlying the varying tolerance of different citrus species to *C*Las infection remain unclear. Studying the early response of HLB-tolerant citrus plants to *C*Las can not only provide insights into their earliest defense mechanisms but also facilitate the breeding of disease-tolerant or resistant cultivars. Therefore, it is crucial to investigate the metabolic mechanisms associated with the initial stages of HLB-tolerant citrus leaf responses after *C*Las infection.

In this study, the citrus leaf metabolic reactions to *C*Las during the 48-hours post-infection (hpi) were investigated. To obtain the fresh citrus leaves, the cuttings from two citrus cultivars, namely the HLB-tolerant ‘LB8-9’ Sugar Belle^®^ mandarin and the HLB-sensitive ‘Valencia’ sweet orange (clone 1-14-19), were collected and cultivated in a nutrient medium. Within 12 to 20 days, new fresh citrus shoots and leaves emerged. Subsequently, three feeding conditions are applied to these fresh leaves: control (no psyllid feedings), *C*Las-infected psyllid feedings, and *C*Las-free psyllid feedings. Fresh leaf samples are collected at 2-, 12-, 24-, and 48 hpi for each feeding condition. These leaf samples underwent untargeted metabolomic analysis to study the metabolic biomarkers associated with the citrus leaf response to *C*Las infection at the earliest stages. HESI/Q-Orbitrap MS analysis was used to examine metabolites in the fresh leaves, and machine learning models were employed to identify temporal differential metabolites during the 48-hour post-infection. Based on the enrichment analysis of temporal differential metabolites, the metabolic responses along the pathways associated with HLB defense of the two cultivars were extensively examined to uncover the potential underlying secrets behind the HLB tolerance of the HLB-tolerant cultivar. These findings held great promise in providing valuable insights for the development of more tolerant cultivars, ultimately serving as a sustainable, long-term solution for controlling citrus HLB.

## 2 Materials and methods

### 2.1 Materials

To prepare the *C*Las-free or *C*Las-infected *D. citri* adults for later feeding experiments, potential *C*Las-infected *D. citri* adults were collected from HLB-affected sweet orange plants (*C. sinensis*) in the greenhouse at Citrus Research and Education Center (Lake Alfred, FL), and maintained on *C*Las-infected young plants (*C. sinensis*) for several generations in the psyllid house. These psyllids were confirmed to be 87% infected with *C*Las (Pandey et al., 2021). Psyllid house conditions were 25°C, 65% r.h., and L14:D10 h photoperiod. The *C*Las-free *D. citri* adults were maintained on Curry tree (*Murraya koenigii*) and transferred to *C*Las-free young plants (*C. sinensis*) before the feeding experiments. The *D. citri* adults were confirmed as *C*Las-free or *C*Las-infected by using quantitative real-time polymerase chain reaction (qPCR) before the feeding experiments.

To prepare fresh (newly generated) leaves for later feeding experiments, *C*Las-free budwoods (scion woods) of HLB-sensitive ‘1-14-19’ Valencia (*Citrus sinensis* ‘Valencia’) and HLB-tolerant ‘LB8-9’ Sugar Belle^®^ (*C. reticulata* “Clementine” mandarin *×* “Minneola” tangelo) were obtained from greenhouse-grown, certified pathogen-tested trees from Brite Leaf Citrus Nursery, LLC (Lake Panasoffkee, FL). The budwoods were washed twice with soapy water and rinsed with DI water. Then the budwoods were cut into 1~2 cm budwood cuttings and maintained with one bud eye each. The cuttings were cleaned with bleach water and sterilized by soaking in 75% ethanol for 1min. Then the sterilized cuttings were rinsed in sterilized DI water for 5-10min and dried on sterilized plates in the air. Five cuttings were placed in one petri dish with nutritional media which consisted of 0.7% (w/v) phytagel and 2% (w/v) sucrose, adjusted to pH 5.8 with KOH. The cuttings were allowed to grow for 12~20 days to get the fresh leaves from sprouting axillary buds. Finally, the cuttings with new fresh leaves were selected out, sterilized, and used for feeding experiments.

**Figure 1:**
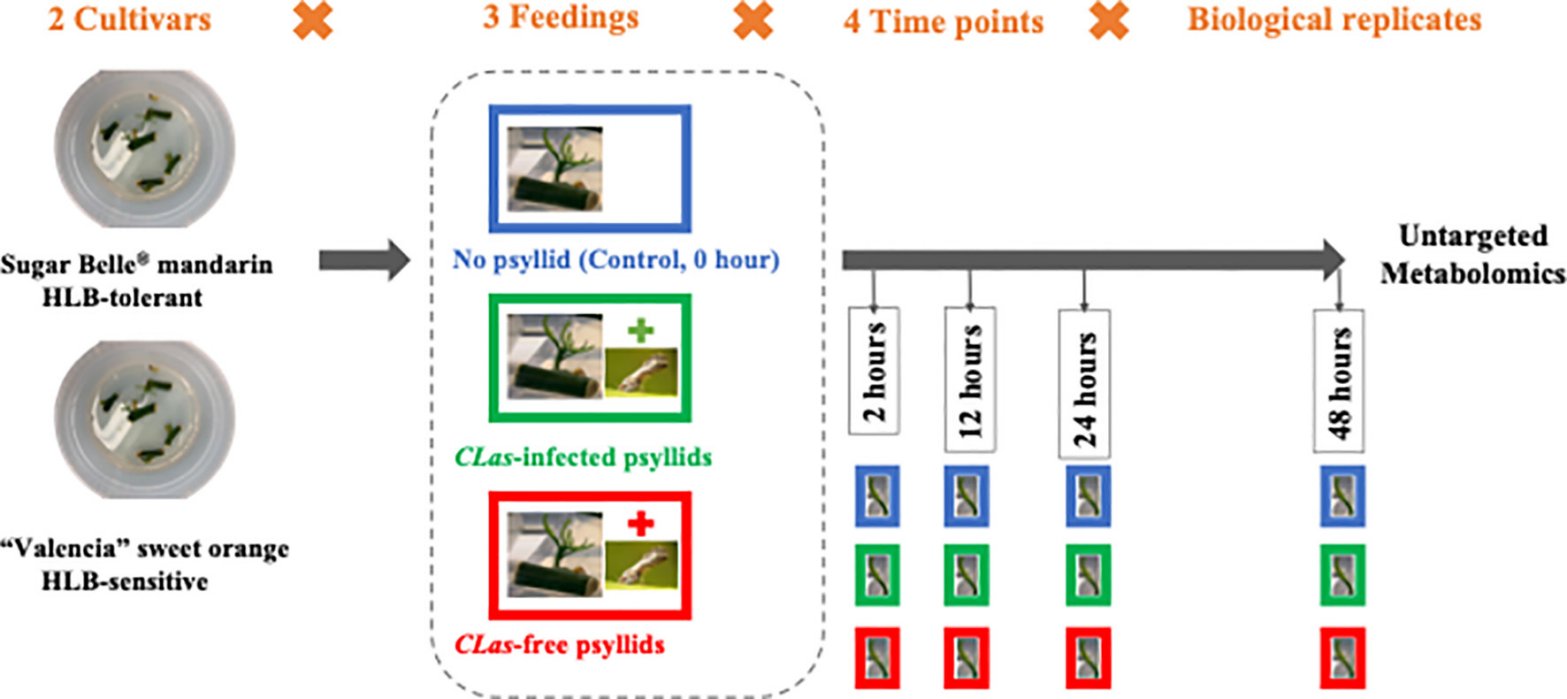
Scheme of feeding experiments.

During the feeding experiments, three cuttings with several leaves attached and six to eight *D. citri* adults constituted a test. As shown in **Figure 1**, at four time points (2, 12, 24, 48 hpi), the fresh leaves were collected after different feeding treatments (‘No psyllid’, ‘*C*Las-free psyllid feedings’, ‘*C*Las-infected psyllid feedings’). The collected samples were promptly frozen in liquid nitrogen and stored at −80°C until analysis. As ‘No psyllid’ can be considered as 0 hpi for both ‘*C*La*s*-free psyllid’ and ‘*C*Las-infected psyllid’ feedings, this experiment can provide the fresh leaves from four treatments: Sugar Belle^®^ with *C*Las-free psyllid feedings (SB_*C*Las-free), Sugar Belle^®^ with *CLas*-infected psyllid feedings (SB_*C*Las-infected), ‘Valencia’ with *CLas*-free psyllid feedings (Val_*C*Las-free), ‘Valencia’ with *C*Las-infected psyllid feedings (Val_*C*Las-infected).

### 2.2 Chemicals and reagents

Authentic chemicals serving as internal standards, including hippuric acid-d_5_, salicylic acid-d_6_, genistein-d_4_, D-sorbitol-^13^C_6_ were purchased from Sigma-Aldrich (St. Louis, MO, USA). Acetonitrile, water, methanol, and formic acid of LC-MS grade were ordered from Fisher Scientific (Fair Lawn, NJ, USA).

### 2.3 Sample extraction

The citrus fresh leaf tissues of 25mg were weighted and subsequently placed into two 2 mL tubes with ceramic beads. Then 240 μl 70% Methanol and 10uL of internal standards mixture (hippuric acid-d_5_, salicylic acid-d_6_, genistein-d_4_, D-sorbitol-^13^C_6_, 5ppm) were added into the tubes. The mixture samples underwent vortex homogenization for 60 minutes, followed by extraction through sonication for 30 minutes. The extract was collected using disposable syringes and then filtered through a 0.22 um filter to remove any impurities or particles, and 5 μl of each extract was archived to mix and make QC samples. Seven to nine biological replicates were used for each time point within each treatment. Those samples were prepared for untargeted metabolomics and each sample was analyzed twice by the instrument (technical duplicates).

### 2.4 UHPLC-HESI/Q-Orbitrap MS analysis

The metabolites profiling was finished by Q Exactive Plus mass spectrometer (Thermo Fisher Scientific, Waltham, MA, USA) coupled to the Ultrahigh Performance Liquid Chromatography (UHPLC) by a Heated Electrospray Ionization (HESI) interface. UPLC separation was performed using a Thermo Vanquish Flex Binary RSLC platform (Thermo Fisher Scientific). Nonpolar or hydrophobic compounds were chromatographically separated using a reverse-phase Agilent Poroshell HPH-C18 column (2.1×150 mm, 1.9 μm) at a column temperature of 40°C. The mobile phases were 0.1% formic acid water (A) and 0.1% formic acid ACN (B). The gradient program was: 0-28 min, 2-100% B; 28-30 min, 100% B; 30-32min, 100 -2% B; 32-37 min, 2% B. The flow rate was constant at 0.4 mL/min and the injection volume was 3 μL. The optimized HESI conditions were: the capillary temperature at 320°C, vaporizer temperature at 250°C, aux gas heater temperature at 350°C, aux gas flow rate at 0 arb, sheath gas flow rate at 20 arb, sweep gas flow rate at 0 arb, ion spray voltage at 3.5 kV, and S-lens RF level, 55%. Full MS/ddMS2 scan mode was employed in both positive and negative ion modes. In Full MS scan mode, mass spectra were collected within the m/z range of 70-1,050 at a resolution of 140,000, with automated gain control (AGC) set at 3 × 10^6^ and a maximum ion accumulation time (IT) of 600 ms. For ddMS2 scan mode, stepped normalized collision energy (NCE) was applied at 20, 40, and 60 V, maintaining a resolution of 17,500. The m/z range was identical to that of the Full MS scan mode. AGC was set at 1 × 10^5^, with an IT of 50 ms. Data-dependent scanning was employed to trigger second-stage fragmentation, selecting the 12 most robust parent ions (TopN) at each MS scanning point for further MS^2^ fragmentation. The dynamic exclusion function was implemented to optimize analysis time by preventing repetitive ion scans.

Data acquisition and analysis were conducted using Xcaliber software (Thermo Fisher Scientific). Three biological replicates were acquired, and Compound Discoverer 3.1 software (Thermo Fisher Scientific) was utilized for metabolite identification and structural elucidation. Raw data features were filtered based on RSDs of QC samples below 20%, availability of molecular formula, potential compound name, spectrum of MS2 fragmentation, and low mass error (< 5 ppm). Tentative identification of these features was achieved by using the MS^2^ fragmentation pattern (FISh) and in-built MS databases such as mzCloud and KEGG.

### 2.5 Statistical analysis

Principal component analysis (PCA) was executed utilizing MetaboAnalyst (https://www.metaboanalyst.ca/) to assess the clustering patterns within the metabolomics datasets. Prior to analysis, all datasets underwent preprocessing through sum scaling and Z-score normalization. The identification of potential outliers was determined by applying a threshold of a 95% confidence interval (CI).

### 2.6 Machine learning analysis

This study utilized machine learning analysis for the identification of differentially expressed metabolites. Given the high dimensionality and continuity of the data, six supervised machine learning classifiers from Python’s Scikit-learn package v1.1.3 (Python Software Foundation, v3.9.3) were chosen and applied to two datasets in pairs. These classifiers included Random Forest (RF), Gradient Boosting (GB), Logistic Regression (LR)-L1, LR-L2, Support Vector Machine (SVM), Multi-layer Perceptron (MLP) (Pedregosa et al., 2011). The utilized configurations for the six machine learning classifiers are detailed in **Supplementary Table S1**. To assess the performance of each model and mitigate overfitting, a k-fold (k = 6) cross-validation was implemented. Testing accuracies were subsequently reported as the average accuracies derived from the 6-fold cross-validation. The identification of differentially expressed metabolites involved the screening of important features based on the rank of average important coefficients for each feature derived from the 6-fold cross-validation.

### 2.7 Screening of temporal biomarker metabolites

To find the differentially expressed metabolites that changed with time after *C*Las infection, the metabolic intensity from two adjacent hpi time points (e.g., e.g., 0 hpi vs 2 hpi, 2 hpi vs 12 hpi, 12 hpi vs 24 hpi, 24 hpi vs 48 hpi) were compared. The features with high importance coefficients in each comparison are retained. As a result, the machine learning classifications were repeated four times and four sets of important features obtained from five time points for both SB_*C*La*s*-infected and Val_*C*Las-infected treatments. Then, the intersection of these four feature sets was taken to identify the temporal differential metabolites, which were the features that exhibit changes across all five time points. The tentative identification of these temporal differential metabolites was achieved through the utilization of the MS^2^ fragmentation pattern (FISh) and in-built MS databases, including mzCloud and KEGG.

### 2.8 KEGG pathway enrichment analysis

Temporal differential metabolites were subjected to KEGG enrichment analyses using the Omicshare online software (https://www.omicshare.com/), aiming to identify metabolic pathways associated with plant tolerance or resistance to HLB at a significance threshold of p < 0.05. The meaningful pathways were selected and the metabolites on these pathways were further discussed. The heatmap plot of these metabolites was performed using the OmicStudio tools at https://www.omicstudio.cn.

## 3 Results

### 3.1 Metabolic variations of fresh citrus leaves within 48 hpi

**Figure 2:**
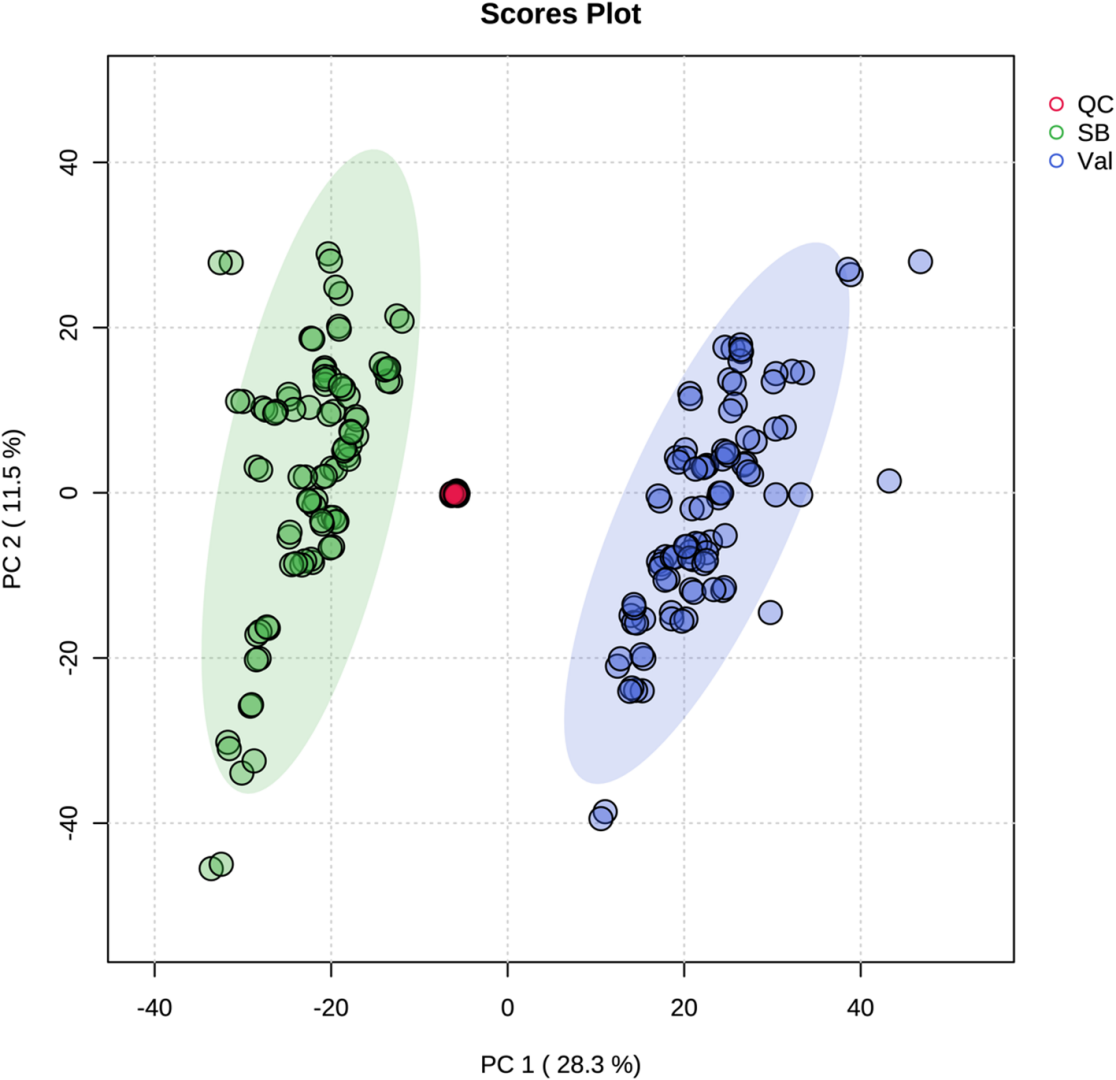
PCA plot of untargeted metabolites in all samples.

Metabolic responses at 0, 2, 12, 24, and 48 hpi after *C*Las infection (**Figure 1**) were examined in fresh leaves of both HLB-tolerant and HLB-sensitive cultivars utilizing an untargeted LC-MS/MS approach. Following the application of stringent filtering criteria, including restrictions on relative standard deviation (RSD) (< 20%), mass error (< 5 ppm), identification of molecular formulas, potential annotations, and the availability of MS2 spectra, a comprehensive set of 1768 metabolic features was successfully identified, with 844 features in the negative ionization mode and 924 in the positive ionization mode. The obtained features were then used for PCA analysis to explore the variations in metabolic difference between the HLB-tolerant and HLB-sensitive cultivars. In **Figure 2**, the closely clustered quality control (QC) samples exhibited an excellent reproducibility of the sample preparation and instrumental analysis. And it was evident that the samples from HLB-tolerant and HLB-sensitive cultivars (including both *C*Las-free and *C*Las-infected treatments) are distinctly separated into two distinct groups. This result implied that the inherent differences between cultivars had a substantial impact on plant metabolism, aligning with our previous study’s conclusions (Suh et al., 2021). These inherent metabolite disparities could contribute to the tolerance of ‘LB8-9’ Sugar Belle^®^ cultivar while ‘Valencia’ is more susceptible to HLB disease.

**Figure 3:**
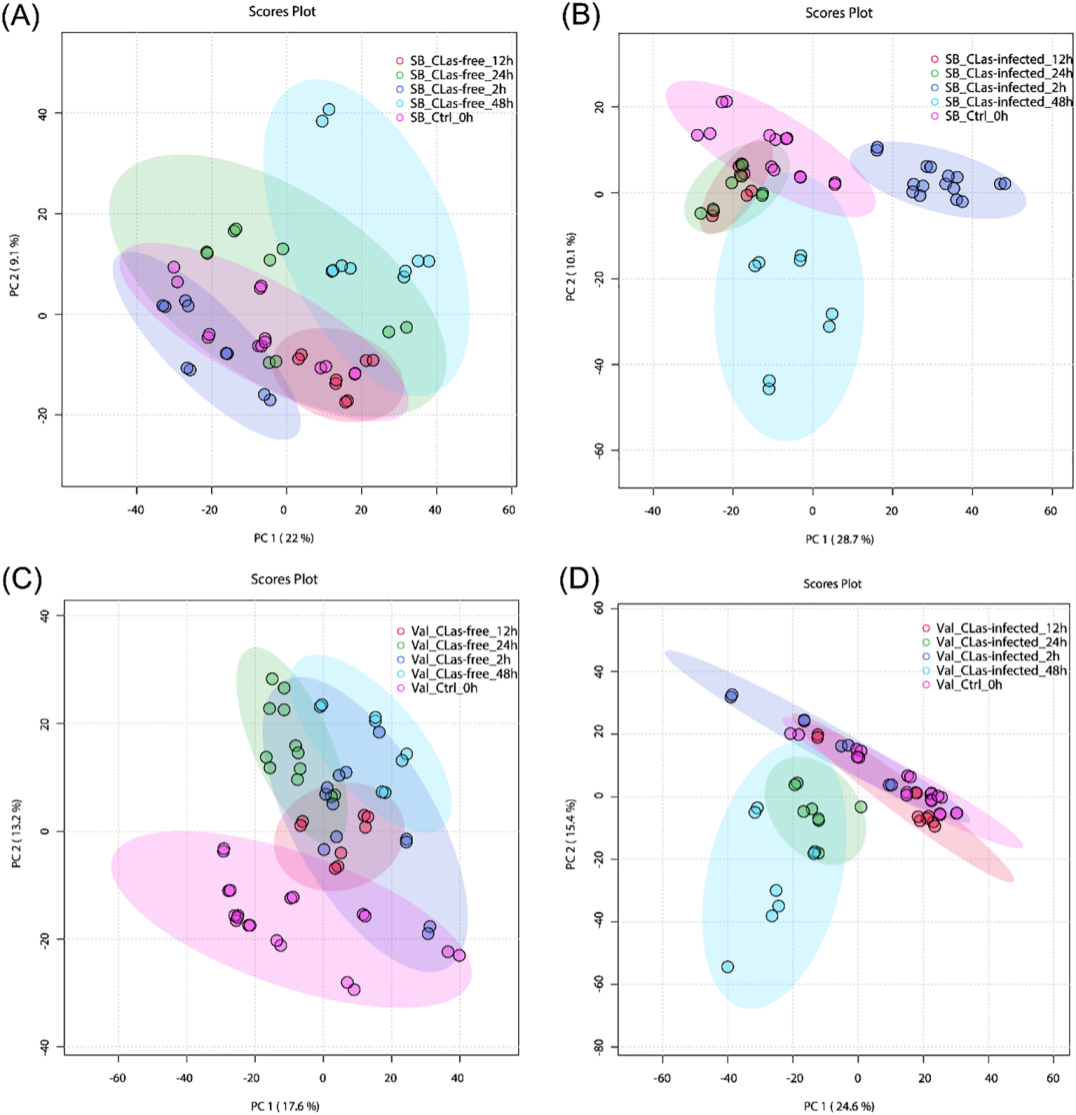
PCA plots for untargeted metabolites in leaves under different treatments. **(A)** SB_*C*Las-free treatment. **(B)** SB_*C*Las-infected treatment. **(C)** Val_*C*Las-free treatment. **(D)** Val_*C*Las-infected treatment.

To gain insights into the temporal metabolic responses of citrus plants to *C*Las infection, additional PCA analyses were conducted. This analysis aimed to differentiate metabolite responses at different time points (0-, 2-, 12-, 24-, and 48 hpi) for four treatments: SB_*C*Las-free, SB_*C*Las-infected, Val_*C*Las-free, and Val_*C*Las-infected. **Figure 3A** illustrates the results of the PCA analysis for SB_*C*Las-free treatment, revealing a relatively high degree of overlap between clusters representing different time points (0-, 2-, 12-, 24-, and 48 hpi). This overlapping indicated a strong similarity between the corresponding groups, suggesting that the HLB-tolerant cultivar exhibited minimal metabolic responses to psyllid feeding behaviors. However, a decrease in the degree of overlap between clusters was observed in SB_*C*Las-infected treatment as shown in **Figure 3B**, particularly noticeable between the 2 hpi cluster and the other clusters. This decrease indicated that the HLB-tolerant cultivar underwent noticeable metabolic changes during 2 hpi time in response to *C*Las infection.

In **Figure 3C**, the clusters at 24 hpi and 48 hpi in the Val_*C*Las-free treatment were completely separated from the 0 hpi cluster but showed overlap with the 2 hpi and 12 hpi clusters. This separation indicated that the HLB-sensitive cultivar exhibited perceptible metabolic changes within 48 hours after psyllid feeding. It suggested that the HLB-sensitive cultivar was more sensitive to psyllid feeding behaviors compared to the HLB-tolerant cultivar. Furthermore, in the Val_*C*Las-infected treatment (**Figure 3D**), the metabolic changes at 24 hpi and 48 hpi were even more pronounced, with no overlaps observed with the 2 hpi and 12 hpi clusters. This finding revealed that the presence of *C*Las during feeding activities heightened the plant’s reactions to psyllid feeding. In short, both the HLB-tolerant and HLB-sensitive cultivars exhibited temporal metabolic changes in response to *C*Las infection.

The results indicate that there are inherent metabolic differences between the HLB-tolerant and HLB-sensitive cultivars, and these two cultivars also exhibit distinct metabolic responses to psyllid feeding activities. To accurately identify the initial metabolic responses caused by *C*Las infection and uncover the true potential defense mechanisms of the two cultivars within 48hpi, it is essential to account for and control these pre-existing differences. Therefore, to reveal the true differences of metabolic activities caused by *C*Las infection, this study proposes the use of “relative intensity” in the discussion part, which is intensity ratios of metabolites in *C*Las-infected samples compared to *C*Las-free samples.

### 3.2 Temporal metabolic changes based on machine learning analysis

To further investigate the temporal changes of metabolites in *C*Las-infected samples, a screening process was implemented to identify differentially expressed metabolites at different hpi time points. This process compared the 1768 metabolic features across adjacent time points following *C*Las infection, specifically between 0 hpi and 2 hpi, 2 hpi and 12 hpi, 12 hpi and 24 hpi, and 24 hpi and 48 hpi. The comparison was performed using machine learning analysis, employing various classifiers such as Random Forest (RF), Gradient Boosting (GB), Logistic Regression (LR)-L1, LR-L2, Support Vector Machine (SVM), and Multi-layer Perceptron (MLP). **Supplementary Table S2** presented the testing accuracies of all machine learning methods. Notably, SVM consistently outperformed other classifiers across multiple evaluation metrics, demonstrating its effectiveness for the classification tasks at hand. Subsequently, the metabolic features with top 400 importance coefficients from each SVM model were selected as the differentially expressed metabolites. This selection process was carried out separately for four comparisons, resulting in four distinct sets of differentially expressed metabolites for both SB_*C*Las-infected and Val_*C*Las-infected treatments. The next step involved dividing these four sets to identify temporal differential metabolites that exhibited changes in response to *C*Las infection from 0 to 48 hpi. As illustrated in **Supplementary Figure S1**, a total of 223 temporal differential metabolites were identified for SB_*C*Las-infected treatment, while 165 were identified for Val_*C*Las-infected treatment. These temporal differential metabolites (listed in **Supplementary Table S3**) were combined and utilized for further enrichment analysis.

### 3.3 Discovered metabolic pathways with temporal changes within 48 hpi

Temporal differential metabolites acquired through untargeted metabolomics were employed for the identification of Kyoto Encyclopedia of Genes and Genomes (KEGG) pathways regulated within the 48-hour timeframe following *C*Las infection. These temporal differential metabolites were mapped into thirty-one KEGGc pathways (listed in **Supplementary Table S4**), most of which were related to carbohydrate metabolism, amino acid metabolism, and biosynthesis of other secondary metabolites. The other three pathways are energy metabolism, metabolism of cofactors and vitamins, and translation. Among them, the most dominant three pathways were arginine biosynthesis, benzoxazinoid biosynthesis, and alpha-linolenic acid metabolism.

## 4 Discussion

**Figure 4:**
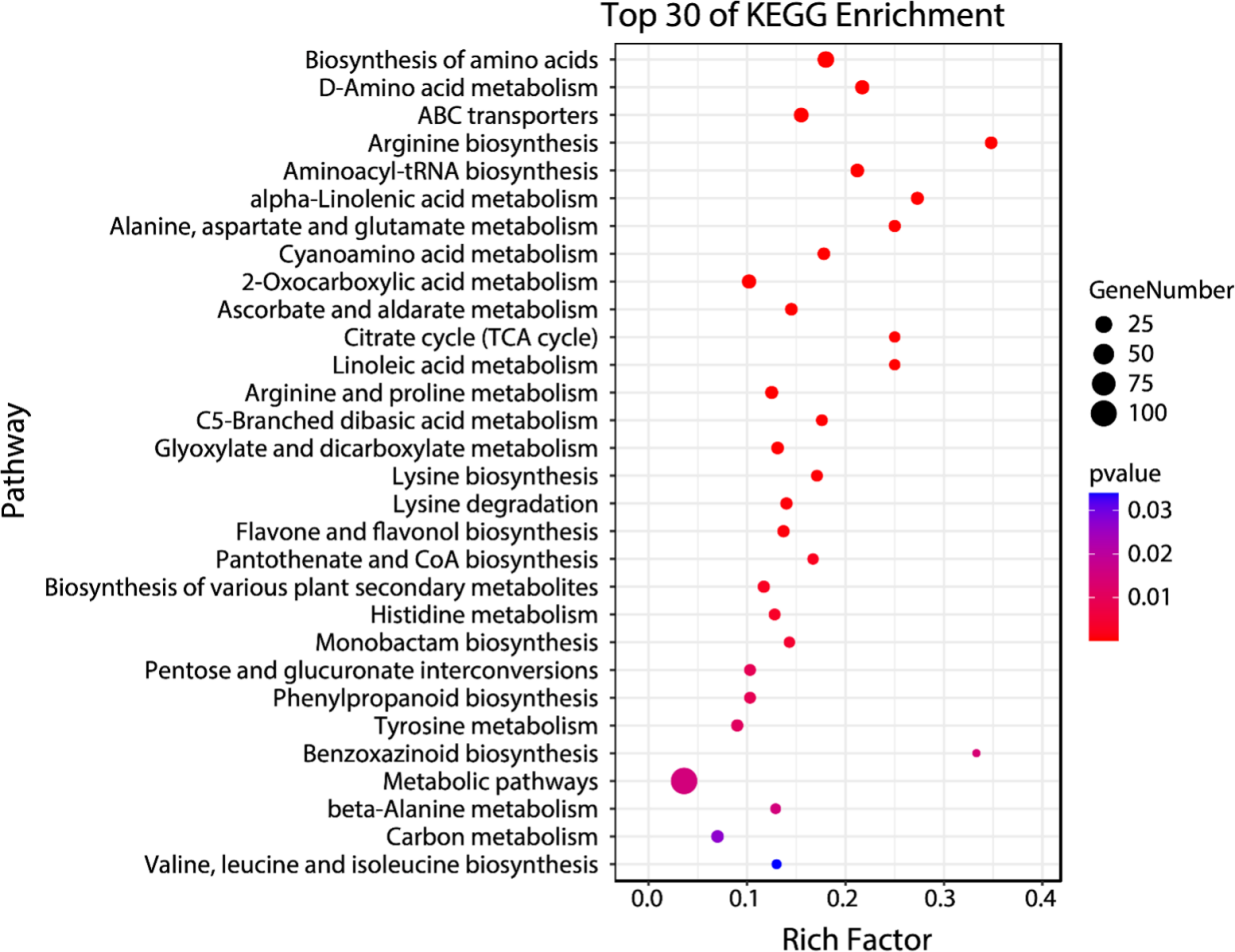
Bubble plot of top 30 enriched KEGG pathways for early biomarker compounds. Rich factor is the ratio of the counts of differential metabolites to the total metabolite number in a certain pathway. The p-value < 0.05 indicated the significance of enrichment of one certain pathway. The color and size of the dots represent the range of the p-value and the number of differential metabolites mapped to the indicated pathways, respectively.

The enrichment analysis which was conducted on temporal differential metabolites has provided important information about the regulations of specific pathways triggered by *C*Las infection. These findings might be able to explain the distinct tolerance levels observed in the HLB-tolerant (‘LB8-9’ Sugar Belle^®^) and HLB-sensitive (‘Valencia’) cultivars when affected by HLB. Among the enriched pathways identified (**Figure 4**), several have been extensively investigated by researchers due to their recognized roles in defense against citrus HLB, including alpha-linolenic acid metabolism, linoleic acid metabolism, flavone and flavonol biosynthesis, and phenylpropanoid biosynthesis (Rao et al., 2018; Suh et al., 2018; Qiu et al., 2020; Cui et al., 2022; Sun et al., 2022; Xue et al., 2022). Hence, it was significant to investigate the regulation of these pathways during the initial phases of *C*Las infection, as it could unveil the underlying factors contributing to the exceptional tolerance exhibited by the ‘Sugar Belle^®^’ cultivar. By investigating these intricate biological mechanisms, we acquire insights into the distinctive characteristics that contributed to the tolerance of ‘Sugar Belle^®^’ and obtain valuable functional genetic information for breeding HLB-tolerant cultivars.

### 4.1 Regulations of key defense pathways against *C*Las infection within 48 hpi

**Figure 5:**
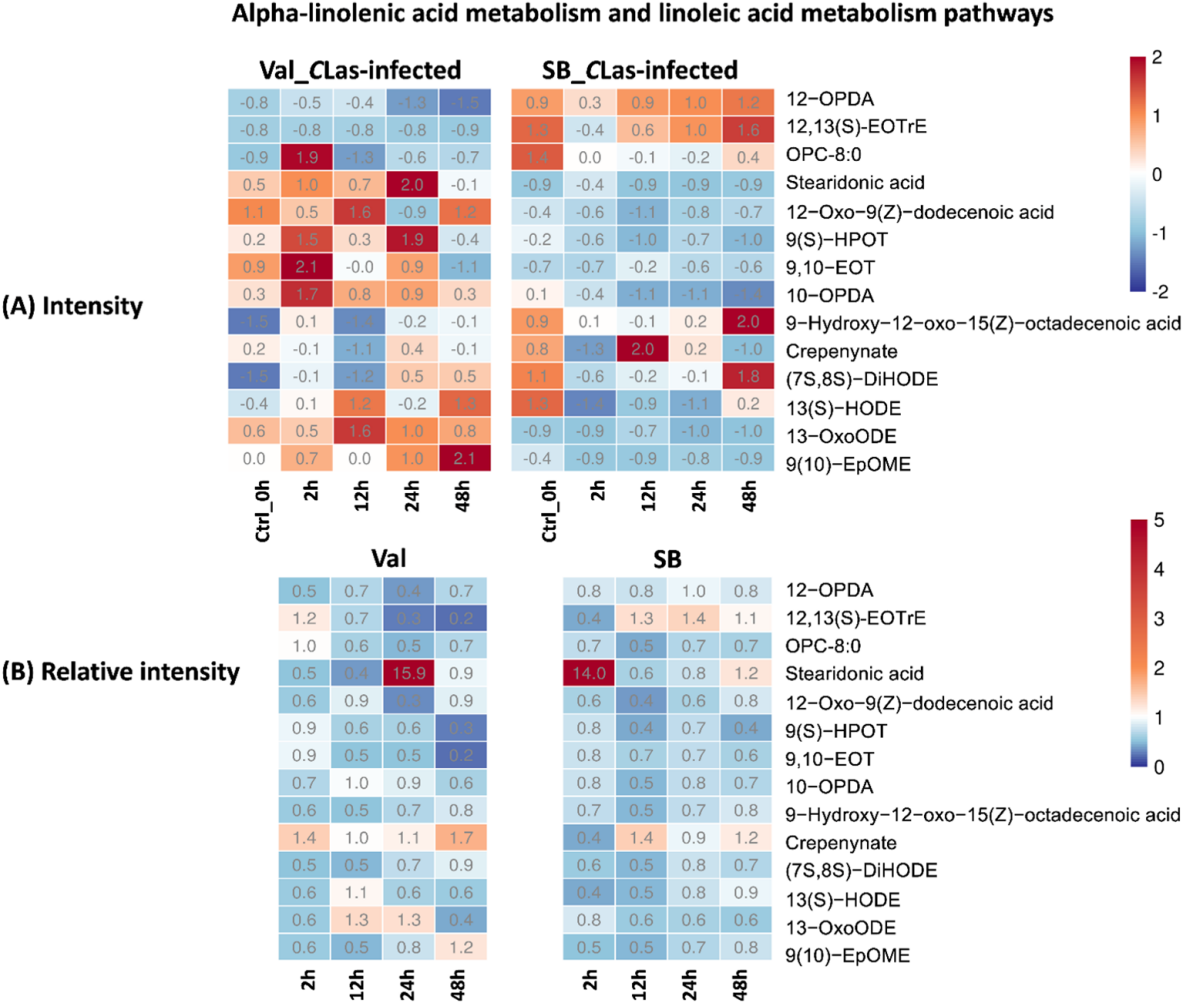
Intensity and relative intensity of metabolites in alpha-linolenic acid metabolism and linoleic acid metabolism pathways. **(A)** Heatmaps for the metabolite intensities in control (0 hpi) and CLas-infected samples at 2 hpi, 12 hpi, 24 hpi, 48 hpi. **(B)** Heatmaps for the relative intensity at 2 hpi, 12 hpi, 24 hpi, 48 hpi. Relative intensity is defined as the ratio of metabolite intensities in CLas-infected samples compared to CLas-free samples.

The alpha-linolenic acid metabolism and linoleic acid metabolism pathways are both categorized under lipid metabolism. In these pathways, alpha-linolenic acid (an omega-3 fatty acid) and linoleic acid (an essential omega-6 fatty acid) undergo a series of enzymatic reactions, such as desaturation and elongation, leading to the production of various metabolites. Oxylipins, the product compound of these two pathways, can act as anti-bacterial molecules defensing against HLB by inhibiting the growth of *C*Las (Jain et al., 2019). They are involved in activating plant immune responses, regulating gene expression, and coordinating defense-related processes (Prost et al., 2005; Suh et al., 2018). In this study, the metabolites on these two pathways include 12-OPDA, (9Z,15Z)-(13S)-12,13-epoxyoctadeca-9,11,15-trienoic acid, 8-[(1R,2R)-3-Oxo-2-{(Z)-pent-2-enyl}cyclopentyl]octanoate (OPC-8:0), stearidonic acid, 12-oxo-9(Z)-dodecenoic acid, 9(S)-HPOT, 9,10-EOT, 10-OPDA, 9-hydroxy-12-oxo-15(Z)-octadecenoic acid, crepenynate, (7S,8S)-DiHODE,13(S)-HODE, 13-OxoODE, 9(10)-EpOME; (9R,10S)-(12Z)-9,10-epoxyoctadecenoic acid, were detected and tentatively identified. During the 48 hpi, significant variations in fatty acid composition were observed between HLB-sensitive and HLB-tolerant citrus cultivars, as depicted in **Figure 5A**. Specifically, the HLB-tolerant cultivar consistently exhibited higher levels of 12-OPDA and (9Z,15Z)-(13S)-12,13-epoxyoctadeca-9,11,15-trienoic acid in response to *C*Las infection. In contrast, the HLB-sensitive cultivar, ‘Valencia’, showed relatively higher levels of other fatty acids, such as stearidonic acid, 12-oxo-9(Z)-dodecenoic acid, 9(S)-HPOT, 9,10-EOT, and 10-OPDA during the same period. However, when considering the influence of psyllid feeding activities on leaf metabolism, it was more appropriate to compare the relative intensities of these compounds (i.e., intensity ratios of metabolites in *C*Las-infected samples compared to *C*Las-free samples). The relative intensity can serve as an indicator of whether these compounds’ activities were stimulated by *C*Las infection. As shown in **Figure 5B**, the relative intensities of these compounds were similar during the 48-hour period for both cultivars. Moreover, most of the fatty acids detected in the lipid metabolism pathways exhibited a lower relative intensity (represented by the blue color in **Figure 5B**, with a ratio < 1), except for stearidonic acid. These results indicated that *C*Las infection might lead to the down-regulation or increased utilization of metabolites in the lipid metabolism pathways in both HLB-tolerant and HLB-sensitive cultivars. Interestingly, stearidonic acid showed a substantial increase, approximately 14 times higher than in *C*Las-free samples, at 24 hpi of Val_*C*La*s*-infected samples and 2 hpi of SB_*C*Las-infected samples. This finding highlights the unique response of stearidonic acid to *C*Las infection in both cultivars.

**Figure 6:**
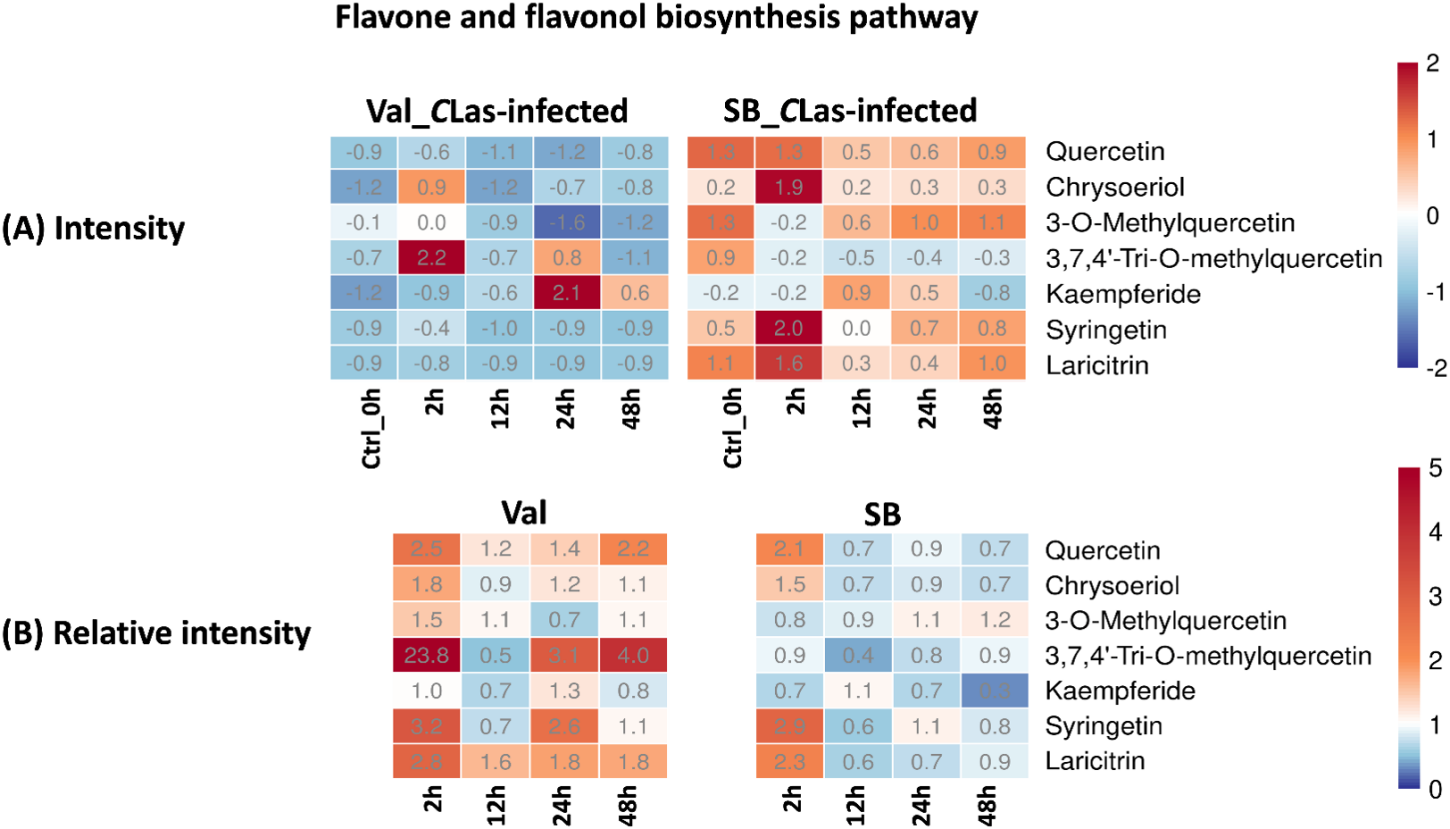
Intensity and relative intensity of metabolites in flavone and flavonol biosynthesis pathways. **(A)** Heatmaps for the metabolite intensities in control (0 hpi) and CLas-infected samples at 2 hpi, 12 hpi, 24 hpi, 48 hpi. **(B)** Heatmaps for the relative intensity at 2 hpi, 12 hpi, 24 hpi, 48 hpi. Relative intensity is defined as the ratio of metabolite intensities in CLas-infected samples compared to CLas-free samples.

Flavones and flavonols are classes of flavonoids, which are secondary metabolites found in plants. These compounds possess antioxidant properties and significantly contribute to the plant’s defense mechanisms against biotic (e.g., herbivory and nematodes) and abiotic stresses (e.g., drought, cold, and UV-radiation) (Chagas et al., 2022). Recent research by Hijaz et al. demonstrated a positive correlation between elevated phenolic and flavonoid leaf contents and enhanced tolerance of citrus plants to the *C*Las bacterium (Hijaz et al., 2020). In this study, the flavone and flavonol biosynthesis pathway was enriched, and several related metabolites were identified, namely quercetin, chrysoeriol, 3-O-methylquercetin, 3,7,4’-tri-O-methylquercetin, kaempferide, syringetin, and laricitrin. As shown in **Figure 6B**, the majority of these compounds displayed relative intensities higher than one after *C*Las infection in the Val_*C*Las-infected samples, indicating an up-regulation of this pathway as a result of *C*Las infection. Nevertheless, quercetin, chrysoeriol, syringetin, and laricitrin were only up-regulated at 2 hpi in SB_*C*Las-infected samples. This implied that the flavonoid-related pathways which can explain HLB tolerance were up-regulated more prominently in the HLB-sensitive cultivar. Nonetheless, this is not the case. As shown in **Figure 6A**, the concentrations of flavonoid compounds remained higher in SB_*C*Las-infected samples compared to Val_*C*Las-infected samples. Despite the up-regulation of the flavonols and flavones synthesis pathway in Val_*C*Las-infected samples from 0 hpi to 48 hpi, the levels of these compounds in Val_*C*Las-infected samples were still lower than that in SB_*C*Las-infected samples. This result aligned with a prior investigation by Hijaz et al., where it was documented that tolerant citrus species exhibited elevated levels of phenolics and flavonoids (Hijaz et al., 2020). It can be hypothesized that HLB-tolerant cultivar could eliminate or decrease *C*Las at the initiate stage when it was introduced either by psyllids or other means due to their high flavonoid content. And this high abundance of flavonoids could enable them to effectively counteract *C*Las that remained in the plant, thereby reducing the *C*Las-triggered stress accumulation. Conversely, HLB-sensitive cultivar with low flavonoid content was unable to eliminate or reduce *C*Las upon initial introduction or subsequent infection. As a result, *C*Las-triggered stresses accumulated within these plants, leading to their susceptibility to HLB.

**Figure 7:**
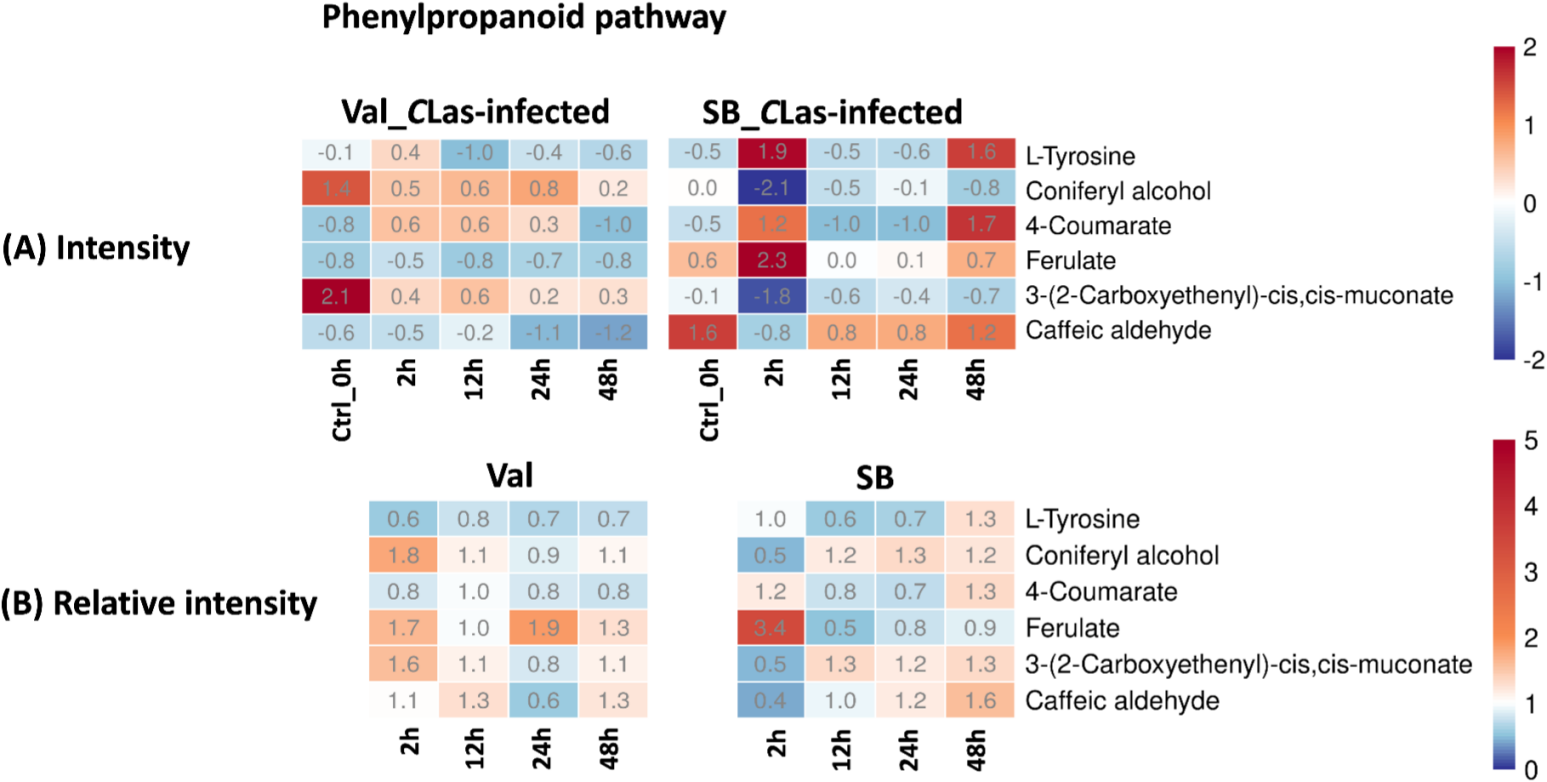
Intensity and relative intensity of metabolites in phenylpropanoid biosynthesis pathways. **(A)** Heatmaps for the metabolite intensities in control (0 hpi) and *C*Las-infected samples at 2 hpi, 12 hpi, 24 hpi, 48 hpi. **(B)** Heatmaps for the relative intensity at 2 hpi, 12 hpi, 24 hpi, 48 hpi. Relative intensity is defined as the ratio of metabolite intensities in *C*Las-infected samples compared to *C*Las-free samples.

Phenylpropanoid pathway plays a crucial role in the synthesis of diverse phenolic compounds in plants, which serve various functions such as providing structural support, defending against pathogens, and offering UV protection (Nicholson and Hammerschmidt, 1992; Hijaz et al., 2020). Within this pathway, several compounds, including L-tyrosine, coniferyl alcohol, 4-coumarate, ferulate, 3-(2-carboxyethenyl)-cis,cis-muconate, and caffeic aldehyde, were identified. **Figure 7B** demonstrated a slight up-regulation of these compounds in response to *C*Las infection in both cultivars. The noticeable fluctuations of concentration (**Figure 7A**), particularly in the SB_*C*Las-infected samples, indicated the active responses of plants to *C*Las infection facilitated by feeding activities of psyllids.

### 4.2 Initial defense mechanisms in HLB-tolerant vs. HLB-sensitive cultivars

In terms of overall metabolites, HLB-tolerant cultivar showed a noticeable response at 2 hpi due to *C*Las infection, which was faster compared to the HLB-sensitive cultivar that showed a significant response at 24 hpi (**Figure 3**). Looking at the details of metabolites, four pathways related to plant defense were enriched based on temporal differential metabolites, and these compounds exhibited various changes. By analyzing the relative intensity of pathway-related compounds in *C*Las-infected samples, it was observed that *C*Las infection led to a reduction in lipid metabolism-related compounds in both HLB-tolerant and HLB-sensitive cultivar (**Figure 5B**). This reduction could be attributed to the plant’s consumption during the defense against *C*Las infection or down-regulation of related pathways due to *C*Las infection. Moreover, regarding flavonoid-related metabolites, HLB-tolerant cultivar showed an increase in some metabolites at 2 hpi, while HLB-sensitive cultivar remained consistently up-regulated from 2 hpi to 48 hpi (**Figure 6B**). Given the markedly reduced concentrations of flavonoids observed in the HLB-sensitive cultivar in comparison to the HLB-tolerant cultivar, (**Figure 6A**), it can be inferred that HLB-sensitive cultivar continuously attempted to replenish their flavonoid levels after *C*Las infection. The higher concentrations of flavonoids observed in HLB-tolerant cultivars could potentially contribute to their tolerance against HLB. In the phenylpropanoid pathway, both cultivars exhibited some level of up-regulation, indicating the defensive effects of this pathway (**Figure 7B**). Overall, there were notable variations in metabolic responses between HLB-tolerant and HLB-sensitive cultivars to *C*Las infection, along the HLB defense-related pathway. It was conclusive that both HLB-tolerant and HLB-sensitive cultivars can react to *C*Las infection as early as 2 hpi and respond with metabolic defenses, particularly by increasing the production of flavonoids.

### 4.3 Insights of defense mechanisms from initial responses to long-term stress

**Figure 8:**
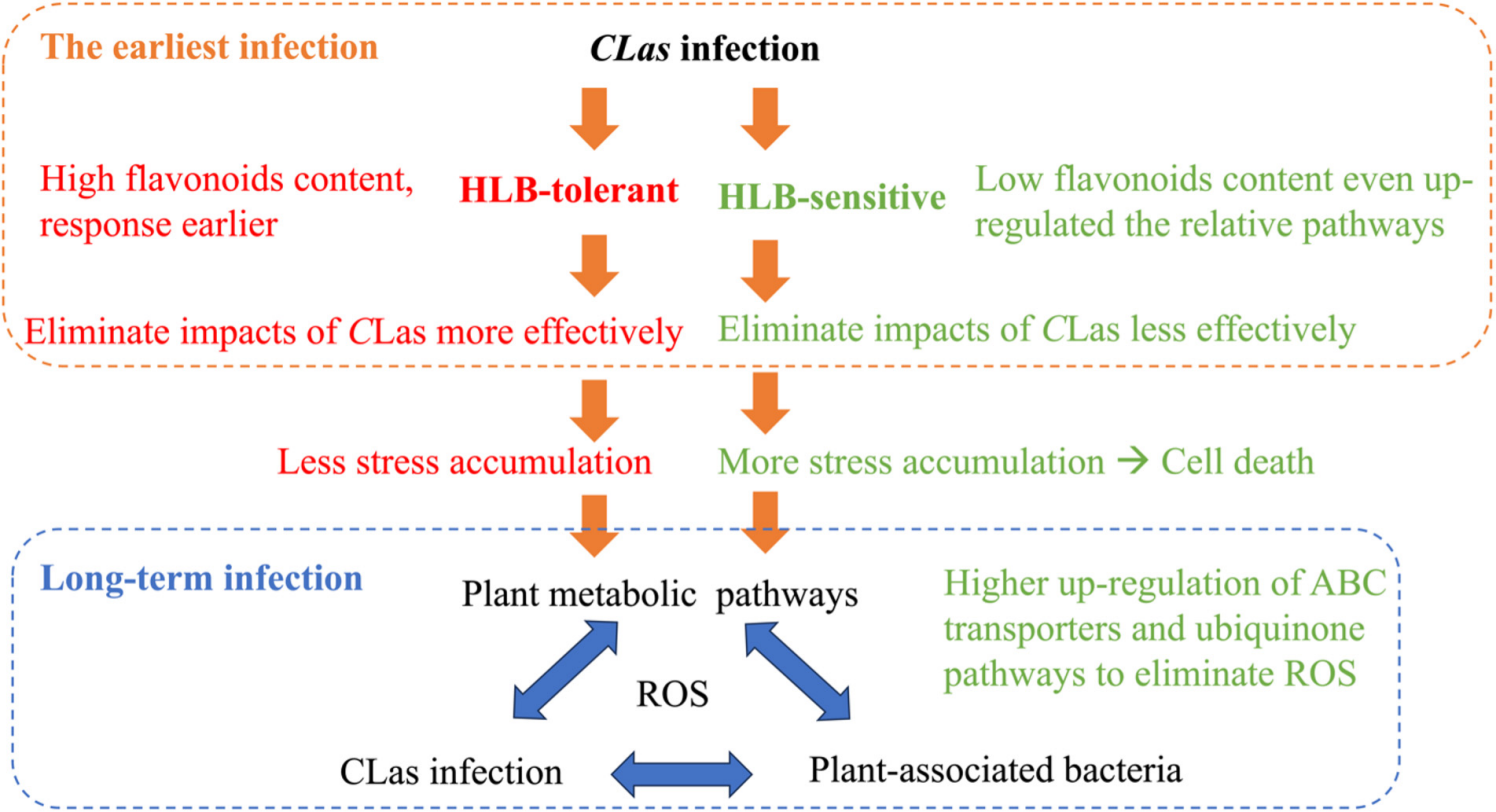
Certain speculations based on the findings of two HLB studies.

Drawing from the findings of this study and our previous study (Li et al., 2023), it is possible to put forward certain speculations as shown in **Figure 8**. When a healthy citrus tree is first infected by *C*Las, HLB-tolerant and HLB-sensitive cultivars show different responses. HLB-tolerant cultivars can exhibit high flavonoid content and a rapid response, eliminating *C*Las-triggered stress (e.g., reactive oxygen species) more effectively. On the other hand, HLB-sensitive cultivars exhibit lower flavonoid content despite continuously up-regulating the flavonoid-relative pathway, resulting in less effective elimination of *C*Las-triggered stress. These initial differences in defense mechanisms lead to HLB-tolerant cultivars accumulating less stress over time, while HLB-sensitive cultivars accumulate more stress, eventually leading to cell death. Furthermore, various other plant pathways come into play in the prolonged battle against HLB, affecting plant-associated bacteria and creating a complex interplay with the disease. To survive, HLB-sensitive cultivars, with their higher reactive oxygen species (ROS) levels, regulate pathways that can eliminate ROS more efficiently compared to HLB-tolerant cultivars. This intricate web of responses sheds light on the temporal struggle between the citrus plants and *C*Las infection.

### 4.4 Implications for disease management

The insights from this study underscore the potential for improving disease resistance from the earliest stages of infection. Understanding the role of key metabolites and pathways, including alpha-linolenic acid metabolism, linoleic acid metabolism, flavone and flavonol biosynthesis, and phenylpropanoid biosynthesis, could advance disease management strategies. For instance, by selecting for cultivars with higher flavonoid content, which is linked to the up-regulation of flavone and flavonol biosynthesis pathways, breeders can create citrus plants that are better equipped to manage initial infection stresses. This proactive approach can help in mitigating the spread of HLB before it establishes a foothold, ultimately reducing the need for reactive treatments and improving overall plant health. Moreover, for trees that are already infected but still alive, focusing on pathways involved in stress response and antioxidant defense could lead to novel therapeutic strategies. For example, developing microbial treatments that enhance the ABC transporters pathway, which is slightly up-regulated in response to HLB affection, could improve the plant’s ability to manage oxidative stress and delay disease progression. Such treatments might not only help in extending the productive life of currently infected trees but also in reducing the severity of the disease, providing a more sustainable approach to managing existing infections. Overall, this study’s findings pave the way for both immediate and long-term solutions to control citrus HLB. By integrating these insights into breeding programs, we could enhance cultivar performance and resilience. Developing cultivars that effectively decrease *C*Las infection and manage oxidative stress by utilizing critical metabolic pathways could lead to significant reductions in economic losses due to HLB. This approach could help ensure greater productivity in citrus agriculture, benefiting growers through improved yield and reduced costs associated with disease management.

To summarize, this study explored the specific pathway regulations triggered by CLas infection in citrus leaves. By using untargeted metabolomics and machine learning methods, the temporal differential metabolites in citrus fresh leaves within 48 hours post-infection were analyzed. The results revealed that flavone and flavonol biosynthesis pathways, which are related to the production of secondary metabolites with antioxidant properties, were significantly up-regulated in response to *C*Las infection. High flavonoids content in the HLB-tolerant cultivar might enhance the tolerance of citrus plants to *C*Las by potentially counteracting the initial infection and reducing *C*Las-triggered stress accumulation. Furthermore, the phenylpropanoid pathway, involved in the synthesis of phenolic compounds, showed slight up-regulation in response to *C*Las infection in both cultivars. These vital pathways could serve as valuable biomarkers in future breeding programs that aimed at developing HLB-tolerant citrus cultivars. Based on this study and our previous one, we can conclude that when citrus trees are first infected by *C*Las, HLB-tolerant and HLB-sensitive cultivars respond differently. HLB-tolerant cultivars have high flavonoid content and rapidly eliminate stress like ROS, while HLB-sensitive cultivars, despite up-regulating flavonoid pathways, have lower flavonoid content and struggle to eliminate stress effectively. Over time, HLB-tolerant cultivars accumulate less stress, while HLB-sensitive ones accumulate more, leading to cell death. The prolonged battle against HLB involves other plant pathways and bacteria interactions. HLB-sensitive cultivars with higher ROS levels regulate pathways to eliminate ROS more efficiently than HLB-tolerant ones, illustrating the complex struggle between citrus plants and *C*Las infection. These findings offer valuable insights for developing more tolerant cultivars, potentially serving as a sustainable, long-term solution for controlling citrus HLB.

## Data Availability

The datasets presented in this study can be found in online repositories. The names of the repository/repositories and accession number(s) can be found in the article/**Supplementary Material**. The Python scripts used for multiple machine learning models and external testing are available on GitHub at https://github.com/Yu-Wang-Lab/Multiple_ML_modeling_for_HLB_prediction.
