# Supplementary material for: Identifying the earliest citrus responses to Candidatus Liberibacter asiaticus infection: a temporal metabolomics study
Source: Front Plant Sci. 2024 Nov 6;15:1455344. doi: 10.3389/fpls.2024.1455344 (PMC11579704; doi:10.3389/fpls.2024.1455344)
Supplement: Supplementary file 1 [file DataSheet1.docx]

**Identifying the Earliest Citrus Responses to *Candidatus* Liberibacter asiaticus** (***C*Las) Infection: A Temporal Metabolomics Study**

**Jingwen Li, Yuanzhi Zimmy Wang, Fred G. Gmitter Jr., Yu Wang***

Citrus Research and Education Center, Institute of Food and Agricultural Sciences, University of Florida, 700 Experiment Station Rd, Lake Alfred, FL 33850

^*^Corresponding author: Dr. Yu Wang, Citrus Research & Education Center, Institute of Food and Agricultural Sciences, University of Florida, 700 Experiment Station Road, Lake Alfred, Florida 33850, United States; E-mail: [yu.wang@ufl.edu](file:///E:\UF\My%20Projects\Star%20fruit\Manuscript\yu.wang@ufl.edu)

**Figure S1.** The numbers of temporal differential metabolites in response to *C*Las infection from 0 to 48 hpi.

**Table S1.** Parameters utilized in the application of six machine learning algorithms for the analysis of untargeted metabolomics data.

**Table S2.** Average accuracies obtained through a 6-fold cross-validation for individual machine learning model applied to untargeted metabolomics data.

**Table S3.** Annotation and MS information of temporal differential metabolites identified in citrus leaves.

**Table S4.** Metabolic pathways identified through enrichment analysis of temporal differential metabolites.

**Figure S1.**

**Table S1.**

| Sub-package | Functions | Parameters |
| --- | --- | --- |
| ensemble | RandomForestClassifier | n_estimators=100, criterion=”gini”, max_depth=None, min_samples_split=2, min_samples_leaf=1, min_weight_fraction_leaf=0.0, max_features=”sqrt”, max_leaf_nodes=None, min_impurity_decrease=0.0, bootstrap=True, oob_score=False, n_jobs=None, random_state=None, verbose=0, warm_start=False, class_weight=None, ccp_alpha=0.0, max_samples=None, |
| ensemble | GradientBoostingClassifier | loss=’log_loss’, learning_rate=0.1, n_estimators=100, subsample=1.0, criterion=’friedman_mse’, min_samples_split=2, min_samples_leaf=1, min_weight_fraction_leaf=0.0, max_depth=3, min_impurity_decrease=0.0, init=None, random_state=0, max_features=None, verbose=0, max_leaf_nodes=None, warm_start=False, validation_fraction=0.1, n_iter_no_change=None, tol=1e-4, ccp_alpha=0.0, |
| linear_model | LogisticRegression | penalty='l1', dual=False, tol =1e-4, C=1.0, fit_intercept=True, intercept_scaling=1, class_weight=None, random_state=None, solver='liblinear', max_iter=100, multi_class='auto', verbose=0, warm_start=False, n_jobs=None, l1_ratio=None |
| linear_model | LogisticRegression | penalty = 'l2', dual=False, tol =1e-4, C=1.0, fit_intercept=True, intercept_scaling=1, class_weight=None, random_state=None, solver = 'liblinear', max_iter=100, multi_class='auto', verbose=0, warm_start=False, n_jobs=None, l1_ratio=None |
| svm | SVC | C=1.0, kernel='rbf', degree=3, gamma=’scale’, coef0=0.0, shrinking=True, probability=False, tol=1e-3, cache_size=200, class_weight=None, verbose=False, max_iter=-1, decision_function_shape=’ovr’, break_ties=False, random_statei=None, |
| neural_network | MLPClassifier | hidden_layer_sizes=(10,4), activation=’relu’, solver=’adam’, alpha=0.00001, batch_size=’auto’, learning_rate=’constant’, learning_rate_init=0.001, power_t=0.5, max_iter=200, shuffle=True, random_stateint, RandomState=1, tol=1e-4, verbose=False, warm_start=False, momentum=0.9, nesterovs_momentum=True, early_stopping=False, validation_fraction=0.1, beta_1=0.9, beta_2=0.999, epsilon=1e-8, n_iter_no_change=10, max_fun=15000 |

**Table S2.**

| Method | Accuracy | Sensitivity | Specificity | Precision | freeative Predictive Value | F1 Score | AUC | MCC |
| --- | --- | --- | --- | --- | --- | --- | --- | --- |
| SB_Ctrl_vs_SB_*C*Las-infected_2h | | | | | | | | |
| LR-L1 | 3.33 ± 7.45 | 0.00 ± 0.00 | 5.56 ± 12.42 | 0.00 ± 0.00 | 5.56 ± 12.42 | nan ± nan | 0.00 ± 0.00 | -94.44 ± 12.42 |
| LR-L2 | 0.00 ± 0.00 | 0.00 ± 0.00 | 0.00 ± 0.00 | 0.00 ± 0.00 | 0.00 ± 0.00 | nan ± nan | 0.00 ± 0.00 | -100.00 ± 0.00 |
| MLP | 53.33 ± 14.91 | 75.00 ± 26.79 | 30.56 ± 22.40 | 54.17 ± 14.59 | nan ± nan | 60.75 ± 17.01 | 45.83 ± 23.45 | 7.43 ± 26.62 |
| RF | 53.33 ± 9.43 | 100.00 ± 0.00 | 0.00 ± 0.00 | 53.33 ± 9.43 | nan ± nan | 69.05 ± 8.42 | 19.44 ± 15.71 | 0.00 ± 0.00 |
| GB | 53.33 ± 24.94 | 69.44 ± 24.37 | 36.11 ± 33.91 | 55.56 ± 22.91 | 50.00 ± 40.82 | 60.63 ± 21.62 | 56.94 ± 34.16 | 5.56 ± 55.00 |
| SVM | 93.33 ± 9.43 | 100.00 ± 0.00 | 88.89 ± 15.71 | 88.89 ± 15.71 | 100.00 ± 0.00 | 93.33 ± 9.43 | 100.00 ± 0.00 | 88.89 ± 15.71 |
| SB_*C*Las-infected_2h_vs_SB_*C*Las-infected_12h | | | | | | | | |
| LR-L1 | 0.00 ± 0.00 | 0.00 ± 0.00 | 0.00 ± 0.00 | 0.00 ± 0.00 | 0.00 ± 0.00 | nan ± nan | 0.00 ± 0.00 | -100.00 ± 0.00 |
| LR-L2 | 0.00 ± 0.00 | 0.00 ± 0.00 | 0.00 ± 0.00 | 0.00 ± 0.00 | 0.00 ± 0.00 | nan ± nan | 0.00 ± 0.00 | -100.00 ± 0.00 |
| MLP | 33.33 ± 18.63 | 41.67 ± 44.88 | 33.33 ± 38.49 | nan ± nan | nan ± nan | nan ± nan | 25.00 ± 18.63 | -26.29 ± 43.61 |
| RF | 20.83 ± 17.18 | 50.00 ± 50.00 | 16.67 ± 37.27 | nan ± nan | nan ± nan | nan ± nan | 18.06 ± 19.49 | -33.33 ± 47.14 |
| GB | 50.00 ± 25.00 | 16.67 ± 37.27 | 66.67 ± 38.49 | nan ± nan | 61.11 ± 31.06 | nan ± nan | 38.89 ± 20.79 | -16.67 ± 41.94 |
| SVM | 95.83 ± 9.32 | 100.00 ± 0.00 | 94.44 ± 12.42 | 91.67 ± 18.63 | 100.00 ± 0.00 | 94.44 ± 12.42 | 100.00 ± 0.00 | 92.96 ± 15.75 |
| SB_*C*Las-infected_12h_vs_SB_*C*Las-infected_24h | | | | | | | | |
| LR-L1 | 0.00 ± 0.00 | 0.00 ± 0.00 | 0.00 ± 0.00 | 0.00 ± 0.00 | 0.00 ± 0.00 | nan ± nan | 0.00 ± 0.00 | -100.00 ± 0.00 |
| LR-L2 | 0.00 ± 0.00 | 0.00 ± 0.00 | 0.00 ± 0.00 | 0.00 ± 0.00 | 0.00 ± 0.00 | nan ± nan | 0.00 ± 0.00 | -100.00 ± 0.00 |
| MLP | 11.11 ± 15.71 | 8.33 ± 18.63 | 8.33 ± 18.63 | 8.33 ± 18.63 | 8.33 ± 18.63 | nan ± nan | 16.67 ± 23.57 | -83.33 ± 23.57 |
| RF | 25.00 ± 18.63 | 50.00 ± 40.82 | 0.00 ± 0.00 | 30.56 ± 22.40 | nan ± nan | nan ± nan | 0.00 ± 0.00 | -50.00 ± 40.82 |
| GB | 22.22 ± 15.71 | 33.33 ± 37.27 | 8.33 ± 18.63 | 22.22 ± 22.91 | nan ± nan | nan ± nan | 8.33 ± 18.63 | -58.33 ± 34.36 |
| SVM | 94.44 ± 12.42 | 100.00 ± 0.00 | 91.67 ± 18.63 | 91.67 ± 18.63 | 100.00 ± 0.00 | 94.44 ± 12.42 | 100.00 ± 0.00 | 91.67 ± 18.63 |
| SB_*C*Las-infected_24h_vs_SB_*C*Las-infected_48h | | | | | | | | |
| LR-L1 | 0.00 ± 0.00 | 0.00 ± 0.00 | 0.00 ± 0.00 | 0.00 ± 0.00 | 0.00 ± 0.00 | nan ± nan | 0.00 ± 0.00 | -100.00 ± 0.00 |
| LR-L2 | 0.00 ± 0.00 | 0.00 ± 0.00 | 0.00 ± 0.00 | 0.00 ± 0.00 | 0.00 ± 0.00 | nan ± nan | 0.00 ± 0.00 | -100.00 ± 0.00 |
| MLP | 50.00 ± 30.43 | 58.33 ± 44.88 | 33.33 ± 47.14 | nan ± nan | nan ± nan | nan ± nan | 33.33 ± 47.14 | -8.33 ± 60.67 |
| RF | 50.00 ± 13.61 | 100.00 ± 0.00 | 0.00 ± 0.00 | 50.00 ± 13.61 | nan ± nan | 65.56 ± 12.27 | 0.00 ± 0.00 | 0.00 ± 0.00 |
| GB | 44.44 ± 22.91 | 66.67 ± 37.27 | 25.00 ± 38.19 | 50.00 ± 28.87 | nan ± nan | nan ± nan | 25.00 ± 28.87 | -8.33 ± 53.36 |
| SVM | 100.00 ± 0.00 | 100.00 ± 0.00 | 100.00 ± 0.00 | 100.00 ± 0.00 | 100.00 ± 0.00 | 100.00 ± 0.00 | 100.00 ± 0.00 | 100.00 ± 0.00 |
| Val_Ctrl_vs_Val_*C*Las-infected_2h | | | | | | | | |
| LR-L1 | 10.00 ± 15.28 | 0.00 ± 0.00 | 12.50 ± 19.09 | 0.00 ± 0.00 | 19.44 ± 27.92 | nan ± nan | 0.00 ± 0.00 | -83.68 ± 23.82 |
| LR-L2 | 0.00 ± 0.00 | 0.00 ± 0.00 | 0.00 ± 0.00 | 0.00 ± 0.00 | 0.00 ± 0.00 | nan ± nan | 0.00 ± 0.00 | -100.00 ± 0.00 |
| MLP | 70.00 ± 27.69 | 33.33 ± 47.14 | 84.72 ± 24.73 | nan ± nan | 74.72 ± 23.22 | nan ± nan | 61.11 ± 37.16 | 18.06 ± 62.07 |
| RF | 40.00 ± 11.55 | 25.00 ± 38.19 | 45.83 ± 13.39 | 11.11 ± 15.71 | 63.89 ± 17.79 | nan ± nan | 29.17 ± 17.18 | -26.59 ± 32.79 |
| GB | 86.67 ± 9.43 | 50.00 ± 50.00 | 95.83 ± 9.32 | nan ± nan | 90.00 ± 10.00 | nan ± nan | 72.92 ± 23.29 | 43.54 ± 45.42 |
| SVM | 100.00 ± 0.00 | 100.00 ± 0.00 | 100.00 ± 0.00 | 100.00 ± 0.00 | 100.00 ± 0.00 | 100.00 ± 0.00 | 100.00 ± 0.00 | 100.00 ± 0.00 |
| Val_*C*Las-infected_2h_vs_Val_*C*Las-infected_12h | | | | | | | | |
| LR-L1 | 0.00 ± 0.00 | 0.00 ± 0.00 | 0.00 ± 0.00 | 0.00 ± 0.00 | 0.00 ± 0.00 | nan ± nan | 0.00 ± 0.00 | -100.00 ± 0.00 |
| LR-L2 | 5.56 ± 12.42 | 8.33 ± 18.63 | 0.00 ± 0.00 | 8.33 ± 18.63 | 0.00 ± 0.00 | nan ± nan | 0.00 ± 0.00 | -91.67 ± 18.63 |
| MLP | 61.11 ± 22.91 | 100.00 ± 0.00 | 16.67 ± 37.27 | 61.11 ± 22.91 | nan ± nan | 73.33 ± 17.95 | 50.00 ± 43.30 | 16.67 ± 37.27 |
| RF | 50.00 ± 16.67 | 91.67 ± 18.63 | 0.00 ± 0.00 | 52.78 ± 14.96 | nan ± nan | 65.00 ± 15.00 | 37.50 ± 34.61 | -8.33 ± 18.63 |
| GB | 38.89 ± 22.91 | 66.67 ± 37.27 | 0.00 ± 0.00 | 44.44 ± 22.91 | nan ± nan | nan ± nan | 37.50 ± 34.61 | -33.33 ± 37.27 |
| SVM | 94.44 ± 12.42 | 100.00 ± 0.00 | 83.33 ± 37.27 | 94.44 ± 12.42 | nan ± nan | 96.67 ± 7.45 | 100.00 ± 0.00 | 83.33 ± 37.27 |
| Val_*C*Las-infected_12h_vs_Val_*C*Las-infected_24h | | | | | | | | |
| LR-L1 | 5.56 ± 12.42 | 0.00 ± 0.00 | 8.33 ± 18.63 | 0.00 ± 0.00 | 8.33 ± 18.63 | nan ± nan | 0.00 ± 0.00 | -91.67 ± 18.63 |
| LR-L2 | 0.00 ± 0.00 | 0.00 ± 0.00 | 0.00 ± 0.00 | 0.00 ± 0.00 | 0.00 ± 0.00 | nan ± nan | 0.00 ± 0.00 | -100.00 ± 0.00 |
| MLP | 61.11 ± 29.92 | 66.67 ± 47.14 | 66.67 ± 37.27 | nan ± nan | nan ± nan | nan ± nan | 58.33 ± 44.88 | 33.33 ± 55.28 |
| RF | 55.56 ± 15.71 | 83.33 ± 37.27 | 25.00 ± 38.19 | nan ± nan | nan ± nan | nan ± nan | 54.17 ± 41.87 | 8.33 ± 18.63 |
| GB | 44.44 ± 31.43 | 25.00 ± 38.19 | 66.67 ± 37.27 | nan ± nan | 47.22 ± 29.53 | nan ± nan | 45.83 ± 33.59 | -8.33 ± 67.19 |
| SVM | 77.78 ± 24.85 | 100.00 ± 0.00 | 58.33 ± 44.88 | 75.00 ± 26.79 | nan ± nan | 82.78 ± 19.29 | 100.00 ± 0.00 | 58.33 ± 44.88 |
| Val_*C*Las-infected_24h_vs_Val_*C*Las-infected_48h | | | | | | | | |
| LR-L1 | 0.00 ± 0.00 | 0.00 ± 0.00 | 0.00 ± 0.00 | 0.00 ± 0.00 | 0.00 ± 0.00 | nan ± nan | 0.00 ± 0.00 | -100.00 ± 0.00 |
| LR-L2 | 0.00 ± 0.00 | 0.00 ± 0.00 | 0.00 ± 0.00 | 0.00 ± 0.00 | 0.00 ± 0.00 | nan ± nan | 0.00 ± 0.00 | -100.00 ± 0.00 |
| MLP | 36.11 ± 27.92 | 33.33 ± 47.14 | 33.33 ± 47.14 | nan ± nan | nan ± nan | nan ± nan | 16.67 ± 23.57 | -33.33 ± 47.14 |
| RF | 38.89 ± 28.33 | 66.67 ± 47.14 | 0.00 ± 0.00 | 38.89 ± 28.33 | nan ± nan | nan ± nan | 0.00 ± 0.00 | -33.33 ± 47.14 |
| GB | 36.11 ± 20.22 | 25.00 ± 38.19 | 58.33 ± 44.88 | nan ± nan | nan ± nan | nan ± nan | 37.50 ± 40.18 | -16.67 ± 47.14 |
| SVM | 75.00 ± 26.79 | 100.00 ± 0.00 | 58.33 ± 44.88 | 72.22 ± 28.33 | nan ± nan | 80.56 ± 20.22 | 100.00 ± 0.00 | 58.33 ± 44.88 |

**Table S3.**

| compound anme | [M+H]+ m/z | characteristic fragment ion m/z(relative abundance %) |
| --- | --- | --- |
| (-)-Carvone | 151.11156 | 53 (6), 53.04 (6), 55.02 (5), 55.05 (20), 57.03 (4), 57.07 (3), 59.05 (2), 65.04 (3), 67.05 (28), 69.03 (100), 69.07 (3), 71.05 (14), 79.05 (16), 81.03 (3), 81.07 (87), 83.05 (1), 91.05 (22), 93.07 (35), 94.04 (1), 95.05 (31), 95.09 (11), 96.06 (7), 97.06 (8), 105.07 (27), 107.05 (2), 107.09 (30), 109.06 (58), 109.1 (16), 110.07 (1), 123.08 (19), 123.12 (19), 131.09 (1), 133.1 (15), 151.11 (39) |
| (-)-Medicocarpin | 433.14856 | 127.04 (1), 357.1 (2), 375.11 (2), 399.11 (1), 417.12 (4), 418.13 (8), 433.15 (100) |
| (+)-Camphene | 137.13229 | 53.04 (1), 55.05 (5), 57.07 (9), 67.05 (19), 69.07 (5), 79.05 (6), 81.07 (63), 93.07 (5), 95.09 (100), 109.1 (6), 137.13 (13) |
| (1'S)-Averantin | 373.12744 | 283.06 (1), 295.06 (1), 297.08 (2), 299.06 (1), 313.07 (5), 315.09 (8), 327.05 (3), 327.09 (10), 329.1 (11), 339.09 (5), 341.07 (2), 341.1 (2), 343.08 (32), 357.1 (12), 373.13 (100) |
| (15S)-15-Hydroxy-5,8,11-cis-13-trans-eicosatetraenoate | 321.24231 | 55.05 (8), 67.05 (3), 81.07 (7), 91.05 (5), 93.07 (5), 95.09 (8), 109.06 (15), 123.08 (41), 135.08 (5), 151.11 (4), 179.14 (60), 265.18 (7), 303.23 (5), 321.24 (100) |
| (2R,3R)-3-Methylglutamyl-5-semialdehyde-N6-lysine | 274.17593 | 60.04 (4), 69.07 (3), 70.07 (44), 72.08 (24), 84.04 (74), 84.08 (18), 86.1 (100), 115.09 (14), 116.07 (12), 129.1 (9), 130.09 (4), 143.08 (13), 147.11 (4), 161.09 (94), 203.14 (4), 204.13 (6), 257.15 (6), 274.18 (15) |
| (2S,5S)-trans-Carboxymethylproline | 174.07562 | 55.05 (4), 68.05 (4), 70.07 (25), 71.05 (4), 82.07 (21), 84.08 (2), 86.06 (2), 88.04 (1), 100.08 (1), 114.05 (100), 115.04 (1), 128.03 (3), 128.07 (54), 174.08 (3) |
| (6Z,9Z,12Z)-Octadecatrienoic acid | 277.21722 | 59.01 (1), 277.22 (100) |
| (7S,8S)-DiHODE | 311.22293 | 57.03 (2), 139.11 (18), 171.1 (85), 183.1 (6), 201.11 (13), 293.21 (26), 311.22 (100) |
| (9Z,11E)-(13S)-13-Hydroperoxyoctadeca-9,11-dienoic acid | 313.23697 | 55.02 (9), 55.05 (21), 57.03 (8), 57.07 (11), 67.05 (44), 69.07 (11), 71.05 (10), 71.09 (61), 79.05 (21), 81.07 (36), 83.05 (15), 83.09 (19), 85.06 (6), 91.05 (11), 93.07 (29), 95.05 (4), 95.09 (25), 97.06 (9), 97.1 (10), 99.08 (53), 105.07 (13), 107.09 (18), 109.1 (18), 121.1 (13), 123.12 (5), 125.1 (3), 127.11 (16), 133.1 (11), 135.12 (16), 141.13 (63), 147.12 (4), 149.13 (4), 151.11 (11), 153.13 (8), 161.13 (15), 163.11 (17), 165.13 (7), 169.12 (24), 175.15 (3), 179.14 (7), 259.21 (7), 277.22 (58), 295.23 (100), 313.24 (6) |
| (9Z,12Z,15Z)-Octadecatrienoic acid | 279.23166 | 53 (2), 53.04 (2), 55.02 (2), 55.05 (13), 57.03 (2), 57.07 (14), 65.04 (2), 67.05 (94), 69.07 (12), 71.05 (2), 71.09 (1), 79.05 (17), 81.07 (100), 83.05 (2), 83.09 (10), 85.06 (2), 85.1 (30), 89.06 (1), 91.05 (14), 93.07 (19), 95.09 (85), 97.06 (3), 97.1 (6), 99.08 (1), 105.07 (9), 107.09 (16), 109.07 (1), 109.1 (47), 111.08 (2), 111.12 (1), 113.1 (41), 117.07 (2), 119.09 (4), 121.1 (12), 123.08 (1), 123.12 (22), 125.1 (1), 131.11 (2), 133.1 (18), 135.08 (1), 135.12 (6), 137.1 (1), 137.13 (8), 139.11 (1), 145.1 (3), 147.12 (4), 149.1 (5), 149.13 (17), 151.11 (7), 151.15 (4), 153.13 (2), 159.12 (4), 161.13 (3), 163.11 (1), 163.15 (2), 165.13 (3), 165.16 (1), 167.11 (1), 167.14 (7), 173.13 (3), 175.15 (1), 177.13 (2), 181.12 (1), 181.16 (2), 187.15 (4), 191.14 (1), 195.14 (2), 205.16 (1), 209.15 (3), 219.21 (1), 223.17 (2), 233.23 (1), 243.21 (7), 261.22 (12), 279.23 (73) |
| (9Z,15Z)-(13S)-12,13-Epoxyoctadeca-9,11,15-trienoic acid | 293.2106 | 55.02 (15), 55.05 (38), 67.05 (52), 69.07 (25), 79.05 (41), 81.03 (12), 81.07 (48), 83.09 (14), 91.05 (43), 93.07 (36), 95.05 (25), 95.09 (46), 97.06 (16), 97.1 (15), 105.07 (35), 107.05 (10), 107.09 (57), 109.06 (10), 109.1 (25), 119.09 (20), 125.1 (14), 133.1 (29), 147.12 (10), 149.1 (15), 151.11 (32), 155.11 (14), 159.12 (9), 275.2 (100), 293.21 (89) |
| (Indol-3-yl)glycolaldehyde | 176.07043 | 106.07 (2), 108.08 (1), 116.05 (1), 130.07 (1), 134.06 (18), 176.07 (100) |
| (R)-(Indol-3-yl)lactate | 206.08098 | 87.01 (2), 91.05 (2), 103.05 (13), 118.07 (17), 120.08 (100), 146.06 (13), 188.07 (10), 206.08 (7) |
| (R)-2-Hydroxybutane-1,2,4-tricarboxylate | 205.03526 | 55.02 (15), 57.03 (19), 58.01 (1), 59.01 (5), 67.02 (6), 69 (2), 69.03 (3), 71.01 (77), 71.05 (2), 72.99 (36), 73.03 (5), 75.01 (17), 81.03 (23), 83.01 (38), 84.02 (3), 85.03 (79), 86 (2), 87.01 (8), 97.03 (2), 99.01 (22), 99.04 (8), 101.02 (34), 103.04 (6), 111.01 (100), 113.02 (3), 115 (7), 115.04 (3), 117.02 (4), 125.02 (84), 127 (2), 129.02 (8), 131.04 (4), 143 (1), 143.04 (9), 145.01 (10), 161.05 (8), 187.03 (9), 205.04 (64) |
| (R)-2,3-Dihydroxy-3-methylbutanoate | 133.05046 | 59.01 (1), 71.01 (69), 72.99 (17), 87.01 (1), 115 (100), 133.05 (1) |
| (R)-3,3-Dimethylmalate | 163.05995 | 53.04 (4), 55.02 (4), 57.03 (10), 61.03 (16), 69.03 (35), 71.01 (1), 71.05 (2), 73.03 (6), 81.03 (10), 85.03 (100), 87.04 (2), 91.04 (1), 97.03 (19), 99.04 (4), 101.02 (1), 115.04 (1), 127.04 (6), 145.05 (1), 163.06 (1) |
| (R)-Mevalonate | 147.06639 | 57.03 (40), 59.01 (25), 71.01 (90), 71.05 (15), 73.03 (15), 85.03 (63), 87.01 (66), 87.05 (33), 101.06 (100), 103.04 (14), 129.06 (47), 145.05 (5), 147.07 (48) |
| (S)-2-Aceto-2-hydroxybutanoate | 145.05067 | 57.03 (100), 59.01 (24), 71.01 (47), 71.05 (36), 73.03 (9), 81.03 (12), 83.01 (3), 83.05 (54), 85.03 (20), 99.05 (40), 101.02 (5), 101.06 (56), 127.04 (66), 145.05 (93) |
| (S)-Limonene | 137.13214 | 53.04 (6), 55.05 (7), 57.07 (6), 65.04 (5), 67.05 (37), 69.07 (9), 79.05 (17), 81.07 (67), 91.05 (37), 93.07 (11), 95.09 (100), 109.1 (15), 119.09 (6), 137.13 (14) |
| (S)-Malate | 133.01419 | 59.01 (1), 71.01 (74), 72.99 (16), 87.01 (1), 89.02 (8), 115 (100), 133.01 (25) |
| (Z)-But-2-ene-1,2,3-tricarboxylate | 189.03935 | 53 (1), 53.04 (4), 55.02 (4), 55.05 (45), 57.03 (17), 61.03 (4), 69 (10), 69.03 (25), 71.05 (2), 73.03 (13), 73.06 (54), 81.03 (9), 83.01 (2), 83.05 (1), 85.03 (100), 87.01 (19), 87.04 (1), 97.03 (16), 99.04 (5), 101.02 (1), 102.03 (2), 103.04 (4), 109.03 (6), 111.01 (2), 115.04 (3), 117.02 (2), 118.03 (2), 119.04 (4), 127.04 (7), 171.03 (4) |
| [6]-Gingerol | 295.19052 | 55.05 (54), 57.07 (35), 67.05 (74), 69.07 (44), 79.05 (65), 81.03 (51), 81.07 (98), 83.05 (33), 83.09 (35), 85.1 (70), 91.05 (49), 93.07 (47), 95.09 (69), 97.1 (25), 105.07 (35), 107.09 (42), 109.1 (29), 113.1 (100) |
| 1-Methoxycyclohexene | 113.09592 | 55.05 (3), 57.03 (4), 67.05 (28), 71.05 (4), 85.06 (1), 93.07 (3), 95.05 (7), 95.09 (100), 113.1 (36) |
| 1-Methyl-4-(1-methylethenyl)-benzene | 133.10095 | 79.05 (3), 91.05 (2), 93.07 (9), 103.05 (2), 105.07 (100), 133.1 (20) |
| 1-Methyladenosine | 282.11938 | 150.08 (100), 282.12 (6) |
| 1-Methylcyclohexa-1,3-diene | 109.10114 | 53.04 (4), 55.05 (9), 65.04 (3), 67.05 (100), 69.07 (6), 79.05 (6), 80.06 (1), 81.07 (33), 94.08 (1), 107.09 (4), 109.1 (65) |
| 1,3-Cycloheptadiene | 95.08527 | 53.04 (4), 55.05 (30), 65.04 (3), 67.05 (93), 79.05 (1), 80.06 (1), 91.05 (3), 93.07 (16), 95.09 (100) |
| 1,3-Cyclohexadiene | 81.06975 | 53.04 (15), 55.05 (1), 65.04 (3), 66.05 (3), 79.05 (75), 81.07 (100) |
| 1,3-Dihydroxy-N-methylacridone | 242.08104 | 172.08 (1), 196.08 (1), 242.08 (100) |
| 1,3,8-p-Menthatriene | 135.11664 | 53.04 (5), 55.05 (9), 65.04 (2), 67.05 (2), 69.07 (3), 79.05 (15), 81.07 (3), 91.05 (38), 93.07 (57), 95.09 (1), 105.07 (19), 106.08 (1), 107.09 (100), 117.07 (1), 119.09 (13), 120.09 (5), 133.1 (1), 135.12 (50) |
| 10-Deoxygeniposidic acid | 357.11929 | 55.02 (2), 57.03 (11), 59.01 (3), 69 (1), 71.01 (5), 72.99 (2), 75.01 (2), 83.01 (3), 84.02 (4), 85.03 (100), 87.01 (1), 101.02 (1), 103 (1), 115 (1), 129.02 (8), 357.12 (3) |
| 10-OPDA | 293.21094 | 55.02 (2), 55.05 (13), 57.07 (2), 67.05 (24), 69.07 (20), 79.05 (12), 81.07 (29), 83.05 (7), 83.09 (4), 85.06 (2), 93.07 (19), 95.05 (7), 95.09 (16), 97.07 (2), 107.05 (7), 107.09 (10), 109.06 (6), 109.1 (9), 121.06 (9), 121.1 (7), 123.08 (5), 123.12 (2), 133.06 (2), 133.1 (17), 135.08 (4), 135.12 (4), 137.1 (3), 147.08 (2), 147.12 (12), 149.1 (3), 149.13 (2), 151.11 (5), 159.12 (7), 161.1 (3), 161.13 (2), 163.11 (4), 173.13 (4), 175.11 (2), 191.14 (4), 207.14 (2), 219.14 (4), 229.2 (2), 239.18 (2), 247.21 (4), 257.19 (14), 275.2 (100), 293.21 (40) |
| 12-OPDA | 293.21063 | 55.02 (8), 55.05 (3), 55.05 (30), 57.03 (5), 67.05 (47), 69.03 (5), 69.07 (21), 71.05 (5), 79.05 (45), 81.03 (5), 81.07 (45), 83.05 (7), 83.09 (18), 93.07 (34), 95.05 (14), 95.09 (33), 97.06 (7), 97.1 (13), 107.09 (65), 109.06 (16), 109.1 (17), 111.08 (4), 119.09 (26), 121.06 (4), 121.1 (20), 123.08 (5), 123.12 (7), 125.1 (13), 133.1 (23), 135.08 (7), 135.12 (6), 137.1 (11), 139.11 (5), 147.12 (12), 149.1 (20), 151.11 (5), 159.12 (8), 161.13 (5), 163.11 (11), 173.13 (5), 229.2 (5), 239.18 (5), 247.21 (4), 257.19 (15), 275.2 (100), 293.21 (74) |
| 12-Oxo-9(Z)-dodecenoic acid | 211.13396 | 167.14 (1), 193.12 (3), 211.13 (100) |
| 13-HOTrE | 295.22659 | 53.04 (13), 55.02 (9), 55.05 (38), 57.03 (5), 57.07 (9), 67.05 (79), 69.03 (7), 69.07 (28), 71.09 (41), 79.05 (45), 81.03 (28), 81.07 (63), 83.05 (15), 83.09 (17), 85.06 (5), 85.1 (8), 93.07 (52), 95.05 (23), 95.09 (40), 97.06 (10), 97.1 (16), 99.08 (25), 105.07 (25), 107.09 (33), 109.06 (5), 109.1 (28), 111.08 (5), 113.1 (5), 119.09 (16), 121.1 (18), 123.08 (5), 123.12 (6), 125.1 (13), 133.1 (14), 135.08 (6), 135.12 (19), 137.1 (6), 145.1 (5), 147.12 (15), 149.13 (10), 151.11 (31), 153.13 (4), 161.13 (15), 163.11 (9), 165.13 (13), 173.13 (5), 179.14 (12), 241.19 (8), 259.21 (9), 277.22 (100), 295.23 (55) |
| 13-HpOTrE(r) | 311.22147 | 55.05 (74), 57.03 (24), 57.07 (14), 67.05 (58), 69.07 (87), 71.09 (19), 79.05 (27), 81.07 (58), 83.09 (49), 91.05 (6), 93.07 (31), 95.09 (45), 97.06 (8), 97.1 (31), 107.09 (15), 109.1 (18), 111.12 (7), 121.1 (23), 125.1 (5), 135.12 (10), 137.13 (7), 149.13 (5), 275.2 (10), 293.21 (100) |
| 13-OxoODE | 295.22647 | 53.04 (3), 55.02 (82), 55.05 (31), 57.07 (2), 67.05 (25), 69.03 (2), 69.07 (11), 71.05 (3), 71.09 (4), 79.05 (16), 81.03 (5), 81.07 (31), 83.05 (11), 83.09 (22), 85.07 (2), 91.05 (23), 93.07 (19), 95.05 (5), 95.09 (17), 97.06 (12), 97.1 (3), 99.08 (4), 107.05 (2), 107.09 (12), 109.06 (6), 109.1 (7), 111.08 (100), 113.1 (2), 121.1 (9), 123.08 (3), 123.12 (3), 125.1 (2), 127.11 (1), 133.1 (24), 135.12 (4), 137.1 (2), 137.13 (2), 139.11 (7), 147.12 (14), 149.1 (8), 149.13 (13), 151.11 (14), 161.13 (4), 163.11 (3), 165.13 (18), 175.11 (4), 175.15 (2), 177.13 (1), 179.14 (6), 193.12 (2), 221.15 (2), 231.21 (2), 241.2 (6), 249.22 (3), 259.21 (10), 277.22 (79), 295.23 (53) |
| 13(S)-HODE | 295.228 | 57.03 (1), 59.01 (2), 113.1 (2), 195.14 (19), 277.22 (16), 295.23 (100) |
| 14,15-Dehydrocrepenynic acid | 277.21613 | 53.04 (7), 55.02 (12), 55.05 (17), 57.07 (5), 67.05 (29), 69.07 (14), 71.09 (10), 79.05 (30), 81.03 (4), 81.07 (35), 83.05 (6), 83.09 (10), 91.05 (20), 93.07 (46), 95.05 (5), 95.09 (23), 97.06 (7), 99.08 (8), 105.07 (18), 107.05 (4), 107.09 (24), 109.07 (4), 109.1 (12), 111.08 (10), 119.09 (13), 121.1 (13), 123.08 (5), 131.09 (5), 133.1 (12), 135.12 (17), 145.1 (7), 147.12 (14), 149.13 (11), 151.11 (6), 161.13 (8), 163.11 (6), 163.15 (5), 165.13 (6), 175.15 (4), 207.14 (4), 235.17 (5), 241.2 (4), 249.22 (6), 259.21 (10), 277.22 (100) |
| 16-Hydroxypalmitate | 271.22794 | 225.22 (66), 253.22 (1), 271.23 (100) |
| 17alpha-Hydroxypregnenolone | 333.24207 | 53.04 (1), 55.05 (12), 67.05 (4), 69.03 (2), 69.07 (5), 79.05 (8), 81.07 (8), 83.09 (1), 93.07 (13), 95.09 (7), 99.04 (1), 107.09 (12), 109.06 (6), 121.06 (17), 121.1 (4), 123.08 (2), 123.12 (1), 147.12 (1), 161.13 (2), 163.11 (5), 165.13 (3), 175.15 (1), 177.13 (11), 191.14 (2), 205.16 (1), 243.21 (1), 261.22 (7), 271.21 (1), 289.22 (12), 333.24 (100) |
| 1H-indene-3-carboxamide | 160.07542 | 115.05 (12), 132.08 (16), 133.07 (3), 142.06 (3), 143.07 (2), 146.06 (1), 159.07 (3), 160.08 (100) |
| 2-(4'-Methylthio)butylmalic acid | 235.06451 | 59.01 (100), 71.01 (19), 85.03 (14), 87.01 (80), 101.02 (11), 113.02 (16), 175.04 (92), 235.06 (33) |
| 2-(Acetamidomethylene)succinate | 188.05513 | 56.05 (19), 60.04 (8), 66.03 (4), 68.05 (2), 69.03 (1), 70.07 (50), 82.03 (2), 82.07 (5), 84.04 (9), 85.03 (3), 86.06 (1), 87.01 (3), 88.04 (2), 96.04 (1), 97.03 (1), 98.06 (3), 100.04 (3), 100.08 (6), 102.05 (12), 110.02 (2), 114.05 (54), 116.07 (25), 128.07 (19), 142.05 (100), 152.03 (2), 170.04 (23) |
| 2-Dehydro-3-deoxy-L-arabinonate | 147.02998 | 57.03 (12), 59.01 (4), 71.01 (94), 72.99 (2), 73.03 (3), 85.03 (32), 87.01 (43), 101.02 (2), 103.04 (13), 115 (100), 129.02 (7), 147.03 (17) |
| 2-Furoate | 111.00871 | 67.02 (50), 111.01 (100) |
| 2-Hydroxy-6-ketononatrienedioate | 213.03931 | 53 (1), 53.04 (23), 55.02 (3), 57.03 (4), 67.02 (2), 67.05 (3), 69 (20), 69.03 (48), 71.05 (16), 79.05 (2), 81.03 (58), 82.04 (2), 83.01 (2), 83.05 (1), 85.03 (34), 87.01 (18), 95.05 (9), 97.03 (10), 99.04 (31), 109.03 (42), 110.04 (17), 123.04 (9), 125.02 (13), 126.03 (1), 127.04 (100), 139.04 (1), 141.05 (1), 151.04 (10), 153.02 (13), 169.05 (1), 195.03 (5), 213.04 (53) |
| 2-Hydroxy-6-oxo-(2'-aminophenyl)-hexa-2,4-dienoate | 234.07576 | 87.01 (1), 103.05 (9), 118.07 (8), 120.08 (100), 130.07 (2), 131.05 (2), 144.08 (7), 146.06 (14), 148.08 (2), 188.07 (14), 206.08 (23), 216.07 (2), 234.08 (3) |
| 2-Hydroxy-6-oxo-6-(2-carboxyphenyl)-hexa-2,4-dienoate | 263.05447 | 53 (1), 53.04 (3), 55.02 (5), 57.03 (2), 65.04 (1), 69 (8), 69.03 (48), 71.05 (1), 79.02 (1), 79.05 (12), 81.03 (3), 83.01 (1), 83.05 (1), 85.03 (5), 91.05 (3), 95.05 (3), 97.03 (1), 99.01 (1), 99.04 (6), 103.05 (2), 105.03 (1), 105.07 (4), 107.05 (31), 109.03 (1), 111.01 (2), 111.04 (14), 115.05 (4), 121.03 (1), 123.04 (1), 131.05 (3), 133.03 (1), 135.04 (78), 137.02 (1), 147.04 (5), 149.02 (1), 149.06 (1), 151.04 (2), 161.06 (3), 163.04 (1), 171.04 (5), 175.04 (5), 177.02 (2), 177.05 (3), 179.03 (7), 189.05 (6), 191.03 (5), 193.05 (6), 199.04 (2), 201.06 (1), 203.03 (20), 207.06 (2), 217.05 (30), 221.04 (18), 227.03 (1), 235.06 (2), 245.04 (100), 263.05 (81) |
| 2-Hydroxy-6-oxo-6-(2-hydroxyphenoxy)-hexa-2,4-dienoate | 251.05476 | 53.04 (6), 55.02 (2), 77.04 (1), 83.01 (5), 85.03 (1), 95.05 (2), 101.02 (1), 105.03 (100), 111.01 (7), 123.04 (3), 129.02 (3), 251.05 (4) |
| 2-Maleylacetate | 159.02872 | 55.02 (2), 55.05 (4), 57.03 (2), 61.03 (2), 69 (5), 71.01 (1), 73.03 (2), 85.03 (1), 87.01 (100), 105.02 (11), 113.02 (1), 117.02 (77), 141.02 (3), 159.03 (16) |
| 2-Oxoglutarate | 145.01416 | 57.03 (32), 71.01 (15), 73.03 (24), 83.01 (22), 99.01 (49), 101.02 (100), 145.01 (20) |
| 2-Oxopimelate | 173.04558 | 57.03 (46), 59.01 (5), 67.02 (5), 69.03 (4), 71.01 (4), 72.99 (20), 83.01 (6), 83.05 (38), 85.03 (100), 85.07 (9), 101.02 (6), 111.05 (72), 127.04 (5), 129.06 (23), 155.04 (48), 173.05 (89) |
| 2-Phenylacetamide | 136.07556 | 65.04 (1), 91.05 (100), 107.05 (4), 109.07 (1), 118.07 (3), 119.05 (30), 136.08 (25) |
| 2,4-Bis(acetamido)-2,4,6-trideoxy-beta-L-altropyranose | 247.12883 | 56.05 (10), 58.07 (22), 72.08 (100), 84.04 (72), 84.08 (10), 118.09 (20), 148.06 (14), 183.11 (38), 229.12 (32), 247.13 (26) |
| 2,4-Diacetamido-2,4,6-trideoxy-beta-L-gulose | 247.12863 | 56.05 (2), 70.07 (4), 72.08 (100), 84.04 (64), 102.06 (2), 118.09 (52), 130.05 (11), 136.08 (3), 156.1 (3), 229.12 (1), 247.13 (13) |
| 2,4-Diacetamido-2,4,6-trideoxy-D-mannopyranose | 56.04992 | 56.05 (1), 70.03 (9), 70.07 (1), 72.08 (1), 73.03 (3), 84.08 (3), 85.04 (6), 88.04 (100), 96.08 (2), 98.02 (6), 100.04 (2), 110.02 (22), 124.04 (4), 141.1 (36), 156.1 (8), 169.1 (1), 183.11 (13), 187.11 (22), 201.12 (69), 211.11 (1), 229.12 (11), 247.13 (21) |
| 2,5-Dioxopentanoate | 129.01932 | 85.03 (100), 129.02 (7) |
| 3-(2-Carboxyethenyl)-cis,cis-muconate | 213.03915 | 53 (1), 53.04 (23), 55.02 (3), 55.05 (1), 57.03 (3), 61.03 (1), 67.02 (4), 67.05 (3), 69 (23), 69.03 (49), 79.05 (2), 81.03 (59), 82.04 (1), 83.01 (2), 83.05 (2), 85.03 (35), 87.01 (16), 95.05 (9), 97.03 (11), 99.04 (31), 107.05 (1), 109.03 (42), 110.04 (18), 123.04 (8), 125.02 (15), 127.04 (100), 151.04 (9), 153.02 (12), 167.03 (1), 169.05 (1), 195.03 (5), 213.04 (47) |
| 3-(β-D-Glucopyranosyloxy)-5,7-dihydroxy-2-(3-hydroxy-4-methoxyphenyl)-8-methoxy-4H-1-benzopyran-4-one | 509.12772 | 61.03 (7), 73.03 (2), 85.03 (6), 91.04 (3), 97.03 (3), 127.04 (1), 169.01 (1), 287.05 (1), 301.03 (10), 315.05 (3), 331.04 (1), 332.05 (35), 347.08 (100) |
| 3-Amino-3-(4-hydroxyphenyl)propanoate | 182.08101 | 91.05 (34), 95.05 (14), 103.05 (1), 107.05 (1), 109.06 (1), 118.07 (1), 119.05 (40), 120.08 (1), 121.06 (1), 123.04 (52), 136.08 (100), 147.04 (18), 165.05 (31), 182.08 (3) |
| 3-Butynoate | 85.02833 | 55.02 (4), 57.03 (20), 67.02 (1), 85.03 (100) |
| 3-Cyano-L-alanine | 113.03568 | 86.02 (1), 96.01 (26), 97.02 (2), 113.04 (100) |
| 3-Deoxy-D-manno-octulosonate | 237.06152 | 57.03 (2), 59.01 (1), 71.01 (2), 72.99 (4), 75.01 (1), 85.03 (5), 87.01 (100), 99.01 (1), 101.02 (1), 129.02 (19), 147.03 (2), 237.06 (6) |
| 3-Dimethylallyl-4-hydroxybenzaldehyde | 191.10635 | 55.05 (1), 79.05 (1), 91.05 (5), 95.05 (1), 95.09 (1), 105.07 (1), 107.05 (2), 117.07 (6), 121.06 (47), 131.05 (16), 135.04 (1), 135.08 (8), 145.06 (9), 145.1 (1), 146.04 (2), 147.04 (2), 148.05 (3), 149.06 (100), 163.08 (13), 163.11 (2), 173.06 (2), 191.11 (10) |
| 3-hydroxy-5,6,7,8,4'-pentamethoxyflavone | 389.12244 | 137.02 (1), 139.04 (1), 153.02 (1), 165.05 (12), 181.01 (1), 299.05 (1), 303.08 (1), 313.07 (1), 329.07 (1), 331.08 (7), 341.07 (2), 343.08 (1), 344.05 (4), 345.1 (2), 357.1 (1), 359.08 (91), 373.09 (5), 374.1 (8), 389.12 (100) |
| 3-Methoxytyramine | 168.10162 | 91.05 (27), 107.05 (5), 109.06 (4), 119.05 (14), 121.06 (7), 135.07 (29), 150.09 (100) |
| 3-Methylindolepyruvate | 218.08128 | 144.08 (1), 159.07 (7), 173.08 (16), 174.09 (15), 200.07 (4), 218.08 (100) |
| 3-Methylpyrrole-2,4-dicarboxylic acid | 170.04456 | 54.03 (2), 56.05 (11), 114.05 (92), 124.04 (1), 142.05 (100), 170.04 (4) |
| 3-O-Methylquercetin | 317.06488 | 92.03 (3), 93.03 (2), 121.03 (1), 139.04 (1), 151.04 (1), 153.02 (16), 165.02 (1), 229.05 (6), 245.04 (3), 246.05 (3), 257.04 (4), 273.04 (3), 274.05 (6), 285.04 (7), 302.04 (21), 317.07 (100) |
| 3-Ureidopropionate | 133.06071 | 60.04 (1), 70.03 (5), 74.02 (100), 87.06 (38), 88.04 (9), 99.01 (1), 116.03 (9), 133.06 (5) |
| 3,4-Methylenedioxycinnamaldehyde | 177.05429 | 91.05 (3), 103.05 (2), 106.04 (4), 117.03 (73), 134.04 (11), 135.04 (6), 145.03 (100), 149.06 (25), 162.03 (3), 163.04 (4), 177.05 (36) |
| 3,6,7,4'-tetramethoxyflavone | 359.11197 | 113.02 (1), 133.06 (7), 135.04 (4), 283.06 (1), 301.07 (4), 326.08 (1), 329.07 (100), 344.09 (18), 359.11 (90) |
| 3,7-Di-O-methylquercetin | 331.0809 | 99.01 (1), 151.04 (3), 245.04 (8), 273.04 (13), 288.06 (1), 301.03 (13), 316.06 (89), 331.08 (100) |
| 3,7,4'-Tri-O-methylquercetin | 345.09641 | 108.02 (1), 149.06 (4), 151.04 (3), 269.04 (3), 283.06 (1), 284.07 (12), 287.05 (22), 297.04 (2), 301.07 (1), 312.06 (51), 315.05 (23), 329.07 (1), 330.07 (11), 345.1 (100) |
| 3',4'-Dimethoxyluteolin | 315.08585 | 108.02 (10), 121.03 (4), 136.02 (6), 226.06 (6), 254.06 (45), 257.04 (1), 271.06 (2), 282.05 (37), 285.04 (1), 299.05 (2), 300.06 (12), 315.09 (100) |
| 4-Acetamidobutanoate | 146.02968 | 58.07 (6), 72.08 (100), 82.07 (2), 84.04 (3), 84.08 (12), 86.06 (1), 100.08 (4), 130.05 (1), 146.08 (1) |
| 4-Coumarate | 165.05444 | 53.04 (4), 65.04 (1), 67.05 (1), 79.05 (1), 81.03 (1), 81.07 (1), 91.05 (21), 93.07 (2), 95.05 (62), 103.05 (3), 109.06 (4), 119.05 (55), 121.06 (2), 123.04 (100), 133.03 (2), 137.06 (3), 147.04 (30), 165.05 (5) |
| 4-Guanidinobutanal | 130.09738 | 53 (1), 56.05 (7), 60.06 (33), 70.07 (57), 71.05 (68), 84.04 (100), 84.08 (96), 88.08 (5), 112.09 (1), 130.1 (29) |
| 4-Hydroxycoumarin | 163.0388 | 53.04 (10), 79.05 (5), 81.03 (1), 92.03 (3), 95.05 (4), 105.03 (7), 107.05 (2), 135.04 (25), 163.04 (100) |
| 4-Hydroxystyrene | 121.06459 | 51.02 (1), 53.04 (29), 55.02 (2), 65.04 (1), 77.04 (2), 81.03 (2), 91.05 (55), 93.07 (55), 95.05 (12), 102.05 (1), 103.05 (75), 121.06 (100) |
| 4-Isopropylbenzaldehyde | 149.09599 | 65.04 (42), 79.05 (20), 91.05 (26), 93.07 (100), 105.03 (54), 105.07 (9), 107.05 (21), 121.1 (27), 149.1 (5) |
| 4-Methylene-L-glutamate | 158.04578 | 72.01 (1), 86.02 (1), 96.05 (21), 97.03 (1), 112.04 (100), 114.06 (21), 158.05 (26) |
| 4-Methylene-L-glutamine | 159.07632 | 68.05 (1), 70.07 (100), 71.05 (1), 86.06 (3), 96.04 (1), 113.07 (43), 114.06 (11), 115.09 (3), 116.07 (1), 159.08 (13) |
| 4-Methylumbelliferone | 177.05434 | 91.05 (5), 103.05 (3), 119.05 (2), 121.06 (1), 135.04 (14), 147.04 (1), 149.06 (63), 162.03 (4), 163.04 (10), 177.05 (100) |
| 4-oxo-9Z,11Z,13E,15E-octadecatetraenoic acid | 291.19522 | 53.04 (5), 55.02 (12), 55.05 (27), 57.03 (2), 59.05 (1), 65.04 (2), 67.05 (25), 69.03 (3), 69.07 (9), 71.05 (2), 79.05 (46), 81.03 (3), 81.07 (46), 83.05 (4), 83.09 (11), 85.06 (1), 91.05 (34), 93.07 (26), 95.05 (15), 95.09 (15), 97.06 (2), 97.1 (12), 105.07 (21), 107.05 (12), 107.09 (16), 109.06 (6), 109.1 (5), 111.08 (3), 119.09 (54), 121.06 (21), 121.1 (9), 123.08 (5), 125.1 (11), 133.06 (7), 133.1 (6), 135.04 (1), 135.08 (12), 135.12 (2), 137.1 (2), 145.1 (26), 147.08 (32), 147.12 (2), 149.1 (8), 151.08 (3), 153.09 (1), 159.12 (6), 161.1 (11), 163.11 (4), 171.12 (4), 173.13 (1), 175.11 (3), 177.09 (1), 185.13 (3), 187.11 (1), 189.13 (2), 199.15 (1), 207.14 (2), 213.13 (2), 219.14 (1), 223.13 (3), 227.18 (2), 231.14 (1), 237.16 (1), 245.19 (3), 249.15 (15), 255.17 (6), 273.19 (45), 291.2 (100) |
| 4-Oxoproline | 130.0499 | 56.05 (4), 70.07 (7), 84.04 (100), 85.03 (4), 102.06 (1), 130.05 (12) |
| 4,5-Dioxopentanoate | 129.01941 | 55.02 (1), 71.01 (1), 85.03 (100), 129.02 (4) |
| 4'-hydroxy-5,6,7,-trimethoxyflavone | 329.10159 | 135.04 (5), 136.02 (5), 154.03 (5), 240.08 (4), 268.07 (41), 271.06 (1), 285.08 (2), 296.07 (38), 299.05 (1), 314.08 (11), 329.1 (100) |
| 4'-hydroxy-5,6,7,3',-tetramethoxyflavone | 359.1123 | 108.02 (5), 136.02 (2), 137.02 (1), 147.04 (2), 154.03 (3), 163.08 (4), 165.05 (4), 270.09 (3), 298.08 (37), 299.06 (1), 301.07 (1), 315.09 (3), 326.08 (31), 329.07 (2), 343.08 (12), 344.09 (10), 359.11 (100) |
| 4'-hydroxy-5,6,7,8,-tetramethoxyflavone | 359.11197 | 121.03 (1), 165.05 (1), 167.03 (3), 179.03 (1), 273.08 (4), 285.08 (1), 298.08 (1), 299.06 (3), 301.07 (27), 313.07 (2), 315.09 (3), 326.08 (1), 329.07 (21), 344.09 (48), 359.11 (100) |
| 4'-O-Methylnorbelladine | 274.14362 | 103.05 (1), 146.06 (1), 176.07 (4), 224.11 (13), 226.12 (1), 241.11 (3), 256.13 (51), 274.14 (100) |
| 5-Acetamidopentanoate | 160.09659 | 160.1 (100) |
| 5-Dehydro-4-deoxy-D-glucarate | 191.01953 | 57.03 (5), 85.03 (29), 87.01 (46), 111.01 (100), 129.02 (5), 155 (1), 173.01 (2), 191.02 (11) |
| 5-Guanidino-2-oxopentanoate | 174.08731 | 55.05 (1), 60.06 (1), 68.05 (3), 69.03 (1), 70.07 (18), 71.05 (2), 86.06 (1), 114.05 (100), 115.04 (1), 128.03 (1), 128.07 (28), 174.09 (6) |
| 5-Hydroxy-2-(3-hydroxy-4,5-dimethoxyphenyl)-7,8-dimethoxy-4H-1-benzopyran-4-one | 375.10699 | 149.06 (5), 151.04 (3), 299.05 (2), 313.07 (2), 314.08 (2), 317.07 (3), 327.05 (25), 329.06 (1), 342.07 (3), 345.06 (62), 360.08 (9), 375.11 (100) |
| 5-hydroxy-3,6,7,8,3',4'-hexamethoxyflavone | 419.13287 | 165.05 (7), 169.01 (1), 178.06 (1), 197.01 (1), 315.05 (1), 343.08 (3), 361.09 (10), 386.1 (1), 389.09 (60), 403.1 (3), 404.11 (6), 419.13 (100) |
| 5-hydroxy-3,7,3',4'-tetramethoxyflavone | 359.11185 | 108.02 (3), 136.02 (1), 137.02 (1), 153.02 (1), 163.08 (4), 165.05 (4), 270.09 (2), 283.06 (1), 298.08 (25), 301.07 (12), 315.09 (2), 326.08 (40), 329.07 (13), 343.08 (5), 344.09 (10), 359.11 (100) |
| 5-hydroxy-6,7,4'-trimethoxyflavone | 329.10147 | 69 (2), 99.04 (3), 108.02 (7), 119.05 (3), 121.03 (7), 127.04 (6), 132.06 (6), 133.06 (10), 135.04 (4), 136.02 (3), 139 (2), 140.01 (1), 154.03 (7), 183.03 (14), 187.04 (2), 240.08 (4), 268.07 (43), 271.06 (3), 283.06 (3), 285.07 (1), 296.07 (35), 299.06 (2), 301.07 (4), 314.08 (17), 329.1 (100) |
| 5-hydroxy-7,8,3',4'-tetramethoxyflavone | 359.11197 | 108.02 (4), 137.02 (1), 147.04 (2), 154.03 (1), 162.07 (14), 163.08 (5), 165.05 (4), 270.09 (2), 283.06 (1), 298.08 (33), 301.07 (7), 315.09 (1), 326.08 (42), 329.07 (7), 343.08 (4), 344.09 (12), 359.11 (100) |
| 5-Hydroxy-L-tryptophan | 221.09187 | 130.07 (63), 132.04 (41), 148.08 (3), 157.08 (32), 158.06 (77), 175.09 (100), 176.07 (2), 186.05 (2), 203.08 (11), 204.07 (5), 221.09 (24) |
| 5-Hydroxyconiferaldehyde | 195.06482 | 91.05 (1), 106.04 (1), 117.03 (43), 121.06 (1), 134.04 (5), 135.04 (7), 145.03 (75), 149.06 (19), 162.03 (1), 163.04 (8), 177.05 (100), 195.06 (1) |
| 5-Methoxy-N,N-dimethyltryptamine | 219.14909 | 58.07 (5), 86.1 (13), 117.06 (1), 132.08 (5), 160.08 (100), 219.15 (5) |
| 5-Methoxytryptamine | 191.11777 | 117.06 (1), 118.07 (4), 130.07 (13), 132.04 (11), 132.08 (19), 134.06 (1), 142.07 (6), 148.08 (3), 160.08 (100), 191.12 (27) |
| 5-Methylfurfural | 111.04388 | 53.04 (1), 55.02 (1), 55.05 (1), 55.05 (20), 65.04 (3), 83.05 (3), 111.04 (100) |
| 5,6,7,3',4'-pentamethoxyflavanone | 375.14346 | 69 (7), 69.03 (11), 148.05 (2), 150.03 (27), 151.04 (1), 151.08 (2), 161.06 (1), 163.08 (8), 164.08 (1), 167.03 (2), 168.04 (15), 191.07 (29), 193.05 (1), 196.04 (27), 211.06 (100), 286.08 (1), 375.14 (68) |
| 5,6,7,4'-tetramethoxyflavanone | 345.13281 | 94.04 (1), 122.04 (2), 133.06 (1), 137.02 (1), 139.04 (1), 150.03 (4), 151.04 (1), 161.06 (1), 167.03 (7), 168.04 (1), 181.01 (2), 196.04 (16), 211.06 (100), 345.13 (21) |
| 5,7-dihydroxy-3,6-dimethoxyflavone | 315.08603 | 108.02 (8), 119.05 (5), 121.03 (3), 136.02 (5), 154.03 (7), 254.06 (41), 271.06 (2), 282.05 (40), 285.04 (1), 299.05 (2), 300.06 (13), 315.09 (100) |
| 5,7-dihydroxy-6,8,3',4'-tetramethoxyflavone | 373.09296 | 132.02 (7), 147.05 (5), 300.03 (12), 315.05 (3), 328.02 (51), 343.05 (89), 358.07 (75), 373.09 (100) |
| 5,7-Dimethoxycoumarin | 207.0649 | 55.02 (1), 69.03 (1), 77.04 (3), 79.05 (1), 91.05 (93), 93.03 (2), 103.05 (2), 107.05 (1), 119.05 (90), 121.03 (3), 122.04 (1), 135.04 (3), 136.05 (2), 145.03 (2), 146.04 (6), 147.04 (75), 149.02 (10), 150.03 (1), 163.04 (1), 164.05 (6), 174.03 (6), 175.04 (100), 177.02 (2), 179.03 (2), 179.07 (9), 192.04 (24), 207.07 (68) |
| 5,8-dihydroxy-6,7,4'-trimethoxyflavone | 345.0961 | 57.03 (6), 85.03 (20), 109.03 (2), 121.03 (1), 125.02 (3), 149.02 (1), 151.04 (1), 153.02 (2), 171.03 (3), 177.05 (1), 195.03 (100), 213.04 (3), 327.09 (1), 345.1 (4) |
| 5'-Methylthioadenosine | 298.09671 | 61.01 (5), 75.03 (2), 97.03 (1), 136.06 (100), 145.03 (1), 163.04 (1), 298.1 (3) |
| 5S,8R-DiHODE | 311.22293 | 57.03 (2), 139.11 (18), 171.1 (85), 183.1 (6), 201.11 (13), 293.21 (26), 311.22 (100) |
| 6-Acetamido-2-oxohexanoate | 188.09128 | 55.05 (16), 56.05 (2), 60.04 (1), 70.07 (100), 82.06 (2), 83.05 (2), 84.04 (1), 84.08 (2), 98.1 (8), 100.08 (4), 101.06 (2), 114.05 (2), 116.07 (42), 128.07 (8), 142.05 (1), 142.09 (10), 146.08 (7), 188.09 (4) |
| 6-Amino-2-oxohexanoate | 146.0808 | 58.07 (7), 72.08 (100), 84.08 (11) |
| 6-hydroxy-5,7,8,4'-tetramethoxyflavone | 359.11218 | 108.02 (5), 137.02 (1), 147.04 (2), 154.03 (2), 163.08 (4), 165.05 (3), 183.03 (1), 270.09 (2), 298.08 (35), 299.06 (1), 301.07 (1), 315.09 (3), 326.08 (30), 329.07 (2), 343.08 (11), 344.09 (10), 359.11 (100) |
| 6,7-Dimethoxycoumarin | 207.06491 | 77.04 (3), 79.05 (1), 91.05 (97), 93.03 (1), 103.05 (1), 105.07 (1), 107.05 (1), 118.04 (7), 119.05 (93), 121.03 (2), 133.03 (2), 134.04 (1), 135.04 (3), 136.05 (3), 145.03 (2), 146.04 (7), 147.04 (75), 149.02 (12), 150.03 (1), 162.03 (1), 163.04 (1), 164.05 (6), 174.03 (6), 175.04 (100), 177.02 (1), 179.07 (7), 192.04 (24), 207.07 (59) |
| 6'-Hydroxy-3,4,5,2',4'-pentamethoxychaclone | 375.14297 | 69.03 (2), 95.05 (2), 108.02 (2), 109.03 (5), 111.04 (2), 121.03 (6), 122.04 (15), 123.04 (4), 125.02 (5), 137.02 (8), 138.03 (2), 139.04 (9), 140.05 (2), 148.05 (3), 150.03 (24), 151.04 (10), 151.08 (3), 153.02 (17), 155.03 (6), 163.08 (9), 165.02 (3), 165.05 (1), 166.03 (2), 167.03 (48), 183.06 (2), 191.07 (32), 333.13 (2), 375.14 (100) |
| 7-Deoxyloganetate | 199.09622 | 55.05 (8), 69.03 (1), 69.07 (1), 73.03 (1), 83.05 (3), 83.09 (5), 111.04 (100), 111.08 (1), 127.08 (1), 137.06 (1), 155.07 (15), 199.1 (2) |
| 7-Hydroxy-2',4',5'-trimethoxyisoflavone | 329.10153 | 329.1 (100) |
| 7-Methoxycoumarin | 177.05432 | 63.02 (1), 89.04 (43), 91.05 (2), 103.05 (1), 106.04 (3), 117.03 (74), 121.06 (1), 134.04 (10), 135.04 (6), 145.03 (100), 149.06 (25), 162.03 (2), 163.04 (4), 177.05 (37) |
| 8-[(1R,2R)-3-Oxo-2-{(Z)-pent-2-enyl}cyclopentyl]octanoate | 295.22626 | 53 (1), 53.04 (5), 55.02 (5), 55.05 (22), 57.03 (2), 57.07 (8), 59.05 (1), 65.04 (1), 67.05 (33), 69.03 (2), 69.07 (18), 71.05 (3), 71.09 (4), 79.05 (21), 81.03 (16), 81.07 (30), 83.05 (9), 83.09 (8), 85.06 (2), 85.1 (12), 93.07 (26), 95.05 (13), 95.09 (25), 97.06 (5), 97.1 (9), 99.08 (3), 105.07 (10), 107.05 (2), 107.09 (14), 109.06 (4), 109.1 (13), 111.08 (2), 113.1 (10), 119.09 (9), 121.06 (1), 121.1 (9), 123.08 (3), 123.12 (3), 125.1 (3), 133.1 (6), 135.08 (1), 135.12 (4), 137.1 (8), 139.11 (4), 145.1 (2), 147.12 (4), 149.1 (3), 149.13 (3), 151.11 (7), 153.13 (1), 155.11 (1), 161.13 (3), 163.11 (2), 163.15 (1), 165.13 (23), 167.14 (1), 173.13 (1), 175.15 (1), 179.14 (3), 181.12 (2), 189.13 (1), 207.14 (1), 221.15 (1), 231.21 (1), 241.2 (1), 249.22 (5), 259.21 (3), 277.22 (56), 295.23 (100) |
| 8-Amino-7-oxononanoate | 188.12779 | 55.05 (12), 70.07 (78), 98.1 (7), 128.07 (2), 142.09 (2), 142.12 (43), 188.13 (100) |
| 8-hydroxy-5,6,7,3',4',5'-hexamethoxyflavone | 419.13245 | 165.05 (8), 327.05 (1), 328.06 (14), 343.08 (5), 345.06 (1), 359.04 (1), 361.09 (17), 374.06 (2), 386.1 (1), 389.09 (59), 404.11 (12), 419.13 (100) |
| 9-Hydroxy-12-oxo-10(E),15(Z)-octadecadienoic acid | 309.20724 | 57.03 (1), 59.01 (2), 67.02 (2), 69.03 (1), 71.05 (3), 81.03 (5), 83.05 (28), 84.02 (12), 95.05 (2), 97.03 (1), 97.07 (3), 99.08 (9), 109.07 (3), 111.08 (1), 115.08 (5), 123.08 (1), 125.1 (5), 127.11 (4), 135.08 (1), 137.1 (44), 139.11 (7), 151.11 (4), 153.09 (8), 154.1 (2), 167.11 (3), 171.1 (48), 193.12 (26), 195.14 (2), 209.12 (3), 211.13 (4), 247.21 (7), 263.2 (6), 265.22 (5), 273.19 (2), 291.2 (100), 309.21 (84) |
| 9-Hydroxy-12-oxo-15(Z)-octadecenoic acid | 311.22287 | 57.03 (1), 121.1 (1), 125.1 (3), 127.11 (6), 139.11 (12), 153.09 (1), 155.11 (2), 165.09 (1), 171.1 (69), 183.1 (5), 185.12 (10), 275.2 (4), 293.21 (14), 311.22 (100) |
| 9-KODE | 295.2265 | 53 (1), 53.04 (4), 55.02 (4), 55.05 (26), 57.03 (2), 57.07 (3), 65.04 (1), 67.05 (62), 69.03 (1), 69.07 (32), 71.05 (10), 71.09 (8), 79.05 (30), 81.03 (3), 81.07 (46), 83.05 (7), 83.09 (6), 85.06 (4), 91.05 (15), 93.07 (61), 95.05 (4), 95.09 (100), 97.06 (11), 97.1 (10), 99.08 (6), 107.09 (20), 109.06 (2), 109.1 (12), 111.08 (2), 111.12 (2), 121.1 (34), 123.08 (2), 123.12 (5), 125.1 (4), 133.1 (12), 135.12 (17), 137.1 (5), 139.11 (15), 147.12 (7), 149.1 (2), 149.13 (7), 151.11 (5), 161.13 (6), 163.11 (3), 173.13 (2), 175.15 (1), 179.14 (3), 187.15 (1), 189.16 (2), 207.17 (2), 231.21 (2), 235.17 (1), 241.19 (5), 259.21 (18), 277.22 (86), 295.23 (12) |
| 9,10-Dihydrojasmonic acid | 213.1483 | 53.04 (1), 55.05 (15), 57.07 (9), 67.05 (3), 69.03 (1), 69.07 (2), 81.07 (4), 83.05 (9), 85.06 (4), 91.05 (3), 93.07 (11), 95.05 (1), 95.09 (11), 97.06 (5), 107.05 (1), 109.06 (4), 109.1 (2), 111.04 (100), 111.08 (3), 113.06 (1), 121.1 (2), 123.08 (1), 125.06 (28), 127.08 (3), 135.12 (22), 137.13 (2), 139.08 (4), 153.13 (6), 163.11 (26), 181.12 (56), 195.14 (4), 213.15 (1) |
| 9,10-EOT | 293.21072 | 53 (1), 53.04 (3), 55.02 (11), 55.05 (1), 55.05 (28), 57.03 (2), 57.07 (1), 65.04 (1), 67.05 (24), 69.03 (2), 69.07 (9), 71.05 (3), 71.09 (8), 79.05 (22), 81.03 (8), 81.07 (33), 83.05 (8), 83.09 (16), 85.03 (2), 85.06 (1), 91.05 (16), 93.07 (19), 95.05 (65), 95.09 (14), 97.06 (4), 97.1 (8), 99.04 (1), 99.08 (6), 105.07 (10), 107.05 (8), 107.09 (17), 109.06 (9), 109.1 (6), 111.04 (5), 111.08 (5), 113.1 (1), 119.09 (9), 121.06 (6), 121.1 (8), 123.08 (4), 123.12 (1), 125.1 (6), 133.1 (6), 135.08 (4), 135.12 (6), 137.1 (3), 139.11 (1), 147.08 (3), 147.12 (5), 149.1 (13), 149.13 (1), 151.11 (2), 153.09 (8), 153.13 (1), 161.1 (1), 161.13 (1), 163.11 (27), 165.13 (9), 167.11 (2), 173.13 (1), 175.11 (1), 177.13 (4), 179.14 (1), 187.11 (1), 193.12 (1), 195.14 (7), 205.12 (3), 223.13 (23), 237.15 (1), 247.21 (3), 257.19 (3), 275.2 (28), 293.21 (100) |
| 9(10)-EpOME | 297.24197 | 55.02 (12), 55.05 (50), 57.07 (8), 67.05 (73), 69.03 (6), 69.07 (56), 71.09 (14), 79.05 (31), 81.07 (74), 83.09 (39), 93.07 (32), 95.09 (67), 97.06 (9), 97.1 (41), 99.08 (13), 107.09 (41), 109.1 (46), 121.1 (11), 123.12 (12), 125.1 (30), 135.08 (10), 135.12 (20), 137.13 (11), 147.12 (10), 153.13 (9), 183.14 (7), 243.21 (13), 261.22 (33), 279.23 (100), 297.24 (15) |
| 9(S)-HPODE | 311.22299 | 113.1 (1), 125.1 (2), 127.11 (3), 139.11 (12), 171.1 (48), 183.1 (3), 185.12 (6), 275.2 (3), 293.21 (37), 311.22 (100) |
| 9(S)-HPOT | 311.22131 | 53 (2), 53.04 (3), 55.02 (9), 55.05 (63), 57.03 (2), 59.05 (1), 67.05 (31), 69.03 (6), 69.07 (14), 71.05 (3), 71.09 (17), 79.05 (24), 81.03 (22), 81.07 (37), 83.05 (18), 83.09 (40), 85.03 (2), 85.06 (2), 91.05 (15), 93.07 (20), 95.05 (31), 95.09 (14), 97.06 (5), 97.1 (34), 99.04 (2), 99.08 (15), 105.07 (13), 107.09 (21), 109.06 (5), 109.1 (13), 111.04 (10), 111.08 (4), 119.09 (10), 121.06 (3), 121.1 (9), 123.08 (1), 125.1 (20), 133.1 (7), 135.08 (4), 135.12 (8), 139.11 (2), 147.12 (6), 149.1 (11), 149.13 (2), 151.11 (1), 153.09 (3), 153.13 (1), 159.12 (4), 163.11 (45), 165.13 (3), 167.11 (5), 171.1 (1), 175.11 (2), 177.13 (5), 181.12 (3), 185.12 (2), 189.11 (11), 191.11 (2), 193.12 (11), 195.14 (18), 211.13 (11), 223.13 (7), 229.2 (2), 239.18 (1), 247.21 (6), 249.22 (3), 257.19 (4), 275.2 (23), 293.21 (100), 311.22 (11) |
| Adenine | 136.06174 | 94.04 (2), 119.04 (7), 136.06 (100) |
| Adenosine | 268.10385 | 136.06 (100), 268.1 (7) |
| Allantoate | 175.04715 | 89.04 (100), 132.04 (17) |
| Anethole | 149.09584 | 65.04 (1), 79.05 (4), 89.04 (1), 91.05 (17), 93.07 (11), 103.05 (1), 105.03 (5), 105.07 (2), 107.05 (7), 109.06 (4), 115.05 (1), 116.06 (4), 120.06 (1), 121.06 (100), 149.1 (21) |
| Anthranilate | 138.05501 | 108.04 (3), 138.06 (100) |
| Apigenin 7-O-neohesperidoside | 577.15625 | 107.01 (1), 117.03 (2), 225.06 (1), 268.04 (3), 269.05 (100), 577.16 (3) |
| Aristolochene | 205.1949 | 55.05 (8), 57.07 (3), 67.05 (6), 69.07 (17), 79.05 (10), 81.07 (54), 83.09 (4), 93.07 (47), 95.09 (28), 97.1 (5), 107.09 (30), 109.1 (23), 111.12 (1), 121.1 (100), 123.12 (11), 135.12 (17), 149.13 (45), 163.15 (3), 205.2 (30) |
| Bannamurpanisin | 403.13757 | 127.04 (3), 163.07 (2), 165.05 (3), 299.09 (1), 312.06 (2), 313.07 (3), 327.09 (7), 345.1 (2), 358.07 (5), 373.09 (80), 387.11 (1), 388.11 (10), 403.14 (100) |
| Benzaldehyde | 107.04894 | 53.04 (9), 55.02 (1), 79.05 (15), 81.03 (1), 91.05 (3), 107.05 (100) |
| Benzyl alcohol | 109.06452 | 51.02 (1), 67.05 (12), 79.05 (2), 81.03 (100), 91.05 (1), 109.06 (5) |
| beta-Caryophyllene | 205.19482 | 53.04 (3), 55.05 (12), 57.07 (7), 67.05 (15), 69.07 (26), 79.05 (14), 81.07 (55), 83.09 (12), 93.07 (53), 95.09 (39), 97.1 (8), 107.09 (43), 109.1 (34), 111.12 (2), 121.1 (100), 123.12 (15), 135.12 (34), 137.13 (3), 149.13 (58), 163.15 (7), 205.2 (45) |
| Bicyclo[3.1.1]hept-2-ene | 95.08533 | 53.04 (3), 55.05 (29), 65.04 (3), 67.05 (91), 80.06 (1), 91.05 (3), 93.07 (15), 95.09 (100) |
| Butanal | 73.06477 | 53 (1), 53.04 (4), 55.05 (100), 58.04 (3), 73.06 (34) |
| Caffeic aldehyde | 165.05426 | 65.04 (1), 79.05 (1), 91.05 (24), 95.05 (1), 119.05 (49), 123.04 (1), 133.03 (1), 147.04 (100), 165.05 (3) |
| Calacorene | 203.17921 | 55.05 (7), 57.07 (2), 67.05 (15), 69.07 (7), 79.05 (5), 81.07 (28), 83.09 (3), 91.05 (7), 93.07 (12), 95.09 (53), 105.07 (47), 119.09 (42), 121.1 (16), 131.09 (3), 133.1 (33), 145.1 (3), 147.12 (59), 159.12 (2), 161.13 (15), 175.15 (6), 203.18 (100) |
| Casbene | 273.25729 | 53.04 (1), 55.05 (11), 57.07 (5), 65.04 (1), 67.05 (17), 69.07 (28), 79.05 (8), 81.07 (52), 83.09 (11), 93.07 (28), 95.09 (58), 97.1 (7), 107.09 (45), 109.1 (46), 119.09 (21), 121.1 (39), 123.12 (29), 133.1 (14), 135.12 (24), 137.13 (11), 147.12 (14), 149.13 (36), 151.15 (6), 161.13 (20), 163.15 (21), 165.16 (2), 175.15 (11), 177.16 (14), 189.16 (18), 191.18 (9), 203.18 (13), 217.2 (21), 231.21 (2), 273.26 (100) |
| Chrysoeriol | 301.07022 | 79.05 (1), 121.03 (1), 153.02 (1), 229.05 (1), 257.05 (1), 258.05 (39), 286.05 (54), 301.07 (100) |
| Chrysoeriol-7-O-β-D-glucoside | 463.12247 | 85.03 (1), 109.03 (1), 137.02 (6), 149.06 (1), 151.04 (16), 175.04 (1), 177.02 (2), 192.04 (1), 271.06 (3), 272.07 (5), 282.05 (1), 283.06 (1), 284.07 (2), 285.08 (3), 286.05 (1), 299.06 (3), 300.06 (8), 301.07 (3), 312.06 (10), 313.07 (69), 325.07 (14), 327.09 (14), 328.06 (24), 337.07 (1), 340.06 (2), 341.06 (3), 343.08 (98), 353.06 (2), 355.08 (4), 357.1 (1), 367.08 (22), 373.09 (7), 385.09 (2), 397.09 (16), 409.09 (10), 427.1 (35), 445.11 (62), 463.12 (100) |
| Chrysosplin | 375.10687 | 149.06 (4), 151.04 (3), 313.07 (2), 314.08 (2), 317.07 (2), 327.05 (22), 330.04 (8), 342.07 (2), 345.06 (57), 360.08 (8), 375.11 (100) |
| cis-2,3-Dihydroxy-2,3-dihydro-p-cumate | 199.0963 | 53 (3), 53.04 (1), 53.04 (19), 55.02 (2), 55.05 (72), 57.03 (6), 67.05 (2), 69.03 (29), 69.07 (1), 71.01 (1), 71.05 (30), 73.06 (100), 81.03 (34), 81.07 (2), 83.05 (8), 85.03 (7), 87.04 (1), 91.05 (1), 93.07 (1), 95.05 (2), 95.09 (2), 97.03 (30), 97.06 (2), 99.04 (61), 109.03 (23), 109.06 (2), 111.04 (62), 111.08 (3), 123.04 (1), 123.08 (1), 125.02 (19), 127.04 (46), 139.08 (3), 141.09 (2), 145.05 (2), 153.09 (2), 169.09 (2), 181.09 (4), 199.1 (11) |
| cis-Aconitate | 173.00903 | 67.02 (5), 85.03 (81), 111.01 (100), 129.02 (12), 155 (2), 173.01 (2) |
| cis-Lanceol | 221.18983 | 55.05 (5), 57.07 (3), 67.05 (13), 69.07 (10), 71.05 (3), 79.05 (8), 81.07 (29), 83.09 (3), 91.05 (8), 93.07 (14), 95.09 (44), 97.06 (3), 97.1 (3), 105.07 (30), 107.09 (33), 109.06 (2), 109.1 (33), 111.08 (4), 119.09 (70), 121.1 (18), 123.08 (3), 123.12 (12), 133.1 (28), 135.12 (5), 137.1 (2), 137.13 (3), 147.12 (39), 149.13 (3), 151.11 (3), 161.13 (19), 163.15 (13), 165.13 (2), 175.15 (27), 203.18 (100), 221.19 (44) |
| Cis-Parinaric acid | 275.20157 | 59.01 (4), 231.21 (8), 275.2 (100) |
| Citrate | 191.01967 | 55.02 (2), 57.03 (15), 59.01 (4), 71.01 (6), 72.99 (2), 83.01 (2), 85.03 (100), 87.01 (3), 111.01 (12), 115 (3), 129.02 (2), 147.03 (2), 173.01 (1), 191.02 (14) |
| Colnelenic acid | 291.19681 | 247.21 (1), 291.2 (100) |
| Coniferin | 341.12427 | 57.03 (4), 58.01 (4), 59.01 (100), 71.01 (88), 72.99 (10), 73.03 (23), 75.01 (33), 83.01 (9), 84.02 (2), 85.03 (26), 87.01 (13), 89.02 (23), 99.01 (4), 101.02 (36), 113.02 (25), 119.04 (7), 129.02 (12), 131.04 (3), 143.04 (2), 159.03 (3), 161.05 (3), 177.04 (56), 281.1 (21) |
| Coniferyl alcohol | 181.08574 | 69.03 (1), 81.03 (100), 89.04 (1), 109.03 (8), 117.03 (1), 135.04 (2), 145.03 (1), 163.04 (8), 181.09 (4) |
| Corymbosin | 359.11194 | 121.03 (2), 127.04 (1), 255.06 (1), 269.04 (2), 283.06 (10), 285.04 (2), 301.07 (4), 313.07 (1), 314.04 (6), 315.09 (1), 329.07 (89), 344.09 (13), 359.11 (100) |
| Coumarin | 147.04391 | 65.04 (7), 91.05 (90), 119.05 (100), 147.04 (50) |
| Crepenynate | 279.23184 | 53.04 (1), 55.05 (7), 57.07 (1), 65.04 (2), 67.05 (93), 69.07 (7), 71.05 (1), 79.05 (9), 81.07 (100), 83.05 (2), 83.09 (4), 85.06 (1), 93.07 (7), 95.09 (77), 97.06 (1), 97.1 (2), 105.07 (3), 107.09 (6), 109.06 (1), 109.1 (49), 111.08 (1), 119.09 (2), 121.1 (6), 123.08 (1), 123.12 (31), 133.1 (1), 135.12 (4), 137.1 (1), 137.13 (10), 147.12 (2), 149.1 (1), 149.13 (3), 151.15 (2), 159.12 (1), 161.13 (1), 163.11 (1), 163.15 (2), 173.13 (4), 177.13 (1), 179.14 (1), 181.12 (2), 187.15 (2), 191.14 (1), 195.14 (1), 201.16 (1), 205.16 (1), 209.15 (3), 223.17 (3), 237.19 (1), 243.21 (2), 261.22 (4), 279.23 (51) |
| Cytosine | 112.05041 | 69.04 (6), 94.04 (2), 95.02 (21), 112.05 (100) |
| D-Arginine | 175.11887 | 60.06 (38), 70.07 (100), 71.05 (1), 72.08 (4), 112.09 (4), 116.07 (33), 130.1 (14), 157.11 (1), 158.09 (9), 175.12 (29) |
| D-Cathinone | 150.09087 | 57.06 (2), 91.05 (38), 103.05 (1), 105.07 (1), 107.05 (10), 117.06 (3), 118.06 (1), 119.05 (18), 121.06 (11), 132.08 (3), 134.06 (2), 135.07 (42), 150.09 (100) |
| D-Galactarate | 209.03024 | 55.02 (1), 59.01 (7), 69 (2), 71.01 (29), 72.99 (8), 75.01 (5), 83.01 (2), 85.03 (100), 87.01 (4), 89.02 (9), 101.02 (1), 103 (2), 111.01 (5), 115 (2), 129.02 (6), 133.01 (12), 147.03 (3), 173.01 (2), 191.02 (24), 209.03 (30) |
| D-Galactonate | 195.05067 | 57.03 (6), 59.01 (9), 69 (2), 71.01 (10), 72.99 (11), 75.01 (100), 85.03 (8), 87.01 (17), 89.02 (6), 99.01 (9), 101.02 (3), 105.02 (2), 111.01 (2), 129.02 (37), 159.03 (6), 177.04 (6), 195.05 (79) |
| D-Glucarate | 209.03027 | 55.02 (1), 58.01 (1), 59.01 (7), 69 (2), 71.01 (27), 72.99 (7), 75.01 (4), 83.01 (2), 85.03 (100), 87.01 (4), 89.02 (9), 101.02 (1), 103 (2), 111.01 (4), 115 (2), 129.02 (7), 133.01 (11), 147.03 (3), 173.01 (2), 191.02 (23), 209.03 (25) |
| D-Gluconic acid | 195.05089 | 57.03 (4), 58.01 (1), 59.01 (13), 69 (1), 71.01 (11), 72.99 (8), 75.01 (100), 85.03 (6), 87.01 (14), 89.02 (7), 99.01 (7), 101.02 (2), 105.02 (4), 111.01 (2), 129.02 (33), 141.02 (2), 147.03 (1), 159.03 (7), 177.04 (8), 195.05 (75) |
| D-Glucose | 179.05597 | 55.02 (1), 57.03 (7), 58.01 (5), 59.01 (39), 71.01 (39), 72.99 (14), 73.03 (6), 75.01 (34), 81.03 (6), 83.01 (10), 84.02 (1), 85.03 (53), 87.01 (9), 87.05 (2), 89.02 (37), 97.03 (13), 99.01 (2), 99.05 (3), 101.02 (5), 113.02 (2), 117.02 (1), 125.02 (1), 131.03 (28), 143.03 (3), 159.03 (1), 161.05 (67), 179.06 (100) |
| D-Glucuronate | 193.03488 | 57.03 (11), 59.01 (3), 67.02 (2), 71.01 (2), 72.99 (3), 73.03 (1), 83.01 (1), 85.03 (45), 87.01 (49), 99.01 (1), 103 (2), 111.01 (100), 129.02 (4), 131.03 (6) |
| D-Lysopine | 219.13388 | 55.05 (16), 56.05 (4), 57.06 (1), 58.07 (1), 70.07 (2), 72.08 (100), 73.06 (12), 84.04 (90), 84.08 (1), 85.03 (4), 86.06 (1), 88.04 (1), 90.06 (4), 90.09 (92), 98.06 (2), 102.05 (4), 110.1 (1), 120.07 (1), 130.05 (26), 138.09 (1), 142.05 (1), 155.12 (3), 156.07 (2), 156.1 (20), 173.13 (1), 184.1 (12), 201.12 (32), 202.11 (28), 219.13 (18) |
| D-Mannonate | 195.05096 | 57.03 (4), 59.01 (8), 71.01 (12), 72.99 (9), 75.01 (100), 85.03 (6), 87.01 (20), 89.02 (7), 99.01 (11), 101.02 (3), 111.01 (5), 129.02 (46), 159.03 (4), 177.04 (5), 195.05 (74) |
| D-Saccharic acid | 209.03024 | 55.02 (1), 59.01 (5), 69 (1), 71.01 (26), 72.99 (9), 75.01 (5), 83.01 (2), 85.03 (100), 87.01 (4), 89.02 (6), 101.02 (1), 103 (1), 111.01 (5), 115 (3), 129.02 (8), 133.01 (9), 147.03 (4), 173.01 (2), 191.02 (25), 209.03 (26) |
| D-Xylonate | 165.04036 | 55.02 (2), 57.03 (1), 59.01 (4), 71.01 (5), 72.99 (8), 75.01 (100), 85.03 (2), 87.01 (10), 89.02 (2), 101.02 (1), 105.02 (5), 117.02 (1), 129.02 (13), 147.03 (4), 165.04 (45) |
| D-Xylose | 149.04538 | 59.01 (11), 71.01 (5), 72.99 (25), 73.03 (7), 75.01 (100), 85.03 (3), 87.01 (5), 89.02 (11), 131.04 (8), 149.05 (39) |
| Dehydroepiandrosterone | 289.21552 | 53.04 (2), 55.02 (1), 55.05 (19), 57.03 (1), 67.05 (22), 69.03 (2), 71.05 (1), 81.07 (25), 83.05 (1), 85.06 (1), 93.07 (26), 95.09 (23), 97.06 (1), 97.1 (4), 107.09 (21), 111.08 (1), 121.1 (10), 123.08 (2), 125.1 (1), 137.1 (1), 161.13 (8), 165.13 (6), 175.15 (2), 177.13 (18), 187.15 (4), 191.14 (6), 201.16 (1), 205.16 (2), 229.16 (1), 233.15 (1), 243.21 (2), 247.17 (2), 253.19 (2), 261.22 (17), 271.21 (5), 289.22 (100) |
| delta-Cadinene | 205.19487 | 55.05 (12), 57.07 (5), 67.05 (9), 69.07 (22), 71.09 (1), 79.05 (10), 81.07 (49), 83.09 (6), 91.05 (6), 93.07 (48), 95.09 (30), 97.1 (8), 107.09 (32), 109.1 (23), 111.12 (2), 121.1 (100), 123.12 (11), 135.12 (25), 149.13 (49), 163.15 (4), 205.2 (33) |
| Deoxyadenosine | 252.10907 | 71.05 (1), 73.03 (1), 99.04 (1), 117.05 (2), 119.04 (1), 136.06 (100), 252.11 (2) |
| Deoxyguanosine | 268.10364 | 136.06 (100), 268.1 (5) |
| Dethiobiotin | 215.13884 | 53.04 (1), 55.02 (1), 55.05 (43), 56.05 (3), 58.03 (1), 67.05 (2), 68.05 (2), 69.03 (2), 70.03 (2), 70.07 (6), 72.04 (3), 72.08 (1), 73.03 (4), 81.07 (2), 82.07 (2), 83.09 (36), 84.04 (4), 84.08 (2), 87.06 (24), 93.07 (2), 95.09 (1), 96.04 (1), 98.1 (1), 110.06 (2), 110.1 (97), 112.11 (28), 128.11 (1), 152.11 (22), 154.12 (7), 156.1 (100), 169.13 (41), 170.12 (41), 171.15 (2), 198.11 (90), 215.14 (66) |
| Dihydroactinolide | 181.12207 | 53.04 (2), 55.05 (30), 57.07 (12), 65.04 (1), 67.05 (10), 69.03 (2), 69.07 (4), 71.05 (1), 81.03 (1), 81.07 (9), 83.05 (14), 85.06 (4), 95.05 (3), 97.06 (10), 109.06 (5), 109.1 (3), 111.04 (100), 111.08 (5), 113.06 (4), 121.1 (4), 123.08 (2), 124.05 (1), 125.06 (46), 127.08 (3), 135.08 (25), 135.12 (24), 138.07 (3), 139.08 (4), 153.13 (6), 163.11 (21), 181.12 (44) |
| Dihydromonacolin L acid | 325.23712 | 53 (1), 55.02 (2), 55.05 (14), 57.03 (1), 67.05 (11), 69.03 (3), 71.05 (2), 81.07 (24), 83.05 (1), 83.09 (3), 85.06 (2), 95.05 (7), 95.09 (18), 97.06 (5), 107.05 (1), 109.06 (25), 109.1 (6), 123.12 (2), 135.12 (1), 161.13 (2), 163.15 (2), 191.14 (4), 243.21 (1), 253.19 (1), 261.22 (19), 271.2 (1), 279.23 (6), 289.22 (40), 307.23 (100) |
| Dihydrophaseic acid | 283.15356 | 53.04 (22), 55.02 (5), 55.02 (21), 55.06 (18), 57.03 (40), 65.04 (6), 67.06 (100), 69.07 (20), 71.05 (8), 81.03 (31), 81.07 (71), 83.05 (32), 83.09 (8), 85.03 (4), 85.07 (9), 95.05 (31), 97.03 (4), 97.06 (29), 99.04 (7), 107.05 (96), 109.07 (12), 111.04 (25), 111.08 (8), 123.04 (4), 123.08 (5), 125.06 (6), 181.12 (42), 191.11 (57), 195.07 (16), 197.08 (42), 209.12 (35), 227.13 (86) |
| Dihydrosterigmatocystin | 327.08563 | 65.04 (3), 81.03 (4), 93.03 (2), 121.03 (14), 136.02 (3), 281.08 (1), 299.09 (1), 309.08 (5), 327.09 (100) |
| Diosmetin | 301.07028 | 119.05 (1), 121.03 (4), 258.05 (2), 285.04 (1), 286.05 (98), 301.07 (100) |
| Diosmetin-6-C-glucoside | 461.10913 | 59.01 (14), 71.01 (7), 297.04 (26), 341.07 (100), 371.08 (10), 461.11 (55) |
| Ecgonine | 186.11226 | 84.04 (12), 85.03 (23), 102.05 (12), 112.04 (1), 130.05 (95), 158.12 (1), 186.11 (100) |
| Elemicin | 209.11684 | 55.02 (25), 65.04 (8), 67.05 (100), 79.05 (90), 91.05 (53), 93.07 (81), 95.05 (39), 103.05 (7), 105.07 (24), 107.05 (65), 109.06 (18), 111.04 (31), 117.07 (38), 119.09 (24), 121.06 (18), 125.06 (19), 131.05 (11), 135.08 (9), 145.06 (19), 149.1 (88), 209.12 (57) |
| Ephedrine | 166.12239 | 72.04 (1), 81.03 (1), 91.05 (5), 95.05 (1), 103.05 (7), 107.05 (5), 120.08 (13), 121.06 (100), 133.05 (1), 148.08 (4), 166.12 (11) |
| epsilon-Caprolactam | 114.0913 | 55.05 (1), 67.05 (1), 68.05 (2), 69.07 (10), 72.08 (1), 79.05 (13), 86.1 (1), 96.08 (9), 97.06 (3), 114.09 (100) |
| Ferulate | 195.06487 | 89.04 (20), 91.05 (1), 103.05 (1), 106.04 (1), 117.03 (41), 134.04 (6), 135.04 (6), 145.03 (72), 149.06 (18), 153.05 (1), 162.03 (1), 163.04 (7), 177.05 (100), 195.07 (1) |
| Feruloylputrescine | 265.15445 | 72.08 (7), 89.04 (4), 89.11 (4), 115.09 (1), 117.03 (20), 134.04 (1), 135.04 (1), 145.03 (58), 149.06 (8), 177.05 (100), 248.13 (6), 265.15 (17) |
| Folinic acid | 474.17261 | 70.07 (6), 84.04 (2), 102.05 (1), 104.05 (2), 106.07 (1), 120.04 (12), 132.04 (100), 134.06 (19), 159.05 (1), 194.07 (19), 195.08 (3), 208.08 (1), 327.12 (16), 474.17 (55) |
| Formylanthranilate | 166.04964 | 65.04 (2), 95.05 (5), 111.04 (1), 120.04 (1), 121.03 (100), 148.04 (6), 149.05 (2), 151.04 (1), 166.05 (10) |
| gamma-Glutamyltyramine | 267.13367 | 56.05 (1), 84.04 (22), 91.05 (2), 103.05 (3), 121.06 (100), 130.05 (12), 138.09 (16), 221.13 (4), 250.11 (14), 267.13 (9) |
| gardenin A | 419.13278 | 165.05 (7), 343.08 (7), 361.09 (19), 374.06 (3), 386.1 (2), 389.09 (72), 404.11 (11), 419.13 (100) |
| Gentiopicrin | 355.10361 | 85.03 (3), 149.06 (17), 193.05 (100) |
| Germacrene A acid | 235.16895 | 53.04 (8), 55.05 (2), 67.05 (20), 69.07 (2), 79.05 (30), 81.03 (1), 81.07 (8), 83.09 (1), 93.07 (100), 95.09 (6), 107.09 (16), 109.1 (2), 119.09 (5), 121.1 (23), 133.1 (6), 135.12 (4), 153.09 (1), 175.15 (3), 189.16 (1), 217.16 (2), 235.17 (21) |
| Guanine | 150.04211 | 107.04 (6), 108.02 (14), 133.02 (100), 150.04 (36) |
| Guanosine | 284.09851 | 110.03 (2), 135.03 (4), 152.06 (100), 153.04 (6) |
| HBOA | 166.04959 | 65.04 (2), 72.01 (87), 90.02 (5), 95.05 (5), 111.04 (1), 121.03 (100), 133.03 (3), 138.05 (1), 148.04 (5), 149.05 (2), 151.04 (1), 166.05 (11) |
| Hesperetin | 303.08551 | 69 (2), 91.05 (1), 97.03 (1), 111.01 (2), 123.04 (2), 125.06 (1), 134.04 (2), 135.04 (4), 137.06 (3), 149.06 (9), 151.04 (1), 151.07 (1), 153.02 (100), 163.04 (11), 177.05 (69), 179.03 (11), 219.06 (1), 285.08 (1), 303.09 (72) |
| Hesperetin 7-O-glucoside | 465.13791 | 67.02 (2), 69 (1), 69.03 (1), 85.03 (2), 121.03 (1), 123.04 (1), 125.06 (1), 137.06 (4), 149.06 (6), 151.04 (1), 151.08 (2), 153.02 (80), 163.04 (9), 177.05 (57), 179.03 (10), 285.07 (1), 303.09 (100), 315.09 (2), 345.1 (1), 369.1 (1) |
| Histamine | 112.08675 | 56.05 (7), 67.03 (3), 68.05 (15), 69.04 (27), 70.07 (100), 95.06 (5), 112.09 (17) |
| Homoeriodictyol | 108.02165 | 108.02 (12), 134.04 (10), 149.06 (1), 177.02 (1), 258.05 (2), 286.05 (10), 301.07 (100) |
| homoeriodictyol 7-O-rutinoside | 609.18256 | 65 (1), 83.01 (1), 107.01 (3), 108.02 (2), 125.02 (4), 134.04 (5), 149.06 (1), 151 (11), 301.07 (100), 325.07 (4), 343.08 (1), 609.18 (6) |
| Homoeriodictyol chalcone | 301.07166 | 108.02 (12), 124.02 (1), 125.02 (6), 134.04 (14), 136.02 (8), 149.06 (2), 151 (22), 152.01 (1), 177.02 (2), 286.05 (12), 301.07 (100) |
| Hypoxanthine | 137.04579 | 82.04 (1), 94.04 (5), 110.03 (10), 119.04 (7), 137.05 (100) |
| Indole-3-acetaldehyde | 160.07549 | 67.04 (1), 105.07 (4), 115.05 (13), 117.06 (7), 118.06 (1), 132.08 (16), 133.06 (3), 142.07 (2), 143.07 (2), 159.07 (2), 160.08 (100) |
| Indole-3-carboxaldehyde | 146.05988 | 91.05 (18), 117.06 (4), 118.07 (100), 146.06 (15) |
| Indole | 118.06495 | 91.05 (22), 117.06 (2), 118.07 (100) |
| Indolin-2-one | 134.05974 | 53.04 (12), 65.04 (3), 79.05 (38), 89.04 (3), 91.05 (49), 104.05 (1), 106.07 (62), 116.05 (28), 132.04 (3), 134.06 (100) |
| Indoxyl | 134.05984 | 65.04 (4), 79.05 (31), 89.04 (2), 91.05 (39), 95.05 (3), 105.03 (4), 106.07 (58), 107.05 (2), 116.05 (23), 132.04 (3), 134.06 (100) |
| IPA imine | 203.08127 | 130.07 (54), 156.07 (1), 157.08 (100), 185.07 (5), 203.08 (65) |
| Ipsdienone | 151.11139 | 53 (1), 53.04 (1), 55.02 (1), 57.07 (2), 65.04 (3), 67.05 (5), 69.07 (1), 79.05 (2), 81.03 (8), 81.07 (7), 91.05 (17), 93.07 (2), 94.04 (6), 95.05 (16), 95.09 (2), 105.07 (2), 107.05 (5), 107.09 (2), 109.06 (37), 109.1 (1), 119.05 (2), 121.06 (1), 123.08 (1), 133.1 (2), 151.11 (100) |
| Isocitric acid | 191.01958 | 57.03 (5), 67.02 (1), 85.03 (27), 87.01 (47), 111.01 (100), 129.02 (6), 173.01 (1), 191.02 (11) |
| Isoglutamine | 145.06181 | 71.01 (3), 72.01 (9), 84.05 (47), 86.02 (9), 98.02 (6), 99.06 (5), 101.07 (26), 109.04 (24), 125.04 (17), 127.05 (100), 128.04 (51), 145.06 (73) |
| Isorhamnetin | 317.06549 | 92.03 (2), 93.03 (2), 121.03 (1), 139.04 (1), 151.04 (1), 153.02 (14), 201.05 (1), 217.05 (2), 229.05 (6), 245.04 (3), 246.05 (2), 257.04 (4), 273.04 (3), 274.05 (6), 285.04 (7), 302.04 (22), 317.07 (100) |
| isorhamnetin 7-O-rutinoside | 625.17731 | 57.03 (14), 69.03 (20), 71.05 (70), 85.03 (100), 127.04 (14), 129.05 (19), 135.04 (13), 137.06 (11), 153.02 (73), 179.03 (13), 191.03 (14), 195.03 (63), 317.07 (61), 377.09 (63) |
| Jasmone | 165.12732 | 53.04 (2), 55.02 (2), 55.05 (5), 57.03 (4), 65.04 (1), 67.05 (29), 69.03 (4), 69.07 (5), 71.05 (2), 79.05 (27), 81.07 (61), 83.05 (7), 83.09 (1), 85.06 (4), 91.05 (32), 93.07 (28), 95.05 (4), 95.09 (17), 97.06 (7), 99.08 (1), 105.07 (54), 107.09 (22), 109.06 (11), 109.1 (3), 117.07 (1), 119.05 (4), 119.09 (42), 121.1 (52), 123.08 (7), 123.12 (8), 137.1 (4), 137.13 (1), 145.1 (1), 147.12 (93), 165.13 (100) |
| Kaempferide | 301.06909 | 55.02 (3), 57.03 (3), 79.05 (5), 91.05 (2), 95.05 (1), 107.05 (2), 111.04 (19), 121.06 (1), 125.06 (1), 153.02 (1), 229.05 (1), 258.05 (36), 286.05 (52), 301.07 (100) |
| kaempferol 7-O-glucoside | 449.1069 | 55.02 (1), 85.03 (1), 109.03 (1), 121.03 (1), 135.04 (3), 137.02 (6), 149.02 (1), 161.02 (2), 163.04 (2), 165.02 (15), 177.02 (1), 179.03 (1), 195.03 (2), 255.07 (1), 286.05 (3), 287.05 (6), 299.05 (100), 311.06 (11), 313.07 (3), 323.06 (1), 325.03 (1), 325.07 (20), 327.05 (1), 329.07 (74), 337.07 (2), 353.07 (39), 383.08 (25), 395.08 (22), 413.09 (21), 431.1 (15), 449.11 (5) |
| L-2-Amino-6-oxoheptanedioate | 190.07092 | 56.05 (3), 59.05 (1), 70.07 (3), 72.08 (4), 84.04 (84), 84.08 (2), 85.03 (1), 87.04 (1), 102.06 (23), 112.08 (3), 118.09 (1), 130.05 (100), 130.09 (2), 144.07 (2) |
| L-2-Aminoadipate | 162.07584 | 55.02 (1), 56.05 (1), 70.07 (18), 84.04 (3), 85.03 (2), 85.06 (1), 98.06 (100), 116.05 (2), 116.07 (35), 131.03 (1), 144.07 (16), 162.08 (10) |
| L-4-Hydroxyglutamate semialdehyde | 146.04587 | 74.02 (1), 85.03 (1), 102.06 (100), 128.04 (38), 146.05 (17) |
| L-Adrenaline | 184.09628 | 55.02 (10), 67.05 (1), 70.03 (1), 79.05 (8), 83.05 (1), 91.05 (3), 95.05 (40), 96.08 (1), 97.06 (1), 105.03 (3), 107.05 (18), 110.1 (1), 120.08 (4), 123.04 (2), 125.06 (1), 138.09 (100), 139.08 (2), 149.06 (10), 166.09 (3), 184.1 (97) |
| L-Allothreonine | 120.06548 | 53.04 (1), 56.05 (70), 57.03 (4), 74.06 (100), 75.04 (1), 84.04 (4), 102.06 (21), 120.07 (5) |
| L-Arginine | 173.10434 | 131.08 (100), 156.08 (2), 173.1 (5) |
| L-Asparagine | 131.04623 | 58.03 (10), 70.03 (54), 71.01 (4), 72.01 (26), 85.04 (1), 86.02 (4), 87.06 (1), 88.04 (1), 96.01 (2), 111.02 (6), 113.04 (59), 114.02 (100), 131.05 (16) |
| L-Aspartate | 132.03024 | 71.01 (17), 72.01 (2), 88.04 (100), 114.02 (4), 115 (22), 132.03 (24) |
| L-Citrulline | 176.1028 | 70.07 (100), 71.05 (1), 71.06 (2), 86.06 (1), 97.08 (1), 113.07 (50), 114.06 (8), 115.09 (9), 116.07 (11), 130.1 (2), 141.07 (1), 159.08 (48) |
| L-Dihydroanticapsin | 202.10713 | 74.02 (7), 85.06 (1), 88.04 (1), 98.06 (1), 100.08 (3), 126.07 (2), 128.07 (100), 138.09 (3), 142.05 (2), 156.08 (3), 156.1 (13), 166.09 (1), 184.1 (3), 202.11 (18) |
| L-Glutamate | 148.06027 | 56.05 (5), 70.07 (18), 71.05 (33), 84.04 (100), 85.03 (2), 88.04 (10), 88.08 (1), 99.04 (2), 102.05 (19), 117.05 (1), 130.05 (10), 148.06 (2) |
| L-Glutamine | 147.07619 | 56.05 (3), 84.04 (100), 85.03 (1), 88.04 (2), 101.07 (4), 102.05 (3), 130.05 (35), 147.08 (1) |
| L-Gulonate | 195.05107 | 59.01 (8), 71.01 (6), 72.99 (10), 75.01 (100), 85.03 (9), 87.01 (15), 129.02 (37), 195.05 (69) |
| L-Histidine | 156.0764 | 82.05 (2), 83.06 (9), 85.08 (4), 93.04 (6), 95.06 (7), 110.07 (100), 156.08 (9) |
| L-Isoleucine | 132.10179 | 69.07 (18), 86.06 (2), 86.1 (100), 132.1 (1) |
| L-Leucine | 132.10187 | 58.07 (5), 68.05 (23), 69.07 (8), 74.02 (2), 84.04 (5), 84.08 (13), 86.06 (50), 86.1 (100), 87.04 (4), 132.1 (8) |
| L-Ornithine | 133.09717 | 60.04 (1), 70.03 (5), 70.07 (17), 74.02 (100), 87.06 (36), 88.04 (9), 116.07 (3) |
| L-Phenylalanine | 164.07169 | 72.01 (40), 147.05 (100), 164.07 (51) |
| L-Proline | 116.07047 | 70.07 (100), 116.07 (11) |
| L-Serine | 104.03532 | 72.01 (86), 74.02 (100), 104.04 (41) |
| L-Threonine | 118.05098 | 72.01 (74), 74.02 (100), 86.02 (3), 118.05 (22) |
| L-Tryptophan | 205.09688 | 58.07 (5), 60.04 (3), 84.04 (1), 94.07 (1), 106.07 (5), 117.06 (1), 118.07 (3), 120.08 (1), 130.06 (1), 132.08 (2), 134.06 (5), 142.07 (2), 144.04 (1), 146.06 (4), 148.08 (4), 159.09 (8), 160.08 (3), 162.05 (9), 170.06 (1), 174.05 (1), 176.07 (4), 177.1 (6), 187.09 (15), 205.1 (100) |
| L-Tyrosine | 182.08098 | 56.05 (8), 91.05 (36), 95.05 (16), 119.05 (41), 123.04 (62), 136.08 (100), 147.04 (20), 165.05 (31) |
| L-Valine | 118.08624 | 58.07 (24), 118.09 (100) |
| Limocitrin 3-O-β-D-glucopyranoside | 507.11411 | 85.03 (6), 302.04 (7), 329.03 (95), 330.04 (18), 344.05 (37), 345.06 (33), 492.09 (5), 507.11 (100) |
| limocitrunshin | 651.1568 | 57.03 (11), 59.01 (2), 99.05 (4), 101.02 (2), 125.02 (3), 148.02 (1), 163.04 (3), 302.04 (12), 305.09 (2), 329.03 (93), 330.04 (58), 344.05 (32), 345.06 (100), 507.11 (89), 549.13 (81), 589.16 (23), 651.16 (15) |
| LL-2,6-Diaminoheptanedioate | 191.10245 | 56.05 (6), 58.07 (1), 59.05 (1), 68.05 (2), 70.03 (3), 70.07 (5), 74.02 (1), 74.06 (10), 84.04 (100), 85.03 (2), 86.06 (3), 88.04 (1), 100.08 (1), 102.06 (3), 110.02 (1), 114.05 (3), 116.07 (2), 117.06 (1), 120.07 (2), 127.09 (5), 128.07 (49), 130.05 (20), 132.07 (2), 145.03 (2), 145.06 (1), 145.1 (1), 156.07 (3), 173.09 (8), 174.08 (46), 191.1 (10) |
| Loganate | 375.12976 | 55.02 (2), 57.03 (13), 59.01 (3), 71.01 (3), 72.99 (1), 75.01 (1), 83.01 (2), 84.02 (3), 85.03 (100), 87.01 (1), 103 (1), 129.02 (11), 357.12 (1), 375.13 (8) |
| Maleamate | 114.01959 | 70.03 (100), 71.01 (44), 114.02 (19) |
| Mandelonitrile | 134.05984 | 79.05 (3), 107.05 (11), 116.05 (1), 133.05 (2), 134.06 (100) |
| Methyl 9,12,15-octadecatrienoate | 293.24698 | 53.04 (1), 55.05 (7), 57.07 (1), 59.05 (1), 65.04 (1), 67.05 (89), 69.07 (10), 79.05 (14), 81.07 (100), 83.05 (1), 83.09 (6), 85.06 (2), 91.05 (5), 93.07 (14), 95.05 (1), 95.09 (86), 97.06 (2), 97.1 (2), 99.08 (1), 105.07 (5), 107.09 (9), 109.06 (1), 109.1 (35), 111.08 (1), 119.09 (4), 121.1 (9), 123.08 (2), 123.12 (19), 133.1 (4), 135.08 (1), 135.12 (6), 137.1 (2), 137.13 (11), 145.1 (5), 147.12 (5), 149.1 (2), 149.13 (5), 151.11 (1), 151.15 (5), 159.12 (4), 161.13 (2), 163.11 (3), 163.15 (3), 165.13 (1), 165.16 (1), 173.13 (6), 177.13 (4), 177.16 (2), 179.14 (1), 187.15 (3), 191.14 (2), 195.14 (1), 201.16 (1), 205.16 (1), 209.15 (2), 219.21 (1), 223.17 (1), 233.23 (1), 243.21 (8), 261.22 (9), 275.2 (1), 293.25 (24) |
| Methyl methanthranilate | 166.08601 | 79.05 (1), 107.05 (2), 120.08 (100), 166.09 (1) |
| Methyl γ-linolenate | 293.24683 | 53.04 (2), 55.05 (10), 57.07 (2), 65.04 (2), 67.05 (92), 69.07 (9), 79.05 (16), 81.07 (100), 83.05 (1), 83.09 (4), 85.06 (1), 91.05 (5), 93.07 (11), 95.09 (71), 97.06 (1), 97.1 (2), 105.07 (3), 107.09 (8), 109.1 (33), 117.07 (1), 119.09 (4), 121.1 (7), 123.12 (18), 131.09 (4), 133.1 (3), 135.12 (4), 137.1 (1), 137.13 (7), 145.1 (3), 147.12 (2), 149.1 (1), 149.13 (2), 151.11 (1), 151.15 (2), 159.12 (3), 161.13 (2), 163.15 (2), 165.13 (1), 173.13 (3), 177.13 (1), 179.14 (1), 187.15 (2), 191.14 (1), 243.21 (3), 261.22 (4), 293.25 (19) |
| Monacolin L acid | 323.22122 | 53.04 (1), 55.02 (5), 55.05 (28), 67.05 (14), 69.03 (4), 69.07 (11), 81.03 (2), 81.07 (15), 83.05 (2), 95.05 (4), 95.09 (6), 97.03 (2), 97.06 (2), 107.05 (3), 109.06 (2), 111.04 (1), 121.1 (3), 133.1 (2), 147.12 (2), 159.12 (1), 189.13 (1), 245.19 (1), 255.17 (2), 263.2 (1), 273.18 (9), 291.2 (100), 305.21 (26), 323.22 (9) |
| Morin | 303.04971 | 69 (4), 97.03 (1), 111.01 (2), 111.04 (1), 123.04 (1), 135.04 (4), 137.02 (1), 137.06 (4), 151.04 (1), 153.02 (100), 163.04 (9), 171.03 (17), 179.03 (11), 229.05 (1), 303.05 (16) |
| Myrcene | 137.13216 | 53.04 (1), 55.05 (5), 57.07 (9), 65.04 (1), 67.05 (24), 69.07 (7), 79.05 (8), 81.07 (68), 91.05 (2), 93.07 (5), 95.09 (100), 109.1 (6), 137.13 (14) |
| N-(L-Arginino)succinate | 291.12982 | 60.06 (3), 70.03 (1), 70.07 (100), 71.05 (3), 72.08 (3), 74.02 (2), 88.04 (17), 98.03 (1), 98.06 (1), 112.05 (1), 112.09 (4), 113.07 (5), 114.06 (1), 115.09 (8), 116.07 (29), 130.1 (1), 131.05 (2), 133.1 (10), 134.04 (4), 141.07 (1), 158.06 (6), 158.09 (8), 175.12 (3), 176.07 (10), 228.1 (4), 246.11 (4), 273.12 (5), 274.1 (2), 291.13 (66) |
| N-Acetyl-L-glutamate 5-semialdehyde | 172.06157 | 57.03 (10), 67.02 (7), 70.03 (4), 72.01 (3), 74.02 (18), 84.05 (23), 86.06 (5), 98.02 (5), 112.04 (14), 116.04 (5), 126.06 (4), 128.07 (100), 130.05 (26), 172.06 (97) |
| N-Acetyl-L-glutamate | 188.05666 | 58.03 (10), 59.01 (35), 74.02 (1), 82.03 (1), 84.05 (2), 98.02 (1), 98.06 (1), 100.04 (3), 100.08 (24), 102.06 (100), 126.06 (6), 128.04 (86), 142.05 (1), 144.07 (27), 146.05 (8), 170.05 (19), 188.06 (24) |
| N-Acetyl-L-phenylalanine | 206.08218 | 58.03 (100), 59.01 (2), 164.07 (26), 206.08 (24) |
| N-Acetylornithine | 175.10791 | 70.07 (100), 71.05 (1), 72.08 (5), 115.09 (1), 116.07 (29) |
| N-Methylserotonin | 191.11755 | 132.08 (6), 133.06 (1), 134.06 (1), 142.07 (1), 148.08 (17), 160.08 (100) |
| N-Methyltryptamine | 175.12251 | 70.07 (100), 72.08 (4), 91.05 (1), 117.07 (3), 132.08 (18), 143.07 (2), 144.08 (88) |
| N-Methyltyramine | 152.10666 | 91.05 (5), 93.07 (10), 103.05 (6), 121.06 (100), 152.11 (1) |
| N-Succinyl-2-L-amino-6-oxoheptanedioate | 290.08673 | 56.05 (3), 57.03 (3), 60.04 (3), 61.03 (2), 69 (4), 69.03 (2), 70.07 (2), 73.03 (2), 74.06 (1), 81.03 (3), 84.04 (100), 85.03 (16), 87.01 (7), 96.04 (2), 97.03 (4), 98.06 (4), 99.04 (1), 103.04 (15), 109.03 (3), 126.05 (25), 127.04 (3), 129.02 (5), 144.07 (1), 147.03 (3), 156.07 (1), 168.07 (8), 171.03 (1), 186.08 (2), 212.05 (1), 228.09 (1), 272.08 (3), 290.09 (15) |
| N~2~-(1-Carboxyethyl)lysine | 219.13382 | 58.07 (4), 60.04 (100), 86.1 (69), 128.11 (2), 132.1 (52), 173.13 (28), 201.12 (5), 219.13 (10) |
| N2-Succinyl-L-ornithine | 233.1131 | 55.02 (4), 55.05 (5), 56.05 (1), 69.03 (2), 70.07 (100), 71.05 (8), 72.08 (43), 73.03 (7), 74.02 (1), 84.04 (9), 84.08 (3), 86.06 (3), 87.04 (3), 88.04 (1), 88.08 (2), 97.08 (4), 98.06 (3), 100.04 (2), 101.02 (20), 104.07 (3), 115.09 (65), 116.07 (14), 118.05 (2), 124.08 (3), 127.09 (2), 128.07 (3), 130.05 (4), 133.1 (4), 145.03 (3), 152.07 (9), 169.1 (4), 170.08 (4), 173.09 (1), 187.11 (1), 197.09 (45), 198.08 (8), 215.1 (8), 216.09 (13), 233.11 (20) |
| N6-Acetyl-L-lysine | 189.1232 | 70.07 (1), 85.03 (2), 86.1 (100), 132.1 (40), 143.12 (23), 189.12 (1) |
| N6-Acetyl-N6-hydroxy-L-lysine | 203.10349 | 71.01 (29), 74.02 (19), 88.04 (20), 97.03 (51), 100.04 (19), 114.06 (7), 203.1 (100) |
| Neohesperidin | 611.19379 | 57.03 (7), 69.03 (10), 71.05 (39), 75.04 (2), 85.03 (49), 107.05 (1), 111.04 (2), 127.04 (1), 129.05 (12), 137.06 (2), 149.06 (4), 151.04 (2), 151.07 (2), 153.02 (52), 177.05 (39), 179.03 (5), 301.07 (4), 303.09 (100), 315.09 (5), 327.09 (4), 345.1 (11), 369.1 (11), 411.11 (1), 413.12 (11), 429.12 (3), 431.13 (4) |
| Nerolidyl propionate | 279.23178 | 53 (4), 53.04 (3), 55.02 (3), 55.05 (13), 57.03 (2), 57.07 (6), 65.04 (3), 67.05 (80), 69.07 (16), 71.05 (2), 71.09 (62), 79.05 (23), 81.07 (100), 83.05 (4), 83.09 (13), 85.06 (4), 85.1 (2), 91.05 (19), 93.07 (21), 95.09 (77), 97.06 (6), 97.1 (6), 99.08 (58), 105.07 (17), 107.09 (14), 109.1 (24), 111.08 (2), 113.1 (2), 119.09 (13), 121.1 (12), 123.08 (2), 123.12 (12), 125.1 (2), 127.11 (3), 131.09 (9), 133.1 (4), 135.12 (9), 137.13 (11), 139.11 (3), 145.1 (6), 147.12 (42), 149.1 (3), 149.13 (5), 151.11 (2), 151.15 (5), 153.13 (8), 159.12 (3), 161.13 (3), 163.11 (8), 163.15 (11), 165.13 (23), 167.14 (4), 173.13 (7), 177.16 (2), 181.16 (7), 191.14 (2), 195.14 (3), 209.15 (5), 223.17 (2), 243.21 (10), 251.24 (3), 261.22 (18), 279.23 (80) |
| Nobiletin | 403.13818 | 165.06 (3), 183.03 (11), 211.02 (5), 327.09 (10), 345.1 (2), 358.07 (5), 373.09 (90), 388.11 (9), 403.14 (100) |
| O-beta-D-Glucosylzeatin | 380.15604 | 58.01 (1), 59.01 (6), 71.01 (27), 72.99 (1), 85.03 (1), 89.02 (8), 101.02 (6), 113.02 (3), 119.03 (2), 161.05 (1), 362.15 (1), 380.16 (100) |
| o-Ethylbenzaldehyde | 135.08028 | 65.04 (4), 79.05 (8), 91.05 (37), 93.07 (3), 105.07 (21), 106.04 (1), 107.05 (2), 107.09 (100), 135.08 (6) |
| o-Isopropenyltoluene | 133.10098 | 53.04 (1), 77.04 (1), 79.05 (4), 91.05 (12), 93.07 (7), 103.05 (3), 105.07 (100), 115.05 (2), 117.07 (2), 131.09 (4), 133.1 (36) |
| Obacunone | 455.20569 | 67.05 (12), 69.07 (10), 79.05 (16), 81.03 (18), 95.01 (40), 95.05 (24), 107.05 (10), 121.06 (12), 151.11 (9), 161.06 (33), 175.08 (17), 215.11 (16), 243.1 (24), 351.2 (12), 367.19 (13), 393.21 (23), 395.19 (24), 409.2 (16), 411.22 (12), 419.19 (10), 437.2 (22), 455.21 (100) |
| Paeoniflorin | 479.15588 | 55.02 (2), 57.03 (16), 71.01 (4), 72.99 (3), 75.01 (1), 83.01 (5), 84.02 (2), 85.03 (100), 87.01 (1), 101.02 (1), 121.03 (20), 129.02 (55) |
| Pantothenate | 218.10327 | 59.01 (1), 71.01 (10), 71.05 (12), 88.04 (100), 99.05 (5), 146.08 (48), 218.1 (23) |
| Pentalenene | 205.19463 | 53.04 (2), 55.05 (23), 57.07 (7), 67.05 (21), 69.07 (36), 81.07 (59), 83.09 (17), 93.07 (61), 95.09 (56), 97.1 (12), 107.09 (66), 109.1 (51), 111.12 (5), 119.09 (3), 121.1 (100), 123.12 (26), 135.12 (53), 137.13 (3), 147.12 (1), 149.13 (82), 163.15 (13), 205.19 (81) |
| Perillic acid | 167.1066 | 53.04 (7), 55.02 (3), 55.05 (15), 57.07 (5), 67.05 (9), 69.03 (12), 69.07 (3), 71.05 (3), 79.05 (58), 81.07 (43), 83.05 (17), 93.07 (42), 95.05 (5), 95.09 (6), 97.03 (19), 97.06 (18), 105.07 (10), 107.05 (7), 107.09 (6), 109.07 (3), 111.04 (100), 113.06 (4), 121.07 (22), 121.1 (33), 125.06 (7), 139.11 (3), 149.1 (32), 167.11 (87) |
| Phenylacetaldehyde | 121.0646 | 51.02 (1), 65.04 (1), 77.04 (1), 91.05 (53), 93.07 (53), 95.05 (12), 103.05 (73), 121.06 (100) |
| Pheophorbide a | 593.2746 | 533.25 (37), 593.27 (100) |
| Phlorin | 289.0918 | 59.05 (56), 69.03 (49), 81.03 (24), 85.03 (16), 87.01 (3), 87.04 (100), 97.03 (10), 99.04 (10), 109.03 (8), 125.02 (3), 127.04 (36), 143.03 (13), 145.05 (3), 213.04 (5), 231.05 (4), 271.08 (12), 289.09 (9) |
| Piperideine | 84.08069 | 55.05 (1), 56.05 (17), 67.05 (1), 69.06 (1), 82.07 (4), 84.08 (100) |
| Piperidine | 86.09633 | 57.06 (15), 58.07 (22), 68.05 (78), 69.07 (52), 86.1 (100) |
| Plastoquinol-1 | 207.13763 | 53.04 (4), 55.05 (3), 57.07 (11), 65.04 (8), 67.05 (6), 69.03 (7), 69.07 (5), 91.05 (97), 105.07 (33), 107.05 (4), 107.09 (68), 109.06 (21), 119.09 (27), 121.06 (25), 123.08 (32), 131.09 (20), 133.1 (5), 135.08 (4), 137.06 (5), 145.1 (6), 147.08 (6), 149.1 (88), 151.08 (74), 161.13 (10), 165.09 (6), 189.13 (50), 207.14 (100) |
| Pseudoecgonine | 186.11238 | 67.05 (1), 73.03 (1), 80.05 (3), 95.09 (11), 122.1 (42), 126.05 (7), 140.11 (19), 168.1 (40), 186.11 (100) |
| Pseudouridine | 245.07661 | 57.03 (3), 69.03 (5), 70.03 (10), 73.03 (3), 84.04 (15), 113.03 (100), 115.04 (3), 133.05 (4) |
| Psilocin | 205.13345 | 58.07 (100), 132.08 (4), 160.08 (67), 205.13 (5) |
| Pyridoxine | 170.08098 | 55.02 (2), 57.03 (2), 69.03 (11), 73.03 (4), 81.03 (3), 82.07 (3), 84.04 (2), 85.03 (2), 87.04 (2), 97.03 (3), 110.06 (3), 114.06 (2), 128.07 (6), 152.07 (8), 170.08 (100) |
| Quercetin 3-O-beta-D-glucosyl-(1-2)-beta-D-glucoside | 625.14142 | 107.01 (3), 125.02 (12), 151 (9), 152.01 (6), 179 (8), 255.03 (26), 271.02 (61), 300.03 (100), 301.04 (17), 625.14 (72) |
| Quercetin | 303.04938 | 69 (2), 109.03 (1), 111.01 (1), 121.03 (2), 137.02 (7), 153.02 (10), 163.04 (1), 165.02 (3), 187.04 (1), 201.05 (4), 229.05 (9), 247.06 (1), 257.04 (5), 274.05 (1), 285.04 (2), 303.05 (100) |
| Riboflavin | 377.14505 | 57.03 (6), 61.03 (3), 69.03 (12), 71.01 (1), 71.05 (2), 73.03 (1), 75.04 (2), 81.03 (3), 99.04 (5), 117.05 (1), 172.09 (30), 200.08 (3), 243.09 (67), 359.13 (2), 377.15 (100) |
| Rosmarinate | 361.09094 | 151.04 (5), 164.05 (1), 243.07 (2), 317.07 (1), 361.09 (100) |
| Santaflavone | 359.11182 | 149.06 (3), 151.04 (3), 153.02 (10), 181.01 (5), 273.08 (1), 301.07 (14), 314.04 (10), 315.09 (1), 329.07 (84), 343.08 (1), 344.09 (16), 359.11 (100) |
| Scolymoside | 593.15149 | 117.03 (5), 356.05 (1), 413.09 (2), 473.11 (14), 503.12 (3), 575.14 (1), 593.15 (100) |
| Scopoletin | 193.04951 | 122.04 (4), 133.03 (35), 133.06 (1), 137.06 (10), 149.06 (2), 150.03 (3), 165.05 (3), 178.03 (22), 193.05 (100) |
| Sedoheptulose | 209.06654 | 55.02 (1), 57.03 (12), 59.01 (8), 69 (1), 71.01 (26), 72.99 (8), 75.01 (5), 83.01 (2), 85.03 (100), 87.01 (4), 89.02 (8), 101.02 (1), 103 (2), 111.01 (4), 115 (2), 129.02 (7), 133.01 (10), 147.03 (4), 173.01 (1), 209.07 (1) |
| Sinapate | 225.07529 | 65.04 (3), 77.04 (2), 79.05 (2), 91.05 (45), 99.04 (3), 103.05 (1), 105.03 (2), 107.05 (1), 109.06 (2), 111.04 (1), 118.04 (4), 119.05 (51), 135.04 (2), 147.04 (39), 149.02 (5), 151.04 (3), 164.05 (3), 175.04 (71), 177.02 (2), 179.07 (5), 183.06 (1), 192.04 (26), 207.07 (100) |
| Sinensetin | 373.12747 | 125.02 (1), 148.05 (1), 153.02 (14), 163.08 (6), 165.05 (4), 181.01 (6), 287.09 (1), 313.07 (1), 314.08 (1), 315.09 (13), 328.06 (2), 329.1 (2), 342.07 (1), 343.08 (79), 357.1 (8), 358.1 (6), 373.13 (100) |
| Skatole | 132.08061 | 91.05 (2), 105.07 (6), 117.06 (38), 130.07 (5), 131.07 (3), 132.08 (100) |
| Spermidine | 146.16502 | 58.07 (8), 72.08 (100), 75.09 (4), 84.08 (9), 112.11 (24), 129.14 (6), 146.17 (3) |
| Stearidonic acid | 277.21613 | 53 (2), 53.04 (5), 55.05 (14), 57.07 (2), 57.07 (34), 67.05 (33), 69.07 (15), 71.05 (2), 71.09 (14), 79.05 (51), 81.03 (2), 81.07 (41), 83.05 (2), 83.09 (8), 91.05 (21), 93.07 (80), 95.05 (4), 95.09 (28), 97.06 (3), 97.1 (3), 99.08 (10), 105.07 (17), 107.05 (3), 107.09 (49), 109.06 (2), 109.1 (11), 117.07 (2), 119.09 (10), 121.1 (31), 123.12 (2), 131.09 (3), 133.1 (7), 135.12 (45), 137.1 (2), 145.1 (3), 147.12 (6), 149.13 (16), 151.11 (8), 159.12 (3), 161.13 (7), 163.11 (4), 163.15 (5), 167.11 (2), 173.13 (2), 177.13 (3), 179.11 (20), 179.14 (19), 195.14 (2), 213.16 (1), 217.16 (2), 219.14 (3), 221.15 (4), 235.17 (89), 259.21 (5), 277.22 (100) |
| Styrene | 105.06969 | 51.02 (1), 53.04 (15), 77.04 (1), 79.05 (35), 103.05 (21), 105.07 (100) |
| Succinate semialdehyde | 101.02435 | 55.02 (35), 57.03 (41), 59.01 (31), 71.01 (100), 101.02 (67) |
| Succinate | 117.01921 | 59.01 (39), 73.03 (100), 99.01 (13), 117.02 (22) |
| Sudachiin C | 665.17297 | 57.03 (20), 59.01 (8), 71.01 (2), 99.05 (13), 101.02 (4), 125.02 (7), 161.05 (3), 305.09 (7), 344.05 (23), 358.07 (67), 359.08 (100), 521.13 (94), 563.14 (61), 603.17 (17) |
| Syringetin | 347.07587 | 121.03 (1), 151.04 (1), 245.04 (2), 261.04 (6), 273.04 (7), 287.05 (2), 300.03 (4), 301.03 (16), 303.05 (1), 315.05 (4), 317.03 (2), 331.05 (2), 332.05 (51), 347.08 (100) |
| tamarixetin 7-rutinoside | 623.16187 | 57.03 (1), 65 (1), 107.01 (1), 125.02 (5), 150.03 (1), 151 (5), 152.01 (1), 165.06 (1), 272.03 (3), 285.04 (6), 287.06 (2), 300.03 (39), 314.04 (35), 315.05 (100), 623.16 (75) |
| Testosterone | 289.21582 | 53 (1), 53.04 (3), 55.02 (1), 55.05 (23), 57.03 (1), 57.07 (3), 67.05 (11), 69.03 (3), 69.07 (10), 71.05 (1), 79.05 (18), 81.07 (18), 83.05 (2), 85.06 (1), 91.05 (18), 93.07 (23), 95.05 (2), 95.09 (12), 97.06 (1), 105.07 (34), 107.05 (1), 107.09 (21), 109.06 (8), 109.1 (4), 111.08 (1), 119.09 (26), 121.1 (7), 123.08 (4), 135.08 (13), 145.1 (11), 159.12 (7), 163.11 (11), 173.13 (4), 175.15 (4), 185.13 (1), 189.13 (2), 191.14 (4), 203.14 (1), 215.14 (2), 233.15 (2), 243.21 (3), 247.17 (1), 253.19 (2), 261.22 (24), 271.21 (8), 289.22 (100) |
| Tetramethyl-O-scutellarein | 343.1171 | 132.06 (1), 133.06 (3), 135.04 (2), 153.02 (6), 168.04 (1), 181.01 (2), 253.08 (2), 267.07 (2), 269.08 (1), 281.08 (5), 282.09 (13), 284.07 (2), 285.08 (10), 297.08 (14), 299.09 (16), 310.08 (4), 311.09 (3), 313.07 (42), 327.09 (10), 328.09 (6), 343.12 (100) |
| Threonate | 135.02974 | 59.01 (7), 71.01 (8), 72.99 (21), 75.01 (100), 87.01 (18), 89.02 (9), 117.02 (1), 135.03 (30) |
| trans-Aconitate | 173.00897 | 85.03 (100), 111.01 (18), 129.02 (12), 173.01 (1) |
| trans-beta-D-Glucosyl-2-hydroxycinnamate | 325.09302 | 163.04 (100) |
| trans-Isoferulic acid | 195.0648 | 91.05 (1), 103.05 (1), 106.04 (1), 117.03 (42), 121.06 (1), 134.04 (6), 135.04 (5), 145.03 (71), 149.06 (20), 162.03 (2), 163.04 (8), 177.05 (100), 195.07 (1) |
| Traumatin | 213.14803 | 53.04 (1), 55.02 (11), 55.05 (56), 57.03 (1), 57.07 (1), 59.05 (1), 67.05 (22), 69.03 (6), 69.07 (11), 71.05 (55), 73.06 (1), 79.05 (14), 81.03 (1), 81.07 (21), 83.05 (4), 83.09 (34), 85.06 (5), 87.04 (7), 93.07 (13), 95.09 (8), 97.06 (78), 97.1 (50), 101.06 (1), 101.1 (1), 105.07 (2), 107.09 (18), 109.06 (1), 109.1 (12), 111.08 (2), 121.06 (1), 121.1 (2), 125.1 (33), 135.08 (4), 143.11 (1), 149.13 (4), 153.09 (1), 177.13 (5), 195.14 (39), 213.15 (100) |
| Tricetin | 303.04938 | 69 (1), 111.01 (1), 121.03 (2), 137.02 (7), 153.02 (10), 163.04 (1), 229.05 (8), 247.06 (1), 257.04 (4), 274.05 (1), 285.04 (2), 303.05 (100) |
| Tricetin pentamethyl ether | 373.12766 | 151.04 (3), 162.07 (1), 163.08 (3), 165.05 (2), 267.06 (1), 268.07 (1), 269.08 (1), 283.1 (1), 285.08 (1), 295.06 (1), 296.07 (3), 297.08 (2), 299.05 (1), 311.09 (5), 312.1 (10), 313.07 (4), 314.08 (2), 315.09 (10), 327.09 (12), 328.06 (1), 329.1 (13), 340.09 (3), 341.1 (2), 342.07 (1), 343.08 (38), 357.1 (13), 358.1 (4), 373.13 (100) |
| Trichodiene | 205.19511 | 55.05 (16), 57.07 (4), 67.05 (8), 69.07 (23), 79.05 (9), 81.07 (26), 83.09 (23), 93.07 (54), 95.09 (36), 97.1 (10), 105.07 (7), 107.09 (52), 109.1 (29), 121.1 (52), 123.12 (14), 135.12 (29), 149.13 (100), 163.15 (6), 205.2 (61) |
| Tylactone | 395.27921 | 55.02 (1), 55.05 (6), 57.03 (1), 57.07 (1), 67.05 (7), 69.07 (6), 71.05 (1), 75.04 (1), 79.05 (6), 81.07 (11), 83.05 (1), 83.09 (2), 93.07 (8), 95.09 (8), 97.06 (1), 97.1 (1), 107.09 (6), 109.06 (8), 109.1 (2), 111.08 (1), 121.1 (5), 123.08 (38), 135.12 (2), 137.1 (3), 147.12 (2), 163.11 (1), 179.14 (13), 205.16 (2), 219.17 (2), 235.17 (1), 247.17 (5), 275.24 (3), 303.23 (43), 321.24 (1), 339.22 (3), 395.28 (100) |
| Tyramine | 138.09122 | 55.02 (1), 65.04 (4), 67.05 (4), 91.05 (12), 93.07 (15), 95.05 (3), 103.05 (9), 121.06 (100), 138.09 (5) |
| Umbelliferone | 163.03871 | 89.04 (64), 91.05 (1), 95.05 (1), 107.05 (13), 135.04 (100), 145.03 (41), 163.04 (89) |
| Umhengerin | 389.12228 | 121.03 (1), 149.02 (1), 165.05 (2), 283.06 (1), 285.08 (5), 297.08 (1), 298.05 (1), 299.09 (2), 313.07 (20), 325.07 (1), 327.09 (2), 328.09 (5), 331.08 (31), 342.07 (1), 343.08 (1), 356.09 (18), 359.08 (8), 373.09 (2), 374.1 (8), 389.12 (100) |
| Uracil | 113.03443 | 70.03 (51), 95.02 (4), 96.01 (34), 113.03 (100) |
| UWM6 | 343.11697 | 243.07 (1), 257.08 (1), 283.06 (1), 284.07 (1), 285.08 (19), 299.09 (1), 313.07 (87), 327.09 (2), 328.09 (7), 343.12 (100) |
| Valylproline | 215.13893 | 55.05 (11), 56.05 (3), 58.07 (1), 69.03 (2), 70.07 (40), 72.08 (75), 73.06 (1), 74.06 (2), 84.04 (4), 84.08 (1), 101.07 (1), 116.07 (100), 169.13 (1), 187.14 (2), 215.14 (12) |
| veronicastroside | 593.15118 | 593.15 (100) |
| Versalide | 259.20532 | 55.05 (7), 57.07 (1), 67.05 (7), 69.07 (24), 83.09 (7), 97.1 (1), 131.09 (9), 147.08 (1), 173.13 (8), 189.13 (3), 201.16 (18), 203.14 (8), 215.18 (4), 241.19 (15), 259.21 (100) |
| Vitexicarpin | 375.10669 | 137.02 (1), 150.03 (1), 165.05 (2), 191.07 (1), 196.04 (1), 286.08 (27), 298.05 (1), 299.06 (2), 314.08 (29), 315.05 (1), 317.07 (3), 329.07 (1), 331.08 (1), 342.07 (19), 345.06 (2), 359.08 (5), 360.08 (23), 375.11 (100) |
| Vitexin 2''-O-beta-D-glucoside | 595.16479 | 55.02 (1), 57.03 (2), 61.03 (1), 73.03 (1), 85.03 (6), 93.03 (1), 103.04 (2), 109.03 (2), 119.05 (8), 121.03 (36), 145.03 (4), 147.04 (1), 161.02 (1), 163.04 (2), 253.05 (1), 271.06 (1), 295.06 (86), 313.07 (6), 325.07 (100), 355.08 (19), 379.08 (56), 397.09 (1), 427.1 (36), 457.11 (85), 475.12 (17), 499.12 (17), 529.13 (8), 541.13 (24), 559.14 (19), 577.15 (9), 595.17 (1) |
| Xanthine | 153.04034 | 55.03 (3), 82.04 (3), 83.02 (1), 98.03 (1), 110.03 (56), 125.05 (4), 136.01 (2), 153.04 (100) |
| α-Glyceryl linolenate | 353.26764 | 53.04 (4), 55.05 (14), 57.03 (5), 57.07 (3), 65.04 (5), 67.05 (87), 69.07 (15), 79.05 (44), 81.07 (95), 83.09 (7), 93.07 (63), 95.05 (3), 95.09 (63), 97.06 (2), 97.1 (2), 107.09 (41), 109.1 (33), 111.08 (3), 119.09 (12), 121.1 (31), 123.12 (21), 133.1 (13), 135.12 (22), 137.13 (6), 145.1 (18), 147.12 (9), 149.13 (19), 159.12 (13), 161.13 (11), 163.15 (14), 173.13 (16), 175.15 (3), 177.16 (5), 187.15 (8), 233.22 (3), 243.21 (22), 261.22 (100), 279.23 (4), 335.26 (6), 353.27 (37) |
| α-Sinensal | 219.17406 | 53.04 (1), 55.05 (14), 57.03 (2), 57.07 (3), 65.04 (2), 67.05 (14), 69.07 (9), 71.05 (2), 79.05 (11), 81.07 (29), 83.05 (1), 83.09 (7), 91.05 (15), 93.07 (36), 95.05 (4), 95.09 (28), 97.06 (9), 97.1 (2), 105.07 (25), 107.09 (76), 108.09 (2), 109.06 (5), 109.1 (17), 111.08 (4), 119.09 (27), 121.06 (5), 121.1 (20), 123.08 (7), 123.12 (8), 125.1 (3), 130.08 (2), 131.09 (11), 133.1 (10), 135.08 (6), 135.12 (37), 137.1 (3), 145.1 (26), 147.12 (16), 149.1 (12), 149.13 (3), 159.12 (19), 161.13 (23), 163.11 (11), 163.15 (3), 173.13 (6), 175.15 (4), 177.13 (3), 191.18 (7), 201.16 (28), 219.17 (100) |
| β-Sinensal | 219.17412 | 67.05 (10), 69.07 (8), 79.05 (7), 81.07 (16), 91.05 (16), 93.07 (21), 95.09 (29), 105.07 (50), 107.05 (6), 107.09 (24), 109.1 (9), 117.07 (8), 119.09 (40), 121.1 (10), 131.09 (17), 133.1 (14), 135.12 (7), 145.1 (36), 149.13 (5), 159.12 (42), 161.13 (100), 163.11 (6), 173.13 (9), 177.13 (5), 201.16 (77), 219.17 (74) |

**Table S4.**

| Pathway ID | Pathway name | Compound Hits | Compounds in pathway | p-value | Compound KEGG ID |
| --- | --- | --- | --- | --- | --- |
| cic01230 | Biosynthesis of amino acids | 23 | 128 | 4.53E-12 | C00025, C00026, C00049, C00062, C00064, C00065, C00077, C00082, C00108, C00123, C00135, C00152, C00158, C00183, C00311, C00327, C00407, C00666, C00956, C01179, C03406, C03871, C04462 |
| cic00470 | D-Amino acid metabolism | 13 | 60 | 2.59E-08 | C00025, C00026, C00049, C00062, C00064, C00065, C00077, C00135, C00433, C00666, C00792, C03239, C03771 |
| cic02010 | ABC transporters | 15 | 97 | 2.49E-07 | C00025, C00031, C00049, C00062, C00064, C00065, C00077, C00123, C00135, C00181, C00183, C00212, C00387, C00407, C01606 |
| cic00220 | Arginine biosynthesis | 8 | 23 | 2.66E-07 | C00025, C00026, C00049, C00062, C00064, C00077, C00327, C03406 |
| cic00970 | Aminoacyl-tRNA biosynthesis | 11 | 52 | 4.20E-07 | C00025, C00049, C00062, C00064, C00065, C00082, C00123, C00135, C00152, C00183, C00407 |
| cic00592 | alpha-Linolenic acid metabolism | 9 | 33 | 4.88E-07 | C01226, C04672, C04780, C16300, C16311, C16321, C16324, C16325, C21924 |
| cic00250 | Alanine, aspartate and glutamate metabolism | 7 | 28 | 1.82E-05 | C00025, C00026, C00049, C00064, C00152, C00158, C03406 |
| cic00460 | Cyanoamino acid metabolism | 8 | 45 | 6.47E-05 | C00049, C00065, C00082, C00152, C00183, C00407, C00561, C02512 |
| cic01210 | 2-Oxocarboxylic acid metabolism | 13 | 128 | 0.00016682 | C00025, C00026, C00049, C00077, C00082, C00123, C00158, C00183, C00311, C00407, C00417, C00956, C01179 |
| cic00053 | Ascorbate and aldarate metabolism | 8 | 55 | 0.00027998 | C00026, C00433, C00679, C00684, C00800, C00818, C00879, C01620 |
| cic00020 | Citrate cycle (TCA cycle) | 5 | 20 | 0.00030783 | C00026, C00149, C00158, C00311, C00417 |
| cic00591 | Linoleic acid metabolism | 5 | 20 | 0.00030783 | C07289, C07354, C14762, C14765, C14825 |
| cic00330 | Arginine and proline metabolism | 9 | 72 | 0.00037688 | C00025, C00062, C00077, C01877, C02647, C03415, C03771, C05938, C10497 |
| cic00660 | C5-Branched dibasic acid metabolism | 6 | 34 | 0.00057501 | C00025, C00026, C00417, C01109, C02341, C06030 |
| cic00630 | Glyoxylate and dicarboxylate metabolism | 8 | 61 | 0.0005771 | C00025, C00026, C00064, C00065, C00149, C00158, C00311, C00417 |
| cic00300 | Lysine biosynthesis | 6 | 35 | 0.00067617 | C00026, C00049, C00666, C00956, C03871, C04462 |
| cic00310 | Lysine degradation | 7 | 50 | 0.00086036 | C00026, C00956, C02727, C03087, C03239, C04020, C05548 |
| cic00944 | Flavone and flavonol biosynthesis | 7 | 51 | 0.00097131 | C00389, C04293, C04443, C04444, C10098, C11620, C12633 |
| cic00770 | Pantothenate and CoA biosynthesis | 5 | 30 | 0.00219403 | C00049, C00106, C00183, C01088, C02642 |
| cic00999 | Biosynthesis of various plant secondary metabolites | 7 | 60 | 0.00255756 | C00049, C00082, C00590, C01752, C05851, C09315, C20417 |
| cic00340 | Histidine metabolism | 6 | 47 | 0.00327759 | C00025, C00026, C00049, C00135, C00388, C16673 |
| cic00261 | Monobactam biosynthesis | 5 | 35 | 0.00440409 | C00049, C00062, C00065, C00082, C01179 |
| cic00040 | Pentose and glucuronate interconversions | 6 | 58 | 0.00930669 | C00026, C00181, C00433, C00502, C00514, C00800 |
| cic00940 | Phenylpropanoid biosynthesis | 6 | 58 | 0.00930669 | C00082, C00590, C00811, C01494, C04366, C10945 |
| cic00350 | Tyrosine metabolism | 7 | 78 | 0.01092848 | C00082, C00483, C00811, C01179, C01850, C02442, C05587 |
| cic00402 | Benzoxazinoid biosynthesis | 2 | 6 | 0.01362695 | C12312, C15769 |
| cic01100 | Metabolic pathways | 105 | 2919 | 0.01476385 | C00025, C00026, C00031, C00049, C00062, C00064, C00065, C00077, C00082, C00106, C00108, C00123, C00135, C00147, C00149, C00152, C00158, C00170, C00181, C00183, C00212, C00257, C00261, C00262, C00311, C00327, C00380, C00387, C00388, C00389, C00407, C00417, C00433, C00483, C00499, C00502, C00514, C00556, C00561, C00590, C00601, C00637, C00666, C00679, C00684, C00792, C00800, C00811, C00818, C00879, C00880, C00956, C01092, C01179, C01226, C01412, C01494, C01546, C01575, C01596, C01606, C01620, C01841, C01850, C01877, C01909, C02076, C02106, C02222, C02341, C02442, C02505, C02512, C02642, C02647, C02727, C03087, C03239, C03406, C03415, C03479, C03519, C03771, C03871, C04462, C04672, C04780, C05519, C05548, C05587, C05938, C06144, C06181, C06213, C08301, C10497, C14825, C17366, C19972, C20417, C20424, C20851, C20853, C20926, C21124 |
| cic00410 | beta-Alanine metabolism | 4 | 31 | 0.0153976 | C00049, C00106, C00135, C02642 |
| cic01200 | Carbon metabolism | 8 | 114 | 0.02659928 | C00025, C00026, C00049, C00065, C00149, C00158, C00257, C00311 |
| cic00290 | Valine, leucine and isoleucine biosynthesis | 3 | 23 | 0.03427242 | C00123, C00183, C00407 |
| cic00710 | Carbon fixation in photosynthetic organisms | 3 | 23 | 0.03427242 | C00049, C00149, C02076 |
